# Separators and Membranes for Advanced Alkaline Water Electrolysis

**DOI:** 10.1021/acs.chemrev.3c00694

**Published:** 2024-04-26

**Authors:** Dirk Henkensmeier, Won-Chul Cho, Patric Jannasch, Jelena Stojadinovic, Qingfeng Li, David Aili, Jens Oluf Jensen

**Affiliations:** † Hydrogen · Fuel Cell Research Center, Korea Institute of Science and Technology, Seoul 02792, Republic of Korea; ‡ Division of Energy & Environment Technology, KIST School, University of Science and Technology, Seoul 02792, Republic of Korea; § KU-KIST Green School, Korea University, Seoul 02841, Republic of Korea; ∥ Department of Future Energy Convergence, Seoul National University of Science & Technology, 232 Gongreung-ro, Nowon-gu, Seoul 01811, Korea; ⊥ Polymer & Materials Chemistry, Department of Chemistry, Lund University, 221 00 Lund, Sweden; # MEMBRASENZ, EPFL Innovation Park Building C, 1015 Lausanne, Switzerland; g Department of Energy Conversion and Storage, Technical University of Denmark (DTU), Fysikvej 310, 2800 Kgs. Lyngby, Denmark

## Abstract

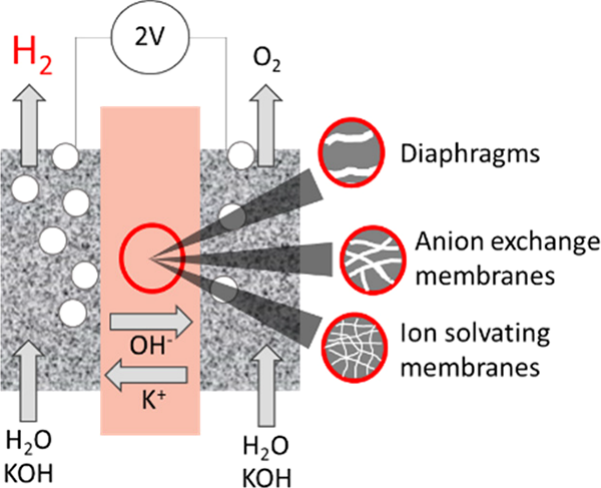

Traditionally, alkaline water electrolysis (AWE) uses diaphragms to separate anode and cathode and is operated with 5–7 M KOH feed solutions. The ban of asbestos diaphragms led to the development of polymeric diaphragms, which are now the state of the art material. A promising alternative is the ion solvating membrane. Recent developments show that high conductivities can also be obtained in 1 M KOH. A third technology is based on anion exchange membranes (AEM); because these systems use 0–1 M KOH feed solutions to balance the trade-off between conductivity and the AEM’s lifetime in alkaline environment, it makes sense to treat them separately as AEM WE. However, the lifetime of AEM increased strongly over the last 10 years, and some electrode-related issues like oxidation of the ionomer binder at the anode can be mitigated by using KOH feed solutions. Therefore, AWE and AEM WE may get more similar in the future, and this review focuses on the developments in polymeric diaphragms, ion solvating membranes, and AEM.

## 1 Introduction

In 2015, 196 parties signed the Paris agreement, which aims to limit global warming to <2 °C, preferably 1.5 °C, compared to preindustrial levels.^1^ In the following years, many countries announced goals to become carbon-neutral between 2030 and 2070, with 2050 being the average and most often announced goal over 69 countries. To achieve these goals, societies need to transition from fossil fuel-based economies to renewable energy based economies. Expanding the use of renewable energy sources will increase the self-sufficiency of almost all countries, but it is also clear that not all countries will have enough readily available locations to install the needed number of wind turbines and solar panels, and some countries like Germany or Korea already prepare to import renewable energy. Furthermore, even if a country can reach self-sufficiency, it will need to store large amounts of energy to compensate for the low production on days with calm winds or shorter days during winter. It is generally believed that transport over long distances and large-scale energy storage are best accomplished by using hydrogen as an energy carrier, either directly as compressed gas or in the form of hydrogen-rich molecules (liquid organic hydrogen carriers, LOHC).^2,3^ Although hydrogen can be produced in many ways, for example by using algae,^4,5^ gasification of biomass,^6,7^ photocatalytic and photoelectrochemical water splitting,^8^ or thermochemical cycles employing solar furnaces with metal/metal oxide cycles,^9,10^ the most promising method that matches the fluctuating production of renewable energies in a mega to gigawatt scale is still water electrolysis. For example, transport of biomass results in significant energy losses, and photocatalytic systems may work in single cells with direct sunlight but cannot be stacked.

Water electrolysis (WE) as an industrial-scale process dates back at least to the 1920s and is typically based on alkaline systems, in which cheap nickel-based electrodes are separated by a porous diaphragm, and >20 wt % KOH solutions are used as feed electrolyte.^11,12^ The drawback is the low productivity of these systems, which only operate within a narrow current range. Particularly when the current density decreases, hydrogen crossover is severe, and for safety reasons, hydrogen-in-oxygen levels below 2% need to be maintained to stay well below the explosion limit of approximately 4%.^13,14^ At higher current densities, the crossed-over hydrogen is diluted by the produced oxygen, and the systems can be operated safely. However, at very high current densities, the high cell voltage results in corrosion and thus shortens the lifetime of the electrolyzer system. The commercialization of chemically stable perfluorinated Nafion membranes, starting in the 1960s, enabled the development of proton exchange membrane (PEM) WE, which have a dense membrane and lower cell resistance. Hence, PEM WE operate at higher differential pressures and reach higher current densities than alkaline systems, which in turn reduces the foot print of electrolyzer systems.^15^

One shortcoming of PEM water electrolyzers is the highly corrosive, acidic environment, which requires platinum catalysts for the hydrogen evolution reaction and iridium-based catalysts for the oxygen evolution reaction. The global yearly supply of iridium is in the range of 5–7 tons,^16^ and it is mined as an accompanying element of platinum. Initially, the anodes of PEM WE used several mg of iridium per square centimeter. This needs to be reduced to 0.05 mg Ir/cm^2^ to allow the yearly global installation of about 5 GW PEM WE in the year 2040.^17^ In the scientific community, loadings as low as 0.036 mg Ir/cm^2^ have been reached, but the long-term stability of such systems needs to be validated and most probably improved further.^17^ A second shortcoming of PEM WE is that only perfluorinated membranes have shown sufficient stability to be used in commercial PEM WE. The persistence of perfluorinated compounds in the environment and human bodies raises expectations that governments will request to fade out these materials by new regulations.^18−20^

The ideal solution would be anion exchange membrane (AEM) WE, which uses a thin AEM as separator and are fed with pure water or low alkaline feed solutions (≤1 M KOH solution), so that a variety of non-noble catalysts is available. The dense membrane allows the use of differential pressure, and its small thickness results in a low resistance, which in turn allows to operate at higher current densities than traditional alkaline systems. The current bottleneck is the low alkaline stability of AEMs, which, however, starts to be overcome.^21^

**Figure 1:**
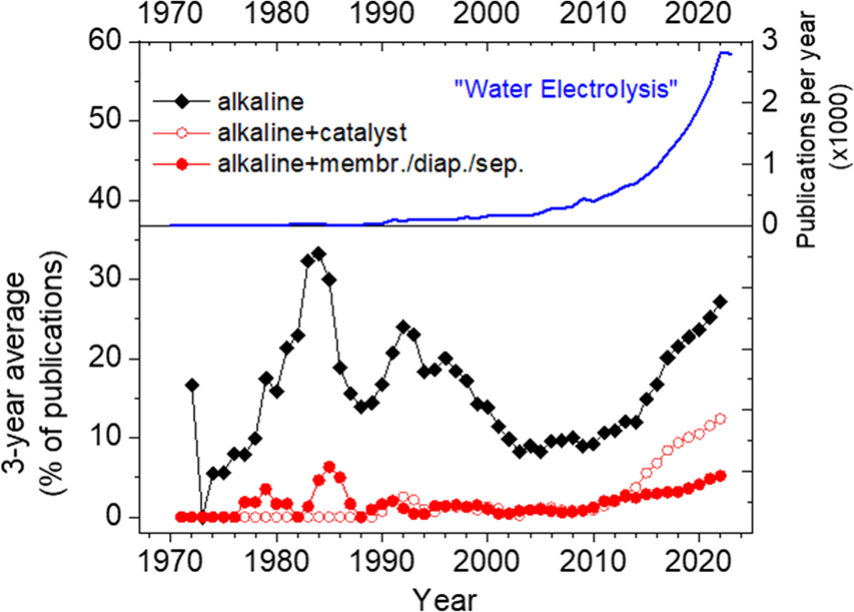
Literature on the topic “water electrolysis”, and percentage of the subgroups “alkaline”, “alkaline & catalyst”, and “alkaline & membrane or diaphragm or separator” (web of knowledge, 2024-01).

As Figure 1 shows, the number of publications related to water electrolysis increases exponentially, and reached over 2500 publications per year in 2021. Over the last 10 years, the percentage of publications dealing with alkaline systems steadily increased and reached a ratio of over 25%. Although the number of publications related to catalyst research for alkaline systems increases more strongly than that of separators, the electrode separator will eventually determine whether future systems will be more similar to current alkaline WE or AEM WE. So far, AEM WE research is focused on pure water and up to 1 M KOH feed solutions, because the AEM degradation accelerates with the increasing concentration of hydroxide ions. As an example, Enapter recommends operation of their AEM WE systems with 1% KOH (ca. 0.2 M KOH) feed solutions.^22^ The reason degradation accelerates with increasing KOH concentration is not only the concentration itself but also the smaller number of water molecules which is available to shield the hydroxide ions from the quaternary ammonium groups attached to the AEM.^23^ However, the performance of AEM WE improves when the KOH concentration increases. This is due to (a) the increasing conductivity when KOH is added to water, up to about 5–7 M,^24^ (b) a trade-off between high mechanical strength (low swelling) and efficient hydroxide transport in ionomer binder systems,^25^ and (c) the anodic oxidation of anion conducting polymers, which is a major reason for AEM WE degradation. The latter is slowed when KOH hinders direct contact between the polymer and the catalyst particles by participating in the formation of the double layer.^26^

Based on these facts, we expect that the increasing stability of AEM, and the improved performance when the feed solutions contain KOH, will shift the research direction away from pure water to KOH-containing feed solutions. In this light, it could even be that future WE systems will use KOH concentrations between the upper 1 M KOH limit for AEM WE followed by most researchers today, and the 5–7 M used in traditional alkaline systems.^27,28^ Furthermore, it was recently shown that an ion solvating membrane (ISM) based on sulfonated *p*-PBI (i.e., a quaternary-ammonium-free membrane) has a conductivity of >100 mS cm^–1^ in 1 M KOH solution.^29^ For these reasons, this current review will focus not only on porous diaphragms and ISM designed for traditional AWE, but will also discuss advances in the field of AEM. The literature discussed covers roughly the last 10 years of development, e.g., literature since 2011 for work on imidazolium-based ionenes, and work published since 2015 for AEMs prepared by polyhydroxyalkylation.

## 2 Alkaline Electrolysis with >20 wt % KOH Feed Solutions

Currently, industrial water electrolyzers operating with 20–30 wt % KOH (4.2–6.9 M) feed electrolytes use diaphragm membranes as separators, wherein the pores are filled with the electrolyte solution to facilitate the transport of ions and complete the electric circuit between the cathode and anode. At the same time, the electrolyte contains large amounts of H_2_ or O_2_, often even above the saturation level. These dissolved gases can permeate through pores driven by concentration gradients and the differential pressure between the cathode and anode, which influences product gas impurity and raises safety concerns. A number of factors influences the gas transport, like porosity, tortuosity, wettability, and pore size. The smaller the pore size of the separator, the lower the gas permeability, and hence the higher the gas purity, but the higher the ohmic resistance becomes. Therefore, comprehensive pore engineering is essential to maximize the ionic conductivity and minimize gas permeation through the separator. The wettability of diaphragms also affects the contact of the liquid electrolyte with the diaphragm surface, wherein a higher wettability enhances the liquid electrolyte flux, leading to reduced ohmic resistance. In the case of composite materials where hydrophilic ceramics are introduced, the higher the proportion of hydrophilic nanoparticles, the higher the wettability. However, concurrently this reduces the structural integrity of the separator, thereby increasing gas permeability. Other vital requirements include chemical stability in alkaline electrolyte (KOH) under operating conditions and scale fabrication since a long lifetime and larger cell area are critical for competitiveness in green hydrogen production.

Over the past 50 years, chrysotile (asbestos) has served well as a porous separator for industrial alkaline electrolyzers. Chrysotile, an important type of naturally occurring silicate minerals with the composition Mg_3_Si_2_O_5_(OH)_4_, is resistant to strong bases like KOH at low temperatures. At above 85 °C, the silica component dissolves^30^ and forms soluble potassium silicates (K_2_SiO_3_) and poorly soluble brucite, Mg(OH)_2_, that leads to diaphragm failure according to eq 1.
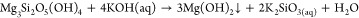
1

Chrysotile can easily be crumbled into bundles of fibrils with a length of millimeters to centimeters, which are mechanically strong and can be made into thin sheets. The fibrils have a diameter as small as 20–30 nm, forming very fine pores that strongly retain the electrolyte through capillary forces, and therefore withstand a certain gas pressure without losing the liquid. However, asbestos has been banned from commercial use in more than 60 countries due to the serious health hazards when the fibrils are airborne and inhaled. Another issue is to achieve constantly high gas purity when the electrolyzer is coupled with renewable energy sources.^31^

As an alternative to asbestos, polymeric membranes with/without ceramic fillers have been proposed. Separators solely based on high-temperature engineering thermoplastics like poly(phenylene sulfide) (PPS) felts have been fabricated, but these materials are generally hydrophobic, leading to low wettability of the pore system with the electrolyte solution. Therefore, other hydrophilic compounds such as oxide ceramics have been added to enhance the overall wettability. Such composite materials combine the stability of high-temperature engineering thermoplastics with the hydrophilic properties of ceramics. The most commonly known porous separator is AGFA’s Zirfon, which is a porous composite membrane of polysulfone (PSU) and ZrO_2_ particles, reinforced with PPS fibers.

### 2.1 Poly(Phenylene Sulfide) Felts

**Figure 2:**
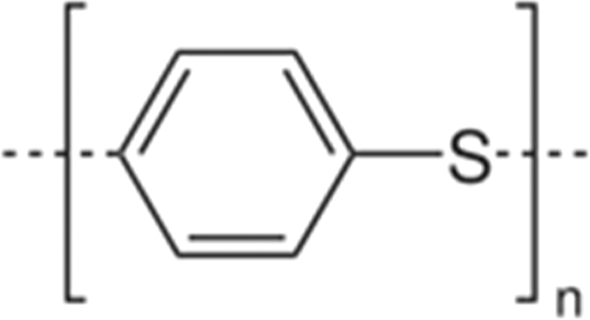
Structural formula of polyphenylene sulfide (PPS).^32^

Organic polymers, such as PPS, have been considered as promising separators for alkaline electrolyzers or as matrix materials for composite diaphragms and membranes. PPS is a thermoplastic polymer, composed of phenylene rings linked by thioether bonds in the 1,4-position (Figure 2).^32^ PPS is thermally, chemically and mechanically stable in a broad range of chemicals (acid and alkaline solutions) at prolonged periods and at elevated temperatures. It has a melting point of approximately 285 °C, withstands continuous use up to 190 °C and possesses inherent flame retardancy. It is commercially available as a linear or branched (cross-linked and cured) polymer.

PPS was discovered in 1888 as a byproduct of a chemical reaction and is nowadays industrially synthesized using either the Phillips method (synthesis of linear PPS resin, sodium sulfide method) or the sulfur method, in which dichlorobenzene and sulfur are polycondensed to produce the PPS resin at 175–250 °C under ambient pressure in polar solvents, such as hexamethylphosphoryltriamide or *N*-methylpyrrolidone.^33^

The three main forms of PPS membranes are flat, hollow fiber and ultrafine fiber membranes.^33^ Even though flat PPS membranes have an important research significance and practical application in many fields (oil–water separation, seawater desalination and dye wastewater treatment), shortcomings such as a low module loading density and insufficient mechanical properties affect the practical application of PPS flat membranes in electrolytic processes.

Development of PPS advanced membranes is challenged by the insolubility of PPS in most solvents and its high melting point. In general, PPS membranes should include the development and synthesis of PPS resin with high molecular weight and narrow molecular weight distribution, and reduction of the solid content of the casting solution to improve the porosity and through-pore content, while maintaining good mechanical properties.^33^

PPS fibers with a diameter below 5 μm, referred to as ultrafine fibers or microfibers, can be prepared using the melt-blowing method. The optimization of this process which could result in fabrication of a PPS melt-blown nonwoven felt for the use in alkaline electrolysis is ongoing.

PPS fabrics, composed of fibers processed from PPS (e.g., Ryton by Solvay or TORELINA by Toray), have been used in commercial alkaline electrolyzers as separators.^34^ The major manufacturers and suppliers of PPS fibers are Celanese, Evonik, and Toray. Fortron PPS (Celanese) can be extruded with melt-blown and spun-bonded technologies for melt-spun nonwovens.

PPS separators for alkaline water electrolysis cells are usually made of PPS fibers connected using either needling (nonwoven needle felt) or weaving (woven fabric).^33^ PPS needle felt separators possess excellent chemical, thermal, and mechanical stability in alkaline electrolytes.^35^ High porosity of PPS needle felt separators results in excellent ion conductivity thereof, but leads to a significant gas crossover. These separators can be used in alkaline water electrolysis under certain operational conditions^36^ or upon being subjected to material modifications to achieve the required gas tightness.

Undesired oxidation and cross-linking of PPS can be observed due to the low bond energy of the C–S–C bond. The oxidative properties of PPS can be adjusted by surface coating methods and direct addition of nanoparticles or antioxidants.^35^

Moreover, PPS is a hydrophobic polymer, which facilitates gas bubble accumulation at the diaphragm-electrolyte interface. Gas bubbles add resistance and thus reduce the electrolyzer’s efficiency. Therefore, it is important to improve the wettability of PPS fibers or felts.^37^ Methods to increase the hydrophilicity of PPS fibers are plasma treatment,^38,39^ ultraviolet light irradiation,^40^ or chemical oxidation. For example, PPS separators can be postmodified by a nitric acid treatment.^41^

Good wettability accompanied by the high temperature resistance of PPS results in an optimized performance of the separator in alkaline water electrolyzer cells.^33,42^

A 2000 h test of an AWE with PPS felt as a diaphragm was carried out by Hoadley at al. and showed that this separator could be a candidate to substitute asbestos diaphragms. However, an inadequate hydrophilic treatment of the PPS felt caused initial operational issues.^43^

Manabe et al.^44^ studied the performance of a composite diaphragm produced by Kawasaki Heavy Industries, Ltd. (KHI), composed of a nonwoven fabric of PPS and PSU. Significant fluctuations of the cell voltage in the zero-gap alkaline water electrolysis system with the KHI diaphragm indicate that the properties, such as hydrophilicity, and pretreatment of the diaphragm determine the performance of AWE.

Functionalization of the PPS fabric or felt by introducing polar groups into the polymer structure reduced the voltage drop.^45^ Incorporation of sulfonic, carboxylic or phosphonic acid groups can be undertaken on PPS polymer powder or flakes, as well as with PPS fabrics or felts.

The property of being resistant to almost all solvents up to 200 °C makes PPS a promising separator material. On the other hand, preparation of flat sheet PPS membranes via a solution phase inversion method remains a challenge due to a lack of suitable diluents suitable to dissolve PPS at low temperature while preserving its performance. Utilization of a single diluent system led to a monotonous morphology and insufficient mechanical stability of the PPS flat sheet membranes.^46^

Wang et al.^46^ prepared PPS flat sheet membranes from the ternary systems of PPS/dibutyl sebacate (DBS)/diphenyl ketone (DPK) by thermally induced phase separation (TIPS). Sandwich-like PPS flat sheet membranes composed of branch-like, bicontinuous, cellular structure were obtained with 16 wt % DBS content, resulting in the highest water flux and porosity among different DBS to DPK weight ratios. A larger amount of DBS, accompanied by higher polymer concentration and faster cooling rate, could improve mechanical properties, such as tensile strength and elongation at break, of these membranes. Godshall et al.^47^ used a thermally induced phase separation process for developing PPS gels with a benign solvent, 1,3-diphenylacetone (DPA). Gelation, which occurs at temperatures below 225 °C and is followed by freeze–drying solvent evacuation, results in formation of aerogels of low densities (0.11–0.25 g/cm^3^) and high porosities (82.2–92.3%), with the structure of elongated, interconnected fibrils that could be of interest for development of future PPS AWE separators.

Homogeneous materials for PPS-based separators usually cannot fulfill the stringent requirements for operation in AWE, such as good ion conductivity, low hydrogen and oxygen crossover, mechanical, chemical and thermal stability, and sufficient hydrophilicity. Therefore, similarly to application in other energy conversion fields,^48^ composite separators (diaphragms and membranes) modified with inorganic nano- and microscale particles, are also used in alkaline water electrolysis to achieve the required separator properties and enable efficient AWE systems. The main type of separators containing inorganic particles are nonwoven mats filled/impregnated with inorganic particles and composite membranes with particles incorporated in their structure or coated thereon.

For the fabrication, dry and wet impregnation, solution dipping and doctor blade coating are techniques used for the incorporation of inorganic particles into nonwoven, woven mats and membranes.^48^ Binders are used to prevent the agglomeration of the nano and micro particles and enable good adhesion to the fibers. The binder type and the particles/binder volume ratio need to be optimized to reach optimal separator performance.

A literature survey shows significant publication activity on the topics of PPS polymer membranes modified with inorganic particles in the field of battery research, but publications covering PPS separators with incorporated inorganic particles for the use in AWE are less numerous.

Ali et al.^49^ studied the effect of TiO_2_ nanoparticles size (18, 40, and 100 nm) on the AWE performance of composite separators comprising titania, polysulfone, and PPS supporting mesh. Smaller size of TiO_2_ nanoparticles led to increased bubble point pressure (BPP) and reduced H_2_ crossover, while larger particles decreased the ohmic resistance. Smaller particles exhibited significant interaction with PSU, and thus increased the resistance by reducing the pore size distribution. In this study, particles interacted with PSU rather than with the PPS support mesh.

Porous PPS diaphragms rapidly absorb KOH solution into their pores and therefore show high ionic conductivity. The shortcoming of highly porous PPS felts is the significant gas crossover especially when a differential pressure between the electrodes is applied. Therefore, the differential pressure should be minimized, and modification of the diaphragms’ structure with fillers to further reduce gas crossover is desired. Porous composite separators containing inorganic nano- and microscale particle fillers incorporated into PPS woven or nonwoven mats^50^ may simultaneously fulfill the contradicting requirements of high ionic conductivity and good gas separation of AWE separator materials, as well as enhancing wettability and stability in harsh alkaline conditions.

### 2.2 Zirfon Type Diaphragms

Currently, Zirfon Perl UTP series membranes are widely used separators for AEL. They employ zirconium dioxide (ZrO_2_) on a porous PSU basis, reinforced with PPS fibers, and are marketed under the name Zirfon (Agfa-Gevaert N.V.). Zirfon has a thickness of 500 ± 50 μm, a porosity of 55 ± 10% and a pore diameter in the range of 150 ± 50 nm.^51,52^ The zirconia particles help to wet the hydrophobic polymer, resulting in much faster electrolyte absorption than for pure PPS felts.

#### 2.2.1 Materials for Macroporous Diaphragms

##### 2.2.1.1 Organic Materials

PSU is renowned for its remarkable thermal stability and stability in highly alkaline environments.^53^ For AEM, aromatic ether bonds were identified as breaking points in contact with hydroxide ions. The reason that the aromatic ether bonds in PSU derivatives such as Udel or Radel do not react in contact with KOH solution is the high overall hydrophobicity of the polymer, which hinders access of hydroxide to the bulk material. Additionally, it can be easily processed via a phase inversion technique. This technique enables control over the microstructure and pore size distribution of the membrane, making it a popular choice for synthesizing diaphragms.^31,54^ Otero et al. employed sulfonated poly(ether ether ketone) (PEEK) as a binder which is a semicrystalline thermoplastic polymer. To enhance its hydrophilicity, PEEK was sulfonated, whereby −SO_3_H groups were incorporated into the polymer via an electrophilic substitution reaction with sulfuric acid.^55^ Perfluoroalkoxy alkanes (PFA) and polytetrafluoroethylene (PTFE) have recently emerged as organic materials for diaphragms.^56^ PFA and PTFE exhibit exceptional stability when in contact with KOH at high temperatures (>120 °C). However, they are not amenable to solution casting, which impedes their application to commercial process techniques for large-area membrane fabrication used in low-temperature diaphragm preparation, such as phase inversion. In addition, (per)fluorinated materials are likely to be banned or strongly restricted.

##### 2.2.1.2 Inorganic Nanoparticles

Incorporation of ceramic fillers has been shown to significantly improve the hydrophilicity of composite membranes and facilitate the formation of mesopores. The efficient wetting behavior of the separators plays a critical role in promoting the migration of OH^–^ ions through the separator. Various ceramic fillers have been explored for use in alkaline electrolyzers due to their ability to withstand extreme conditions of high alkalinity, voltage, temperature, and oxygen partial pressure. Among the commonly employed fillers are nickel oxide,^57^ CeO_2_,^58^ ZrO_2_,^58^ barite (BaSO_4_),^54^ zirconia toughened alumina (ZTA),^59^ yttria-stabilized zirconia (YSZ),^55^ TiO_2_,^60^ and Ni–Fe layered double hydroxide.^56^ There are some limitations when choosing proper ceramics in AEL. The use of electrically conductive material such as Ni is not feasible as they can lead to short-circuits. Inorganic materials such as Al_2_O_3_ and SiO_2_ offer high surface area and the flexibility of surface modification, but they are not resistant to alkaline conditions and may ultimately dissolve in concentrated electrolytes.

Chatzichristodoulou et al. reported purely ceramic separators based on yttria-stabilized zirconia (YSZ).^61^ A YSZ powder was dispersed in ethanol and 12 wt % polyvinylpyrrolidone (PVP) binder, which then was removed by sintering at 600 °C in air. The porous ceramic separator had pores with a mean diameter of 70 nm and a porosity of 45%, and was used in an alkaline electrolysis cell operating at 200–250 °C and 20–40 bar. While the performance was very promising (1.5 ± 0.1 V for 400 h at 500 mA cm^–2^), the mechanical properties are probably equally challenging as for the brittle ceramics used in solid oxide cells.

#### 2.2.2 Diaphragm Fabrication

The production of composite diaphragms usually involves the phase inversion method, which is a process of transitioning a polymer from a liquid (dissolved) state to a solid state. One commonly used type of diaphragm in alkaline electrolyzers is the Zirfon separator, which consists of a PPS fabric that is symmetrically coated with a slurry of PSU and zirconium dioxide (ZrO_2_). To produce the Zirfon separator, PSU is dissolved in *N*-methyl-2-pyrrolidone (NMP) and polymeric additives such as poly(vinylpyrrolidone) (PVP) or poly(ethylene glycol) (PEG) may be added to increase the resulting membrane’s porosity.^62^ ZrO_2_ nanopowder is then added to the mixture, and the resulting slurry is homogenized before being cast onto a substrate, such as a glass plate, using a doctor blade. The sample is then immersed in a nonsolvent, such as distilled water or isopropanol, to extract the NMP solvent. The morphology of the resulting membranes can be varied widely depending on the composition and viscosity of the casting solution as well as on the composition of the nonsolvent bath and its temperature.^52,63,64^

Recently, fluoropolymers, such as PFA and PTFE, with high porosity have been used as separator templates. However, even though their chemical resistance is outstanding, there are no solvents that can dissolve them at room temperature. This impedes the application of the processing technology obtained from the well-developed low-temperature diaphragm preparation using phase inversion. The pore-filling strategy was proposed to synthesize PTFE-based composite membranes. First, the pristine PTFE membrane is immersed in an ethanol solution to improve its hydrophilicity. Then, the treated PTFE membrane is immersed in a mixed solution of precursors, and the precursor precipitates in the pores of the PTFE membrane’s skeleton. The resulting composite membrane is obtained after heat treatment.^56^

##### 2.2.2.1 Polymer–Inorganic Material Interaction

**Figure 3:**
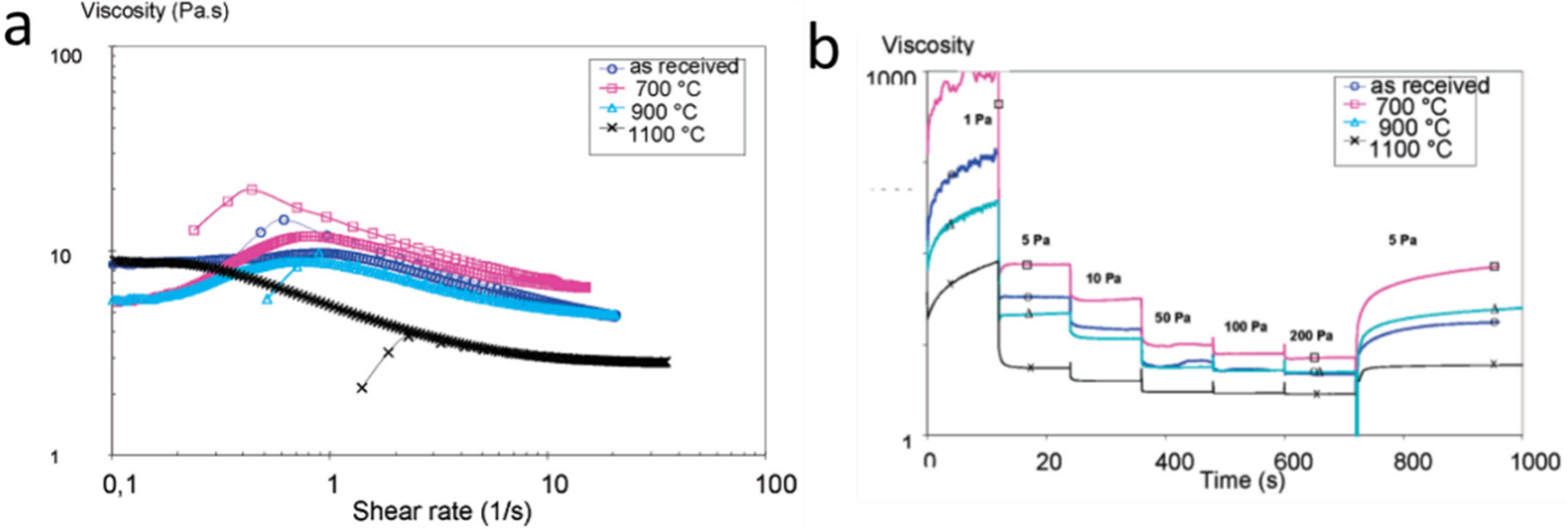
(A) Viscosity of the casting suspension (18 wt % PSU/NMP + 10 vol % ZrO_2_) as a function of the shear rate (stress-ramp experiment) and the sintering temperature of the zirconia particles. (B) Time dependent rheological behavior of 80 wt % ZrO_2_+20 wt % PSU casting suspensions with sintered zirconia. Reproduced with permission from reference (65). Copyright 2006 American Chemical Society.

Conventional composite membranes typically consist of a polymer with a small addition of inorganic nanoparticles. However, the Zirfon-type separator for AEL contains more than 80 wt % of inorganic nanoparticles to maximize its wettability in liquid electrolyte. Many researchers experienced nanoparticle loss from composite separators during phase inversion, which can lead to a dismantled polymer matrix because the slurry is not carefully prepared. Therefore, the interaction between a hydrophobic polymer and hydrophilic ceramics must be investigated as it significantly affects the properties of composite membranes. Aertz et al.^65^ studied the interaction between PSU and ZrO_2_ particles as a function of particle sintering temperature to understand the role of ZrO_2_ on the formation, morphology, and properties of composite membranes. They controlled the surface properties of the particles by means of surface area and surface hydroxyl group functionalities by varying the sintering temperatures between 500, 700, 1000, and 1100 °C. They found that changes in surface characteristics of the particles determined the amount of PSU that was adsorbed. As the sintering temperature increased, the amount of adsorbed polymer decreased, resulting in a less viscous and weaker suspension. The interaction was inferred from the amount of PSU adsorbed at the surface of ZrO_2_ measured by high-performance liquid chromatography and thermogravimetric analysis (see Figure 3). The authors concluded that the different interactions between sintered ZrO_2_ particles and the PSU solution influenced the rheological properties of the casting solution and the strength of the formed network, which affects the membrane formation process and resulting structures.

#### 2.2.3 Cell Performance

**Figure 4:**
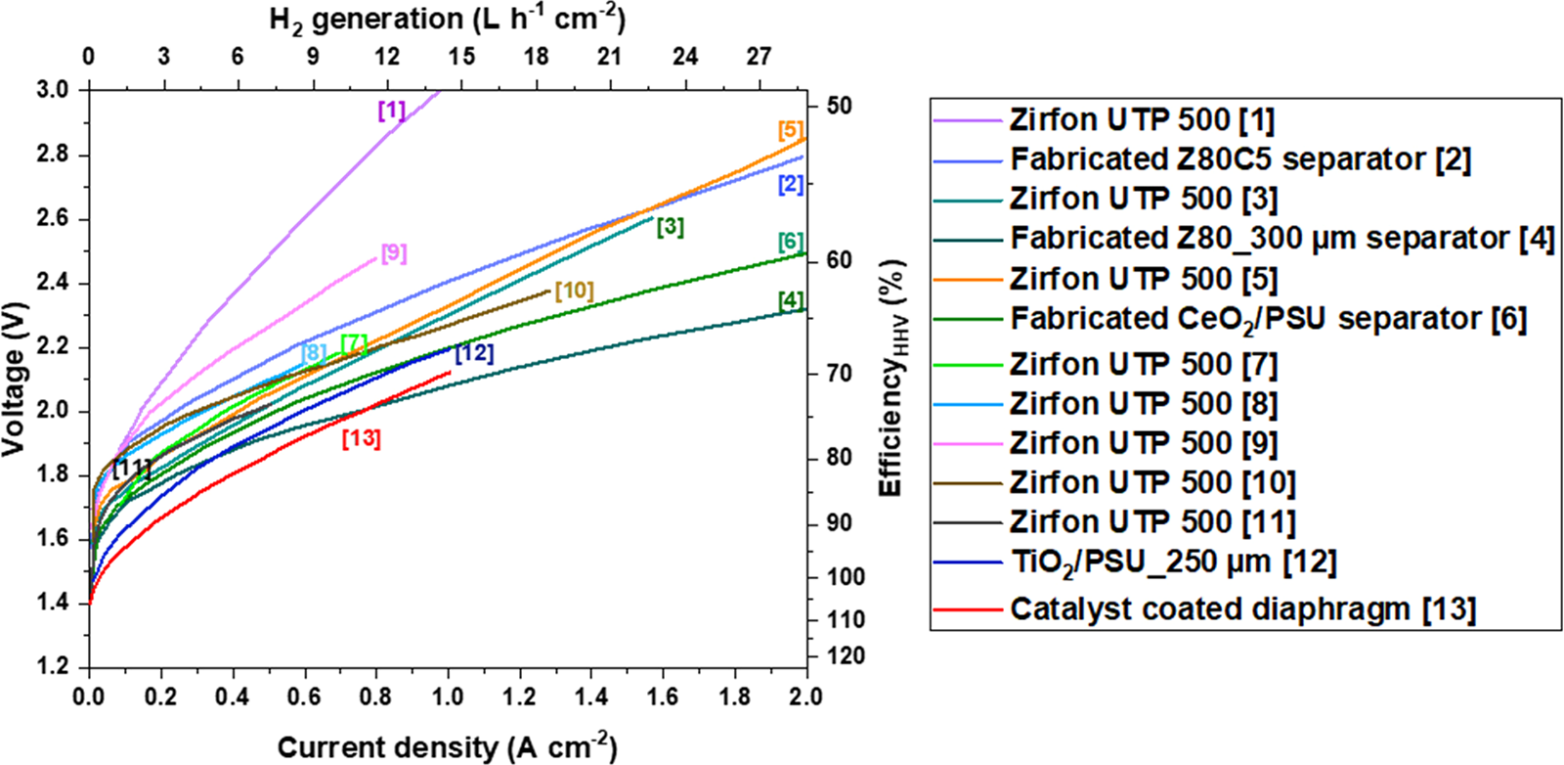
Published performances of AWE cells using diaphragms and uncoated electrodes. Details and references are listed in Table 1.

**Figure 5:**
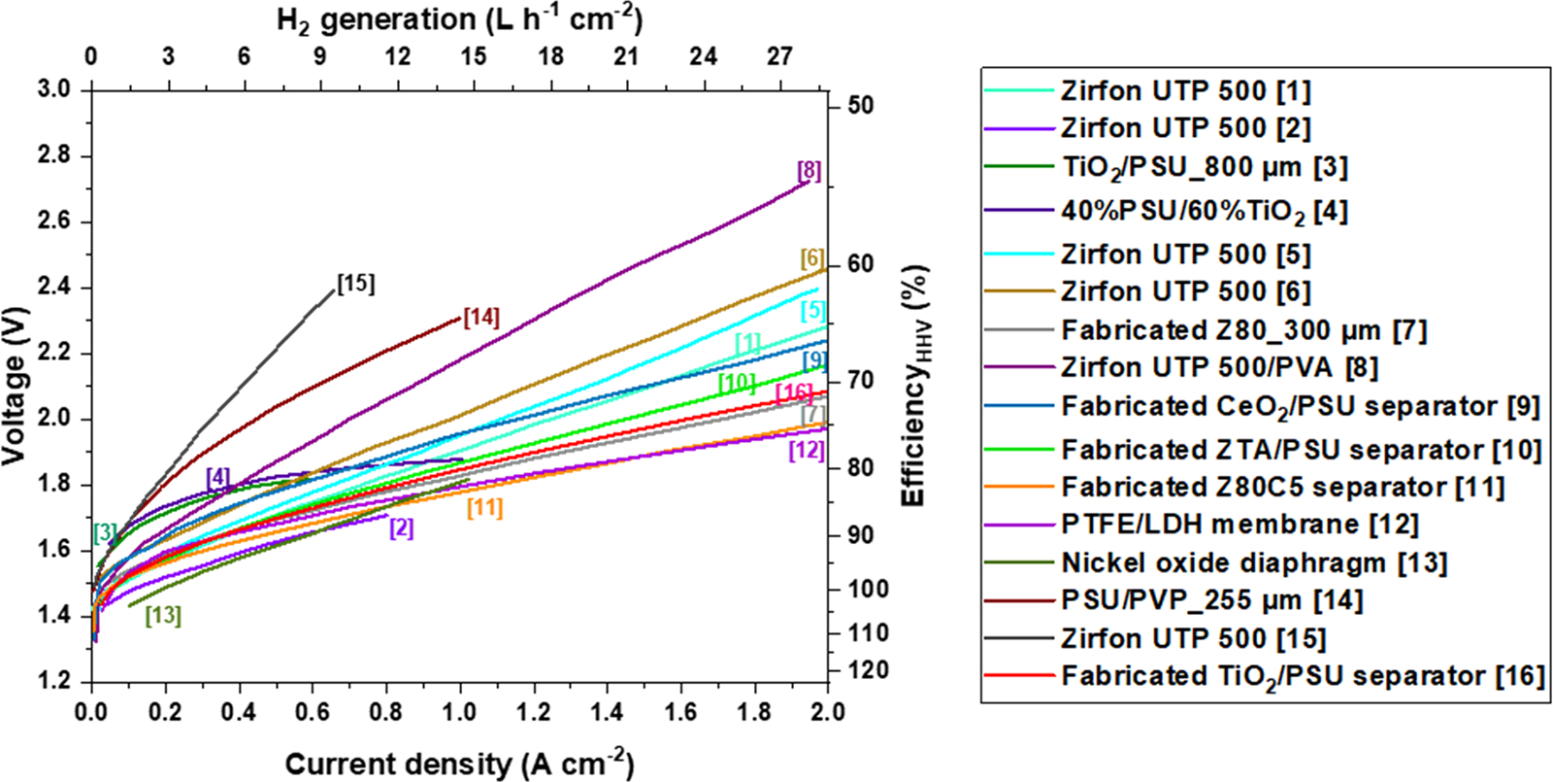
Published performances of AWE cells using diaphragm and coated Ni-electrodes. Details and references are listed in Table 2.

The separator performance in a cell can be assessed based on the voltage–current characteristic measured, which is primarily influenced by the interplay between electrodes and separators. The kinetic overvoltages at the electrodes dominate the overpotentials at low current densities, while those at higher current densities are mainly dictated by the ohmic resistance of the separator membrane. The differences in slopes observed in the higher current density region are primarily attributed to the ohmic resistance of the selected membrane, disregarding any mass transport resistance. Hence, a lower slope in the high current density area indicates better diaphragm performance. To differentiate the effects of overvoltage arising from electrodes and separators, two sets of IV performance curves are presented in this study. One shows IV performance curves using uncoated Ni-electrodes (Figure 4), and the other shows coated Ni-electrodes (Figure 5).

Figure 4 compares voltage–current characteristics of AWE cells equipped with diaphragms and uncoated Ni-electrodes (such as Raney Ni, Ni foam, Ni powder, etc.). The detailed conditions are summarized in Table 1. The cells equipped with uncoated Ni-electrodes exhibit the average performance of 2.3 ± 0.1 V at 1 A cm^–2^, corresponding to 64% efficiency (based on higher heating value, HHV). The slopes in the low current density region (0.1–0.2 A cm^–2^) are high due to the use of uncoated Ni-electrodes. The exceptionally high slope of IV curve #1 is due to the use of low concentration (10 wt %) KOH electrolyte.

**Table 1:** Details of the Cells with Uncoated Electrodes Which Are Compared in Figure 4

assigned number (in Figure 4)	separator	cathode	anode	temperature (°C)	KOH electrolyte (wt %)	reference
[1]	Zirfon UTP 500 (∼500 μm)	Ni foam	Ni foam	80	10	J.W. Lee et al. (2022)^66^
[2]	Fabricated 5 wt % cellulose blended 80 wt % ZrO_2_/15 wt % PSU separator Z80C5 (468 ± 30 μm)	Ni foam	Ni foam	80	10	J.W. Lee et al. (2022)^66^
[3]	Zirfon UTP 500 (∼500 μm)	Ni foam	Ni foam	80	30	H.I. Lee et al. (2020)^67^
[4]	Fabricated 80 wt % ZrO_2_/20 wt % PSU_Z80_300 μm	Ni foam	Ni foam	80	30	H.I. Lee et al. (2020)^67^
[5]	Zirfon UTP 500 (∼500 μm)	Ni foam	Ni foam	80	30	J.W. Lee et al. (2020)^58^
[6]	Fabricated 85 wt %CeO_2_/15 wt % PSU composite separator (460 ± 25 μm)	Ni foam	Ni foam	80	30	J.W. Lee et al. (2020)^58^
[7]	Zirfon UTP 500 (∼500 μm)	Ni foam	Ni foam	60	30	H.I. Lee et al. (2020)^68^
[8]	Zirfon UTP 500 (∼500 μm)	Ni foam	Ni foam annealed for 24 h at 600 °C	80	32.5	C. Karacan et al. (2022)^69^
[9]	Zirfon UTP 500 (∼500 μm)	Ni foam	Ni foam	80	30	A. Alam et al. (2020)^70^
[10]	Zirfon UTP 500 (∼500 μm)	Ni foam	Ni-perforated plate	80	24	M.R. Kraglund et al. (2019)^71^
[11]	Zirfon UTP 500 (∼500 μm)	Ni plates	Ni plates	80	30	W.B. Ju et al. (2018)^72^
[12]	5 wt % PSU/75 wt % NMP/5 wt % PVP/15 wt % TiO_2_ diaphragm (∼250 μm)	STS plates	STS plates	80	30	S.S. Kumar, et al. (2018)^73^
[13]	Catalyst-coated diaphragm (CCD) (∼500 μm)	Raney Ni powder	Preheated Ni foam	80	32.5	C. Karacan et al. (2022)^74^

It is noted that IV curve #13 shows a significant reduction in overvoltage in the low current density region despite the use of Raney Ni powder. Karacan et al.^74^ directly coated a commercial Raney nickel onto the cathode sides of Zirfon UTP 500 diaphragms in a catalyst coated membrane (CCM) approach via blade coating. The overvoltage dropped by ∼200 mV at 0.2 A cm^–2^ compared to the conventional zero-gap configuration, in which Ni foams are mechanically pressed on each side of the separator. This result suggested that the catalytic activity can be enhanced by employing the CCM concept for zero-gap alkaline water electrolysis.

It is observed that the slopes in the higher current density region are almost identical when the same separator (i.e., Zirfon UTP or a homemade separator) are used because ohmic resistance starts to dominate the reaction rate.

It is remarkable that the slopes of cells with the fabricated separators made by research group of Prof. Cho W.C. (see the IV curve #4, #6, and #12) are much lower than those with Zirfon PERL separator. The performance gap originates from the ohmic resistance difference between commercial Zirfon and other separators, which will be discussed in the later section. Besides, the difference between IV curves #4 and #6 is attributed to the thickness of the separator. The cell with a thinner separator (less than 300 μm, line #4) demonstrates a higher performance because the separator thickness is directly related to the overpotential.

Figure 5 summarizes IV curves of AWE cells equipped with a diaphragm and coated Ni-electrodes, so-called advanced electrodes. We excluded PGM catalysts in this study since it overestimates the AWE cell performance and is not conducive at cost competitiveness. Detailed conditions are summarized in Table 2.

**Table 2:** Details of Electrolytic Unit Cells with Coated Electrodes, as Shown in Figure 5 (Non-PGM Catalysts)^a^

assigned number (in Figure 5)	separator	cathode	anode	temperature (° C)	KOH electrolyte (wt %)	reference
[1]	Zirfon UTP 500 (∼500 μm)	VPS NiAl/Mo coated perforated plate	VPS NiAl coated perforated plate	80	30	M. Schalenbach et al. (2016)^75^
[2]	Zirfon UTP 500 (∼500 μm)	VPS NiAl/Mo coating	VPS NiAl/Co_3_O_4_ coating	80	29	P. Vermeiren et al. (1998)^76^
[3]	20 wt % PSU/5–15wt % PVP/200 wt % (in % of polymer weight) TiO_2_ diaphragm (∼800 μm)	Ni–Mo-coated expanded Nickel grids	NiCo_2_O_4-_coated expanded Nickel grids	80	28	N.V. Kuleshov et al. (2019)^77^
[4]	40 mass. % PSU/60 wt % TiO_2_	Porous coatings on Ni mesh modified by NiPx catalyst	Porous coatings on Ni mesh modified by by NiCo_2_O_4_ catalyst	90	39	N.V. Kuleshov et al. (2020)^78^
[5]	Zirfon UTP 500 (∼500 μm)	Raney-type-NiMo	Raney-type-Ni	80	24	M.R. Kraglund et al. (2019)^71^
[6]	Zirfon UTP 500 (∼500 μm)	Raney Ni	LDH-NiFe	80	30	H.I. Lee et al. (2020)^67^
[7]	Fabricated 80 wt % ZrO_2_/20 wt % PSU_Z80_300 μm	Raney Ni	LDH-NiFe	80	30	H.I. Lee et al. (2020)^67^
[8]	Zirfon UTP 500 coated with PVA (∼500 + 6 μm)	Raney Ni	LDH-NiFe	80	30	S. Kim et al. (2022)^79^
[9]	Fabricated 85 wt % CeO_2_/15 wt % PSU composite separator (460 ± 25 μm)	Raney Ni	LDH-NiFe	80	30	J.W. Lee et al. (2020)^58^
[10]	fabricated 85 wt % ZTA/15 wt % PSU composite separator (430 ± 5 μm)	Raney Ni	LDH-NiFe	80	30	M.F. Ali et al. (2022)^59^
[11]	Fabricated 5 wt % cellulose blended 80 wt % ZrO_2_/15 wt % PSU separator Z80C5 (468 ± 30 μm)	Raney Ni	LDH-NiFe	80	30	J.W. Lee et al. (2022)^66^
[12]	PTFE/LDH composite membrane with PGM-free catalyst (20 ± 1 μm)	CoP	FeNi LDH	60	28	L. Wan et al. (2021)^56^
[13]	nickel oxide diaphragm (400–500 μm)	Raney Ni-zinc	Raney Ni-zinc	100	40	J. Divisek et al. (1982)^80^
[14]	25 wt % PSU/75 wt % PVP blend membrane (255 μm)	NiMo	FeNi LDH	60	20	D. Aili et al. (2020)^81^
[15]	Zirfon UTP 500 (∼500 μm)	NiMo	FeNi LDH	60	20	D. Aili et al. (2020)^81^
[16]	Fabricated 85 wt % TiO_2_/15 wt % PSU composite separator (430 ± 5 μm)	Raney Ni	LDH-NiFe	80	30	M.F. Ali et al. (2023)^49^

a VPS stands for vacuum plasma spraying.

In general, cells equipped with advanced electrodes demonstrates high performances of maximal 1.9 ± 0.1 V at 1 A cm^–2^, corresponding to 77% efficiency (HHV basis). However, the IV curves #8 and #14 exhibits a lower performance despite the use of advanced electrodes. The cells of IV curve #8 and #14 used a PVA cross-linked Zirfon separator and a PSU/PVP blend, respectively. Hydrophilic and dense poly(vinyl alcohol) (PVA) and PVP were incorporated into the porous separators in order to enhance the hydrophilic nature of PSU. However, the introduction of the hydrophilic polymer to PSU did not increase in the conductivity, but rather acted as additional resistance.

The polarization curves of #2 and #13 shows the highest performances of 1.7 ± 0.1 V at 0.7 A cm^–2^, which is comparable to state-of-the-art PEM electrolyzers. The data were already reported in the 1980s and the performances are not reproduced elsewhere. Even though key materials and experimental conditions of both systems are similar, the polarization curve #2 in 1998 exhibited a reduced potential by ∼200 mV compared to that of #1 in 2016.

The polarization curves #11 and #12 exhibit the highest efficiency of 75% (HHV) while achieving the highest current density of 2 A cm^–2^ at <2 V. The separator for #11 was made by adding hydrophilic cellulose nanocrystals (CNCs) into a Zirfon-type separator, while the separator for #12 was a 201 μm thick PTFE/LDH composite membrane, which adopted a CCM configuration.

For the cells reported by researchers from Seoultech (#7, #9, #10, #11), which all shows the remarkable performance of 2 A cm^–2^ at around 2.0 V, the performance mainly results from homogeneous distribution of nanoparticles, addition of hydrophilic materials, and thinner fabrication of the separator/membrane. For comparison, commercial PEM electrolyzers from Siemens, Hydrogenics, and Proton Onsite (NEL) are reported to operate at the current density 2 A cm^–2^ at 2.2 V.^82^ These results suggest that AWE with PGM-free catalysts can compete with PEM electrolysis in terms of polarization performance by adopting advanced separators.

The cell performance can vary depending on the diaphragm thickness, electrolyte concentration, and temperature. However, discrepancies between IV curves are observed even though similar materials are used in the same or similar operation condition. De Groot et al.^83^ explained that the cell performance can be influenced by the zero gap configuration, uneven current distribution, a finite gap, and the effect of bubbles. This limits the comparability of cell performances measured in different groups through the steady state polarization curve, and it is recommendable to evaluate the cell performance combining both durability test and polarization curves.

The cell #14 with a polymeric PSU/PVP diaphragm was reported to operate at a low current density (500 mA cm^–2^ at 2.0 V) for the relatively short operating time of ∼170 h because the PSU/PVP cell showed gradual degradation. The cell with PTFE/LDH composite membrane shows the best performance in Figure 5 (line #12). It was fabricated into a catalyst coated membrane (CCM) to reduce the contact resistance while maximizing catalytic activity. However, a durability test was conducted at the mild condition of 500 mA cm^–2^@1.65 V and 60 °C for 180 h. The cell with the PVA coated Zirfon separator (#8) also operated at mild conditions of ∼350 mA cm^–2^ at 1.8 V for 180 h. The CCM cell sustained ∼650 mA cm^–2^ at 2 V for 1,000 h, which coincides with the performance from IV curve #13. However, the CCM cell experienced detachment of catalyst particles which were washed out from both the anode and cathode outlet streams, suggesting that the further study on the catalyst/membrane interface is needed for stable continuous operation. The cells with zero-gap configuration operated stably under test conditions for a certain period of time at a low degradation rate, supporting that AWE is a simple and established technology.^84−86^

**Figure 6:**
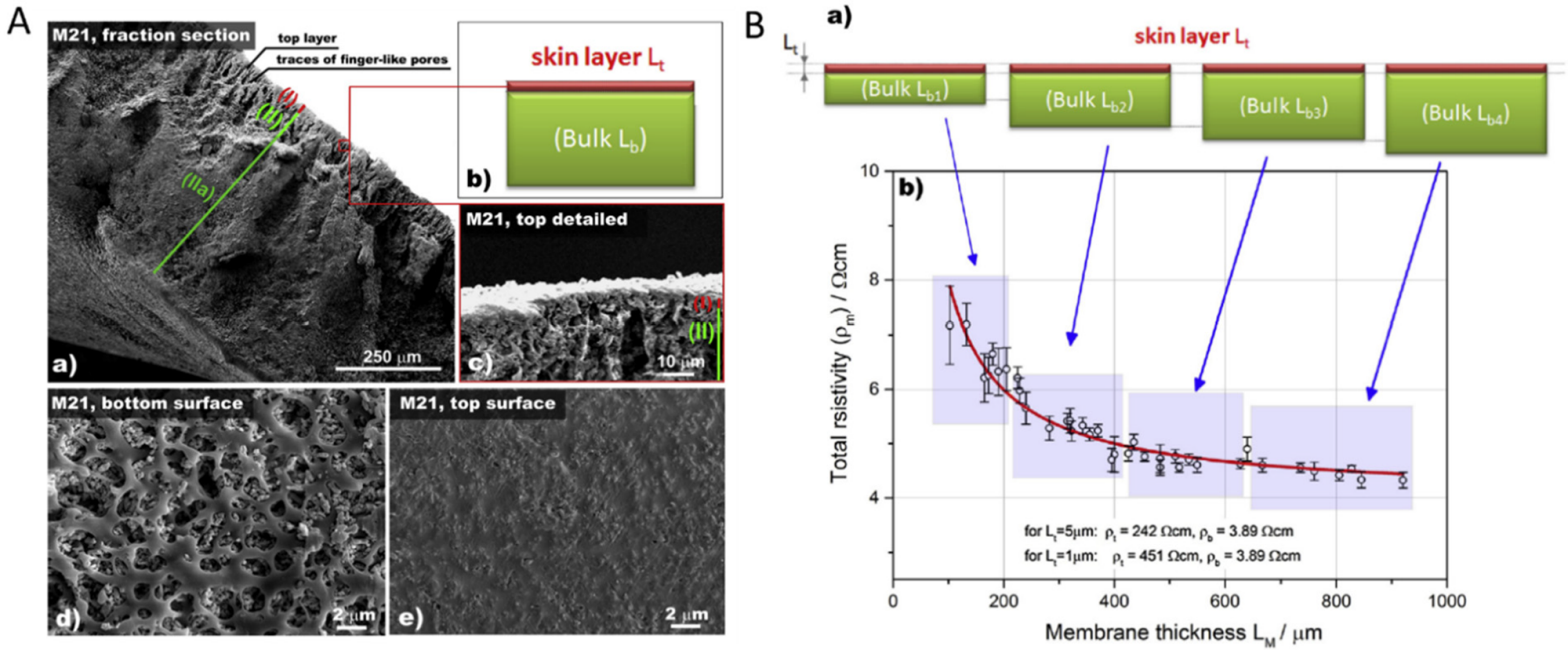
(A) (a) Cross sectional overview on membrane M21 indicating two distinct regions of the membrane, top layer and bulk; (b) schematic representation of membrane cross-section with distinct two microstructures (c) detailed cross sectional view on top layer; (d) bottom surface of the membrane M21; (e) top surface of membrane M21. (B) Variation of the total resistivity as a function of membrane thickness. (a) Schematic representation of top layer (Lt) and bulk microstructure (Lb) ratios for membranes of various thicknesses. (b) Influence of membrane thickness on total resistivity (M21). Reproduced with permission from reference (54). Copyright 2015, Elsevier.

It is typical for composite separators to have asymmetric structures, consisting of a thin top layer and a bulk part.^87^ The top layer, with a thickness of 1–5 μm, is characterized by low porosity and high polymer concentration, while the bulk part, which represents the majority of the membrane volume, features significantly higher porosity. The formation of the dense and polymer-rich top layer is attributed to its direct exposure to the nonsolvent during the coagulation stage. High porosity of the bulk part, coagulated at the end of the process, is demonstrated by the presence of large pores in the μm-range (Figure 6(A)). The researchers found that total resistivity strongly depends on the resistivity of the outer skin layer, indicating that optimizing the top layer conductivity is crucial for improving the resistivity of membranes prepared through the phase inversion process.

**Figure 7:**
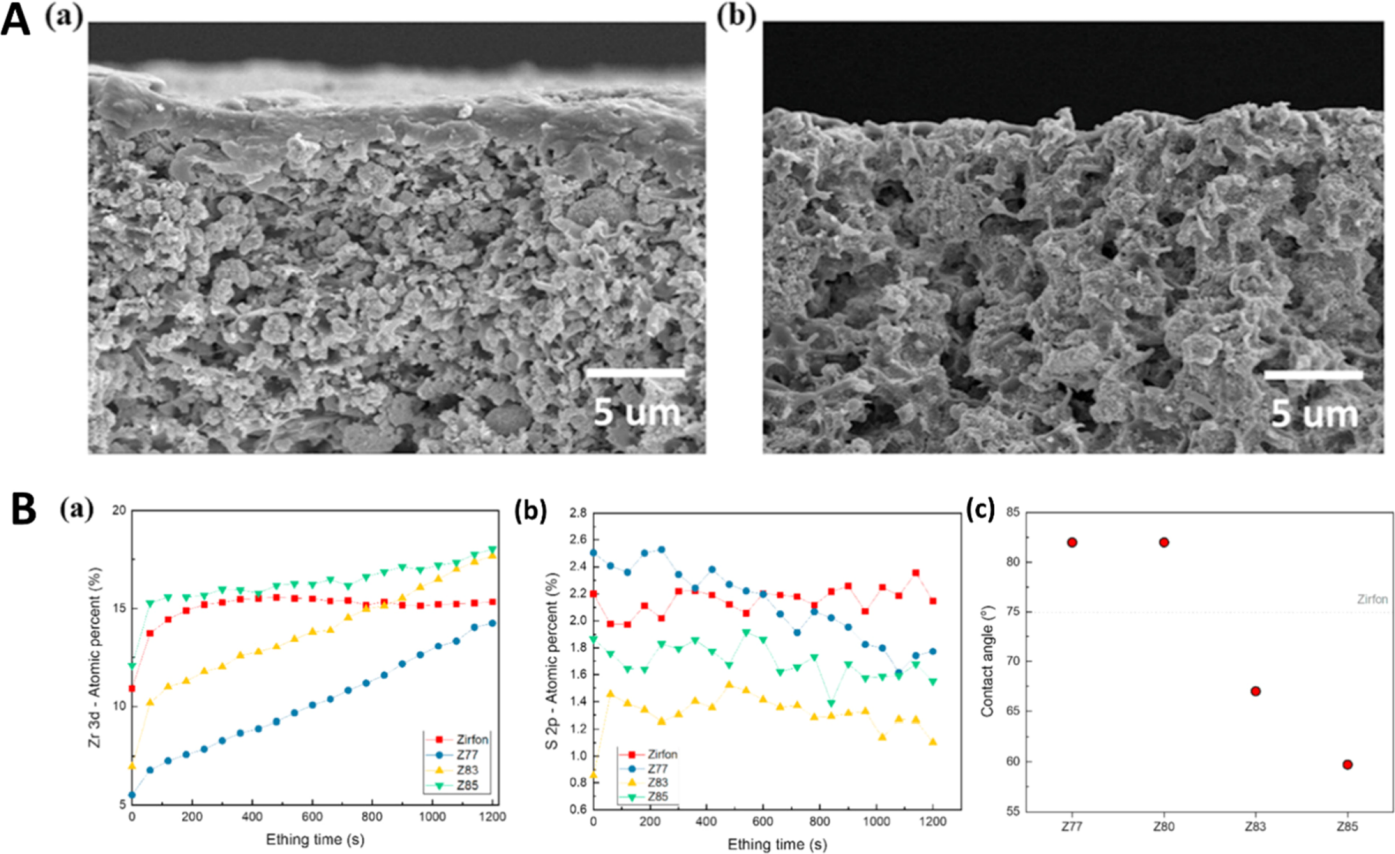
(A) Cross-sectional SEM images of (a) Zirfon PERL and (b) fabricated separator Z85 from Seoultech. (B) Amount of (a) Zr 3d and (b) S 2p atomic percentage of the prepared separators from Seoultech and Zirfon PERL separator as a function of the etching time from 0 to 1220 s through XPS depth profile. (c) Contact angle of the prepared separators measured at room temperature in KOH 30 wt % solution. Reproduced with permission from reference (67). Copyright 2020, Elsevier.

This assumption is supported by comparing cross-sectional SEM images of separator membranes. Skin layers with a thickness of 1–5 μm were clearly observed on the top of Zirfon UTP. In contrast, it was difficult to distinguish a skin layer on a separator made by researchers at Seoultech, which had a homogeneously porous overall structure (Figure 7(A)).

XPS analysis at various argon etching times was conducted to investigate element concentrations (Figure 7(B)). It shows that the zirconium concentration of the Z85 separator without observable skin layer is higher than that of the Zirfon PERL separator, while the sulfur concentration of Z85 is lower than the value obtained for Zirfon PERL, which is consistent with the results presented in Figure 7(A). The contact angle between the 30 wt % KOH electrolyte and the separator surface showed that its magnitude decreases as the zirconia content increases. The contact angles of Z83 and Z85 (less than 67°) are smaller than that of the Zirfon PERL separator (75°), which contains a polymer-rich layer (Figure 7(B)). Hence, the polymer-rich layer of Zirfon PERL apparently contributes to its high contact angle and ohmic resistance.

### 2.3 Ion Solvating Membranes

The pore diameter of Zirfon separators, as an example for diaphragms, is around 150 nm in the bulk, but smaller close to the surface.^51^ Most AEM have hydrophilic domains in the range of 1 nm or larger, which develop by phase separation of the hydrophobic polymer backbone and the hydrophilic quaternary ammonium groups when water is absorbed. Ion solvating membranes (ISM) are membranes which have a low tendency for phase separation, and absorb KOH solution into the volume between the polymer chains by swelling. This is achieved by locating the hydrophilic groups directly on the polymer backbone, and providing a high density of charged or polar groups. The most investigated aqueous alkaline system is KOH doped polybenzimidazole (PBI), which was first described by Xing and Savadogo for application in alkaline fuel cells.^88^ While pristine PBI itself is practically nonconductive, strong bases deprotonate the benzimidazole amine groups (p*K*_a_ = 12.8) under formation of the corresponding benzimidazolides, which increases the polarity of the polymer and results in increased absorption of water and KOH.^89^ This is especially attractive for use in electrolyzers, where the membranes are in contact with KOH feed solution, and thus are not expected to lose the absorbed KOH. Ion-solvating membranes based on *m*-PBI are described in more detail in section 2.3.1, followed by a discussion about *m*-PBI derivatives and non-PBI based ion-solvating membrane based chemistries in section 2.3.2.

#### 2.3.1 PBI-Based Ion Solvating Membranes

**Figure 8:**
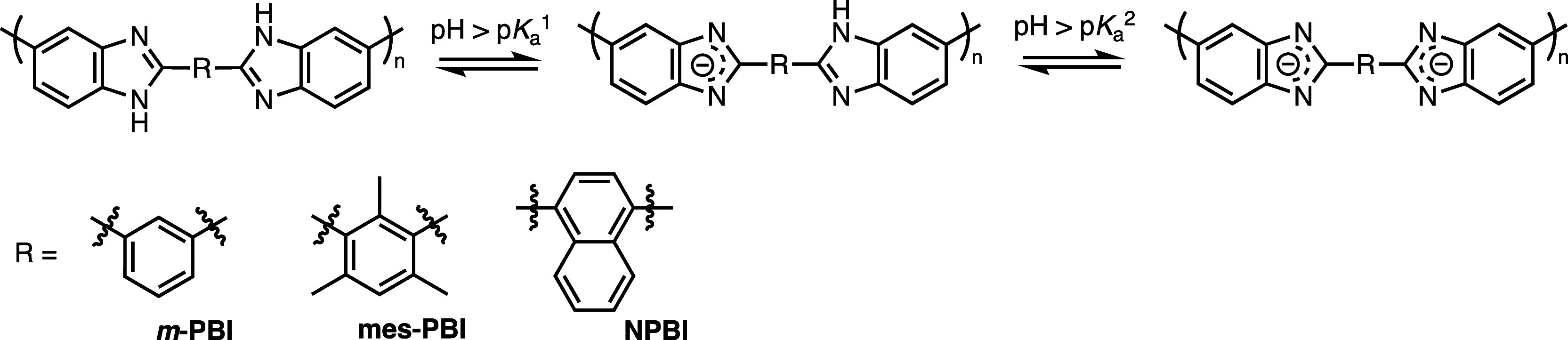
Acid–base equilibrium of *m*-PBI and structure analogues thereof in alkaline environment.

Polybenzimidazole refers to a large family of polymers with benzimidazole units as part of the repeat unit. A general description of the synthesis, properties, and structural scope of polybenzimidazoles reported in the scientific literature can be found elsewhere.^89^ The most explored PBI derivative for alkaline electrolysis is poly(2,2′-(*m*-phenylene)-5,5′-bibenzimidazole) (*m*-PBI), which is obtained by condensation–polymerization of isophthalic acid and 3,3′-diaminobenzidine. When exposed to an aqueous alkaline environment, the neutral polymer is in equilibrium with the deprotonated form since the benzimidazole units are weakly acidic, as shown in Figure 8.

The electrolyte uptake and the effective KOH concentration of the aqueous phase within the membrane matrix depends on the KOH concentration of the surrounding solution, which in turn determines the position of the equilibrium.^90,91^ For *m*-PBI membranes prepared by solution casting from organic solvent, the electrolyte uptake at room temperature increases from around 23% in 5 wt % KOH to 134% in 25 wt % KOH.^92^ Further increasing the KOH concentration of the surrounding solution beyond 25 wt % results in decreasing electrolyte uptake of the membrane due to dehydration. The ion conductivity peak seems to coincide with the electrolyte uptake peak, typically reaching 100 mS/cm or even higher in 20–25 wt % depending on temperature and specific conditions.^93^ When combined with highly active nickel-based electrode chemistries, *m*-PBI membranes can support current densities reaching 2000 mA/cm^2^ at <1.85 V.^71^ Furthermore, it shows good gas barrier characteristics and low H_2_ crossover.^94^

Although some work indicated that physical aging is the reason for membrane degradation,^95^ there is a common believe that the main challenge with *m*-PBI membranes under alkaline water electrolysis conditions is the polymer backbone degradation, which results in gradually decreasing molecular weight and eventually membrane failure due to loss of low molecular weight fractions and thus thinning of the membrane, and because chain scission decreases the degree of entanglement. Under simulated operating conditions, the stability window of *m*-PBI membranes in 25 wt % KOH at temperatures close to 90 °C has been found to be around 4 months, after which the membrane spontaneously disintegrated when handled.^96^ By cross-linking the membrane matrix, the stability window could be extended to around 6 months. At device level, the degradation seems to occur faster than under simulated operating conditions. Due to additional stressors such as contacting with hydrogen/oxygen and rough electrode surfaces, electrolyte flows, pressure differentials, polymer oxidation near the anode, etc. the maximum reported lifetime at device level is a couple of weeks at 80 °C in 24 wt % KOH.^71^ With an alternative processing method that allows for higher electrolyte uptakes, combined with mechanical reinforcement, the lifetime could be increased to >1000 h.^97^ Using platinum group metal-free catalysts, a current of 1.76 A cm^–2^ was achieved at 1.8 V. It is noteworthy that the increased electrolyte uptake of these membranes shifted the usable KOH concentration range in the feed solution from ≈15 wt % to <10 wt % KOH (2 M). Presumably, the reduced alkalinity will slow down degradation and thus increase the lifetime.

Recent computational studies indicate that the degradation proceeds by hydroxide ion nucleophilic attack at the fraction of neutral (nondeprotonated) benzimidazole moieties (see Figure 8),^28^ which seems supported by model system studies.^98^ Within the structural scope of this work, the trend was that derivatives with electron-rich arylene linkages showed significantly higher stability than structures with electron-withdrawing substituents, which could potentially guide the design and synthesis of new PBI chemistries with improved stability.

#### 2.3.2 ISM beyond *m*-PBI

As discussed in section 2.3.1, alkaline ion-solvating membranes based on *m*-PBI combine high conductivity with good gas barrier characteristics. However, the polymer degradation under alkaline conditions limits the lifetime at the device level, which has stimulated research and development of alternative membrane chemistries. The introduction of methyl substituents in the 2, 4, and 6 positions of the *m*-phenylene linkages is an effective strategy to improve the stability of anion exchange membranes based on fully or partially *N*-alkylated polybenzimidazoliums,^99^ as it hinders nucleophilic attack at the labile benzimidazolium C2 position (this will be discussed in detail in section 3.3). This concept has also been investigated for the neutral form of the polymer (mes-PBI, see Figure 8), and was indeed found to suppress degradation of the corresponding PBI derivative. However, for this derivative the stability improvement was most evident in aqueous KOH of moderate concentrations (5–10 wt %).^100^ Other alternative PBI chemistries include structures linked by bulky naphthalene groups (NPBI, Figure 8)^28^ as well as sulfonated versions thereof.^101^ In a very recent work, Dayan et al. showed that sulfonated *p*-PBI absorbs so large amounts of electrolyte that it needs to be cross-linked to control the swelling. These membranes survived 6 months in 1 M KOH at 80 °C without loss of mechanical properties, showed a conductivity of >100 mS cm^–1^ at room temperature in 1 M KOH, and showed a very stable performance in an AEMWE over the tested 500 h. This work shows that the problem of low alkaline stability of AEMs could simply be solved by substituting AEMs with ISMs.^29^

As indicated by the computational and model system studies discussed in section 2.3.1,^98,102^ the degradation of PBI mainly proceeds via the fraction of neutral benzimidazole units. A rational strategy in the design of new PBI chemistries for alkaline ion-solvating membranes could therefore focus on steric hindrance approaches that effectively protect these linkages.

**Figure 9:**
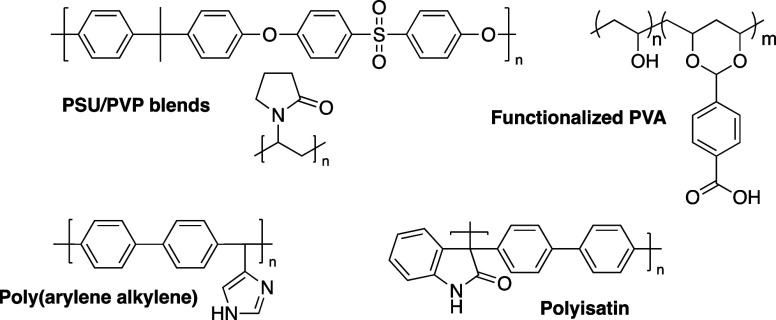
Summary of non-PBI polymer systems that have been explored for alkaline ion-solvating membranes.

To mitigate the intrinsic stability limitations of PBI based membranes, chemistries based on alternative hydrophilic polymer systems have been explored as summarized in Figure 9. PVP, for example, has no protonic group but is highly hydrophilic and thus absorbs water or electrolyte. Presumably, also some portion of the amide groups are hydrolyzed to amine and carboxylic acid, which should intensify the interaction with KOH solutions. Reported systems include homogeneous PSU/PVP blends,^81^ poly(vinyl alcohol-*co*-vinyl acetal)s,^103^ and imidazole functionalized poly(arylene alkylene)s.^27^ A notable example is polyisatin, which combines high electrolyte uptake and conductivity with exceptional stability in alkaline environment.^104^ Polyisatin has also been investigated as a component in homogeneous blends with *m*-PBI, which was found to increase the stability of the membranes compared with pristine *m*-PBI by molecular reinforcement.^105^ The synthesis of poly(arylene alkylenes) and polyisatines are typically carried out by super acid mediated polyhydroxyalkylation, which will be discussed in detail in section 3.4.

### 2.4 Cation Exchange Membranes

Perfluorosulfonic acid is one the few known polymer families that combine excellent chemical stability in alkaline environment with high hydrophilicity, and is used as electrode compartment separator and cation conductor in chloroalkali cells for the production of chlorine and sodium hydroxide from brine.^106^ In aqueous KOH it shows a high K^+^-selectivity, with a K^+^ transference number of around 99%.^107^ With only 1% conductivity contribution from the OH^–^ ion, it is therefore not an appropriate electrolyte system for alkaline water electrolyzers, because the limiting molar conductivity of OH^–^ ions is 2.7 times higher than that of K^+^ ions.^108^ The low hydroxide ion conductivity of perfluorosulfonic acid membranes in aqueous KOH is further supported by the high ohmic resistance measured by Yeo et al. for alkaline water electrolyzers equipped with Nafion membranes.^109^ In that work, Nafion membranes were employed in concentrated alkaline (up to 30% NaOH and KOH) electrolytes at elevated temperatures (up to 160 °C). The rationale of the idea was to take advantages of the excellent stability of the CEMs in the neutralized salt form, which exhibit a glass transition temperature of as high as up to 200 °C as well as enhanced mechanical strength.

To enhance the swelling and hence the anion conductivity of the CEM in alkaline media, perfluorosulfonate membranes with expanded morphologies were prepared with water contents as high as λ = 85 [H_2_O]/[−SO_3_K] and an ionic conductivity of 0.2 S cm^–1^ after equilibration in aqueous KOH.^110^ This was achieved by blending with a secondary hydrophilic component, to reduce the concentration of ionic sulfonates responsible for the effective Donnan exclusion. In this way, the transference number of OH^–^ was increased, which significantly lowered the ohmic resistance at the device level.^110^ While potassium exchanged Nafion 211 showed only an in-plane conductivity of 7.8 mS cm^–1^ in water and a through-plane conductivity of 2.3 mS cm^–1^ in 1 M KOH, a potassium exchanged sulfonated PSU reached up to 22.9 mS cm^–1^ in water and up to 12.0 mS cm^–1^ in 1 M KOH.^111^ These values are still too low to be useful for alkaline water electrolyzers, but suggest that strongly swollen cation exchange membranes, reinforced with a strong porous support, may allow to reach a useful conductivity range in highly concentrated KOH solutions.

## 3 AEM for Use in Alkaline Solutions < 2 M

**Figure 10:**
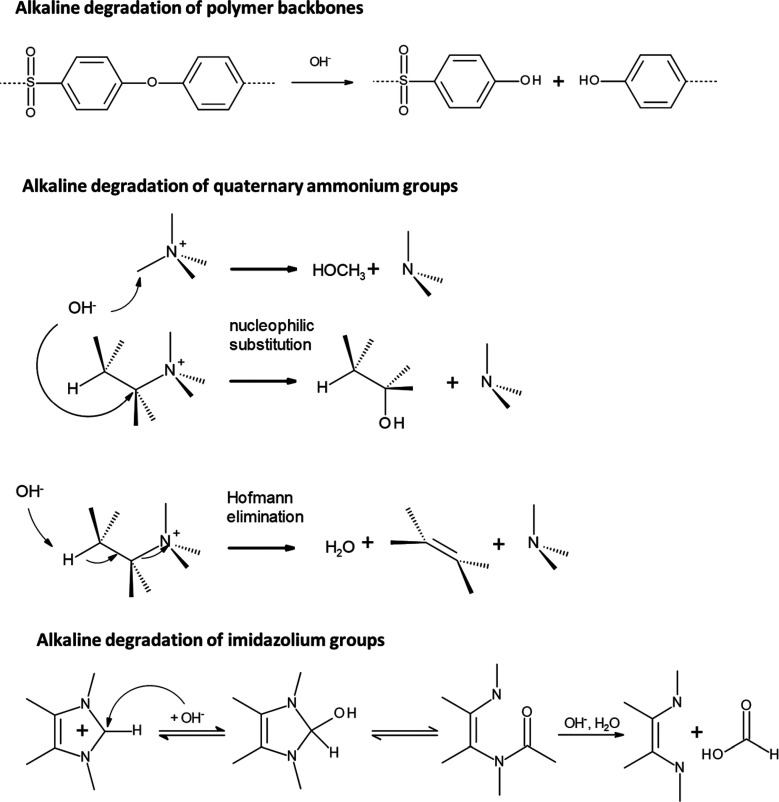
Degradation of AEM in alkaline conditions, reproduced with permission from reference (21). Copyright 2021, American Society of Mechanical Engineers.

AEMs contain cationic functional groups. Although a plethora of different chemistries has been investigated, most cationic groups are based on quaternary ammonium, which suffers from poor alkaline stability. Degradation proceeds via nucleophilic substitution or, if the structure has beta-hydrogen atoms, via Hofmann elimination. Imidazolium ions degrade by reaction of hydroxide ions with the carbon atom in position 2 (Figure 10). For this reason, most early work on AEM focused on neutral or low alkaline applications, like electrodialysis or water purification. Research on hydroxide exchanged AEM accelerated over a decade ago, as an effort to develop alkaline anion exchange membrane fuel cells, (AAEMFCs) which potentially could be operated with noble-metal free electrodes.^112^ This was vital, because some early electric vehicle fuel cell stacks used up to 60 g platinum, a lot compared to the 180 tons mined in 2021.^113^ Work on AEM for AAEMFC revealed that arylene ether bonds can hydrolyze under alkaline conditions, and commonly used polymer backbones like PSU, PEEK or polyphenylene oxide (PPO) would not be good materials to start fabrication of AEMs.^114^ However, it has to be stressed that the pristine materials are practically stable in the bulk, because their hydrophobicity prevents contact between hydroxide ions and the sensitive arylene ether groups.

In 2011 and 2012, the first papers on AEM-based water electrolysis appeared.^115−117^ Failure of the nuclear reactor in Fukushima spurred the transition to renewable energy, and green hydrogen production by water electrolysis with AEM moved into the focus.^118^ Simultaneously, stable polymer backbone chemistries and functional groups were developed,^21^ and first alkaline membrane water electrolyzers are now commercialized (for example, by Enapter). Until now, AEMs are haunted by the ghost of the past—low alkaline stability—and reports on using AEM in traditional alkaline water electrolyzers (i.e., >20 wt % KOH) are rare. However, first reports of AEMs which show a half-life of several thousand hours in 10 M KOH at 100 °C appeared,^119^ and it could well be that the research focus shifts in the near future to higher KOH concentrations, to find an optimum between low cell resistance, long lifetime and gas purity.

### 3.1 Effect of KOH Concentration on AEMs

An increasing KOH concentration has three main effects on AEMs: (1) the uptake of KOH increases, (2) water uptake decreases, and (3) the alkaline stability decreases. Indirectly, this affects conductivity, presumably mechanical properties and lifetime.

In principle, AEMs should not absorb KOH, because Donnan exclusion hinders cations from entering the membrane. However, the barrier for anions, the Donnan potential, is strongest in pure water and at high internal ion concentrations, and decreases when the ionic strength of the solution outside of the membrane increases. In a simplified explanation, mobile anions prefer to stay in the vicinity of the immobile cationic groups, and rather inside of the charged membrane than in the solution. Any co-ion (i.e., cation) which approaches the membrane/solution interface experiences electrostatic repulsion, preventing it from entering the membrane. When the ionic strength of the solution increases, more counterions are also found on the outer side of the interface. Already at concentrations of around 1 M KOH, this effect is so strong that AEMs absorb significant amounts of KOH, and at the very high ionic strength of traditional alkaline electrolyzer feed solutions (e.g., 20–30 wt % KOH), membranes will lose ion selectivity and act as a diaphragm with transference numbers approaching those of pure aqueous solutions.^120^

The water uptake of AEMs is connected to the osmotic pressure difference between the membrane and the solution. In pure water, the ionic strength in the hydrophilic domains of the membrane is obviously larger than in the external solution, and water rushes into the membrane to reduce this concentration difference. When the ionic strength of the solution increases, the membrane absorbs less water.

**Figure 11:**
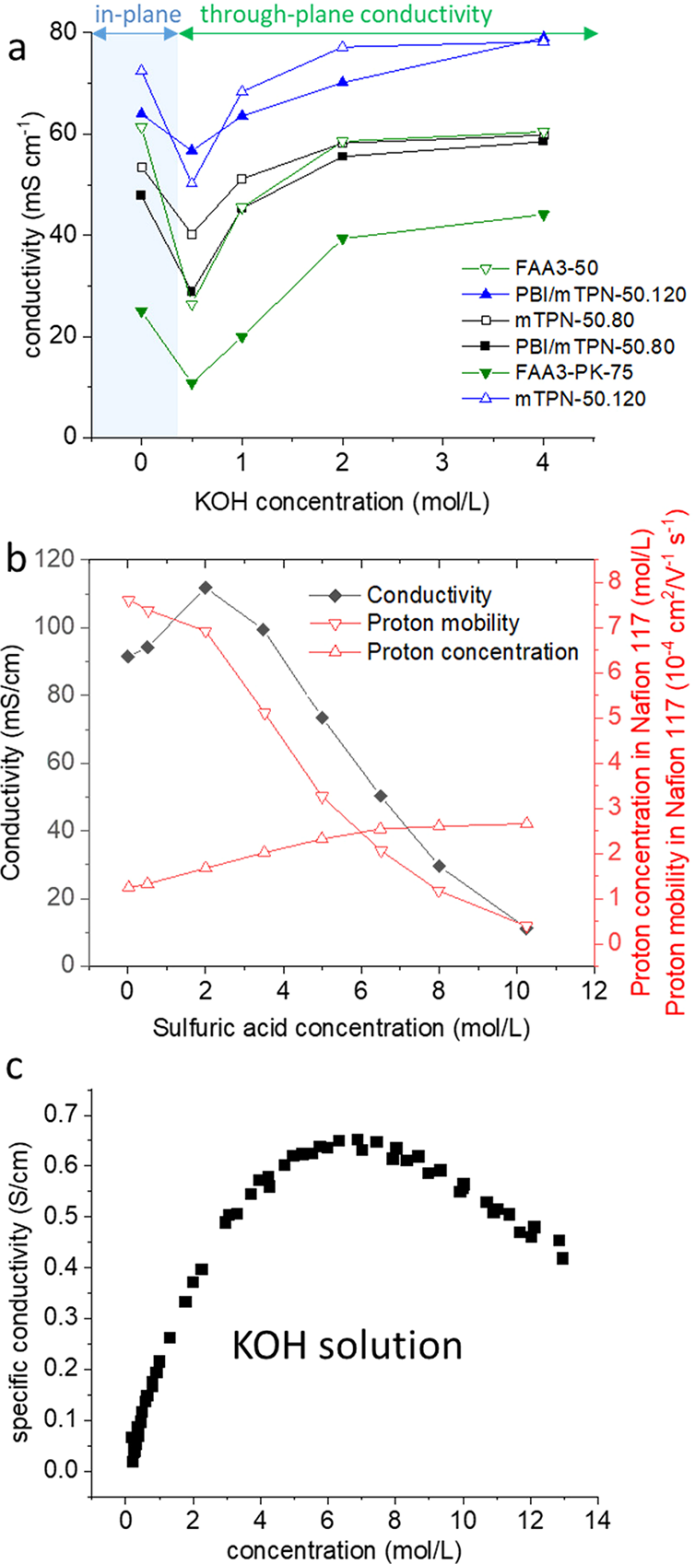
(a) Dependence of conductivity on KOH concentration, reproduced from.^121^ (b) Conductivity, proton mobility, and proton concentration of Nafion 117 in sulfuric acid,^122^ (c) Conductivity of KOH solutions; (a) reproduced with permission from reference (121). Copyright 2021, Elsevier.

The decreasing water content should result in increasing tensile strength and Young’s modulus, and in decreasing conductivity. On the other hand, the absorption of KOH increases the conductivity and may decrease the tensile strength. For example, 6 different AEMs showed an initial drop in the conductivity, when the solution was changed from water to 0.5 M KOH (it should be cautioned that this could also be related to the different measurement methods, in-plane conductivity in water, and through-plane conductivity in KOH solutions), and then an increasing trend between 0.5 and 4 M KOH.^121^ In this range, the effect of the absorbed KOH on the conductivity is stronger than that of the reduced water uptake (Figure 11a). For Nafion 117, Tang et al. reported a maximum conductivity in ca. 2 M sulfuric acid, and a constantly decreasing conductivity in the range of 2 to 10 M (Figure 11b).^122^ The reason for this is that conductivity is proportional to the product of proton concentration and mobility. While the first increases when sulfuric acid is added to the external solution and then levels off at higher concentrations, the latter constantly decreases in the same range. A possible explanation is that the contribution of Grotthuss mechanism to the total conductivity decreases when the water uptake decreases.

Because an increasing KOH concentration in the external solution reduces the water uptake of the membrane and allows absorption of additional KOH, hydroxide ions in the membrane are less well solvated, which increases their basicity and nucleophilicity, and the alkaline stability of the membrane decreases.^23,123^ For this reason, electrolyzers with an AEM are usually not operated with the highly alkaline feed solution used in traditional alkaline water electrolyzers, i.e., > 25 wt % KOH (ca. 5.5 M, around the maximum conductivity of KOH solution, Figure 11c). The use of pure water would be ideal from an industry perspective, because it may allow to directly use PEM water electrolysis technology (pumps, tubings, cell geometries etc.). However, such cells require an ionomer in the electrode to establish a pathway for the hydroxide ions between membrane and catalyst particles, which raises other engineering challenges. One is the stability of phenyl groups in contact with platinum catalysts; apparently, phenyl groups can be oxidized to phenols, which are slightly acidic and thus consume an equimolar amount of the mobile hydroxide ions.^25,124,125^ Another challenge is the trade-off between high water uptake to reach low resistance and mechanical strength of the binder; at high water uptakes, catalyst particles are easily washed out, visibly as a discoloration of the feed solution.^25^ Therefore, many AEM electrolyzers operate with 1 M KOH feed solution, the commercialized stacks from Enapter operate at 1% (ca. 0.2 M KOH) KOH feed.^22^Figure 11 suggests that a higher KOH concentration of about 2 M KOH could result in optimized balance between membrane resistance and alkaline degradation for many AEMs.

Although the degradation of AEMs under alkaline conditions is quite well understood, some details remain less investigated. For example, degradation of benzyltrimethylammonium-functionalized polymers preferentially occurs by hydroxide attack on the benzyl position, and to a lesser extent on the methyl groups.^126−128^ Bauer et al. reported that degradation occurs to 65% by attack on the benzyl group and 35% by attack on the methyl group.^126^ The test condition was an accelerated test at 160 °C in 2 M KOH in glycol. Khalid et al. stored a membrane in 1 M KOH at 60 °C for 1 month, and XPS analysis showed the absence of nitrogen signals, indicating a higher selectivity than 65/35 (benzyl/methyl).^128^ The knowledge gap is the effect of temperature and hydroxide concentration on the benzyl/methyl selectivity. For other quaternary ammonium groups, also competition between S_N_2 and Hofmann elimination will play a role, and the parameters affecting the selectivity for Hofmann elimination, S_N_2 (benzyl) and S_N_2 (methyl) are not well investigated, and could be the reason different groups report different degradation rates.^127^ When AEM are used in DI water or with very low concentrated KOH feed solutions, degradation of the functional group will result in high membrane resistance.^128^ However, if the concentration of the electrolyzer feed solution is high (20–30 wt % KOH, ca. 4–7 M), Donnan exclusion is low and the membrane absorbs excess KOH. Hypothetically, if the conditions can be controlled to favor degradation pathways toward amine-functionalized polymers (e.g., attack of OH on the methyl groups of benzyl-TMA → polymer-NR_2_ + MeOH), phase separation into hydrophilic and hydrophobic domains may remain, especially for polymers below the glass transition temperature. In such a case, the hydrophilic domains would be filled with KOH solution, and the membranes would function as ion solvating membranes. In that case, controlled degradation of AEMs in highly alkaline solutions could result in separators for alkaline water electrolyzers which do not degrade completely, or which even do not degrade—if contribution to total conductivity from absorbed KOH is larger than that of hydroxide counterions.

### 3.2 Recent Advances in AEM Development

The main challenges in AEM development have been the low alkaline stability of the functional groups and the polymer backbone. Some guidelines helped in the development of alkaline-stable AEM.

The alkaline stability of model compounds can be investigated by dissolving them in hot alkaline solution and monitoring their degradation by an appropriate method like NMR spectroscopy.^129^ A systematic investigation of several model compounds indicated that pyridinium, guanidinium, and simple imidazolium ions that are not sterically protected have such a low alkaline stability, that their half-life in 6 M NaOH and 160 °C is too short to be measured.^130^ The often used benzyltrimethylammonium motif showed a half-life of 4.18 h, while the heterocyclic compounds showed the longest lifetime, for example 13.5 h for monomethylated (1,4-diazabicyclo[2.2.2]octane) (DABCO), 87.3 h for fully methylated piperidine, and 110 h for 6-azonia-spiro[5.5]undecane (ASU), two piperidine rings connected by a shared nitrogen atom. At first glance, this is an unexpected result, because these structures have beta-hydrogen atoms, which can react in Hofmann eliminations. The explanation is that Hofmann elimination requires an antiperiplanar conformation of the ammonium group and the beta-proton, which is less easy to obtain for heterocyles than for open-chain analogues (e.g., ethylammonium). Another not directly expected finding was that trimethylammonium (TMA) ions show increasing stability in the order ethyl-TMA < benzyl-TMA < hexyl-TMA < methyl-TMA. Methyl-TMA is most stable, because Hofmann elimination cannot occur, and hexyl is more stable than ethyl, because the required antiperiplanar orientation is less easily obtained, because the long chain hinders the necessary rotation. Another consideration is the electron density and the fact that electron-donating groups inductively stabilize ammonium ions.

Stable polymer backbones were developed by removing ether groups. Suitable example polymer structures are polystyrene,^131,132^ styrene-ethylene-butylene-styrene (SEBS) block copolymers,^133−135^ polynorbornene,^136−138^ polyphenylene,^139^ and polybenzimidazole.^97^

The combination of stable functional groups and stable polymer backbones led to the commercialization of several new AEMs, like Versogen’s piperidinium functionalized poly(arylene alkylene)s, Orion polymer’s TM1 (a poly(arylene alkylene) with TMA attached to the polymer backbone through a pentyl chain), and Dioxide Materials’ polystyrene-based Sustainion membranes, which seem to be surprisingly stable considering that they use an imidazolium functional group.^21^

### 3.3 Polyimidazolium Membranes

**Figure 12:**
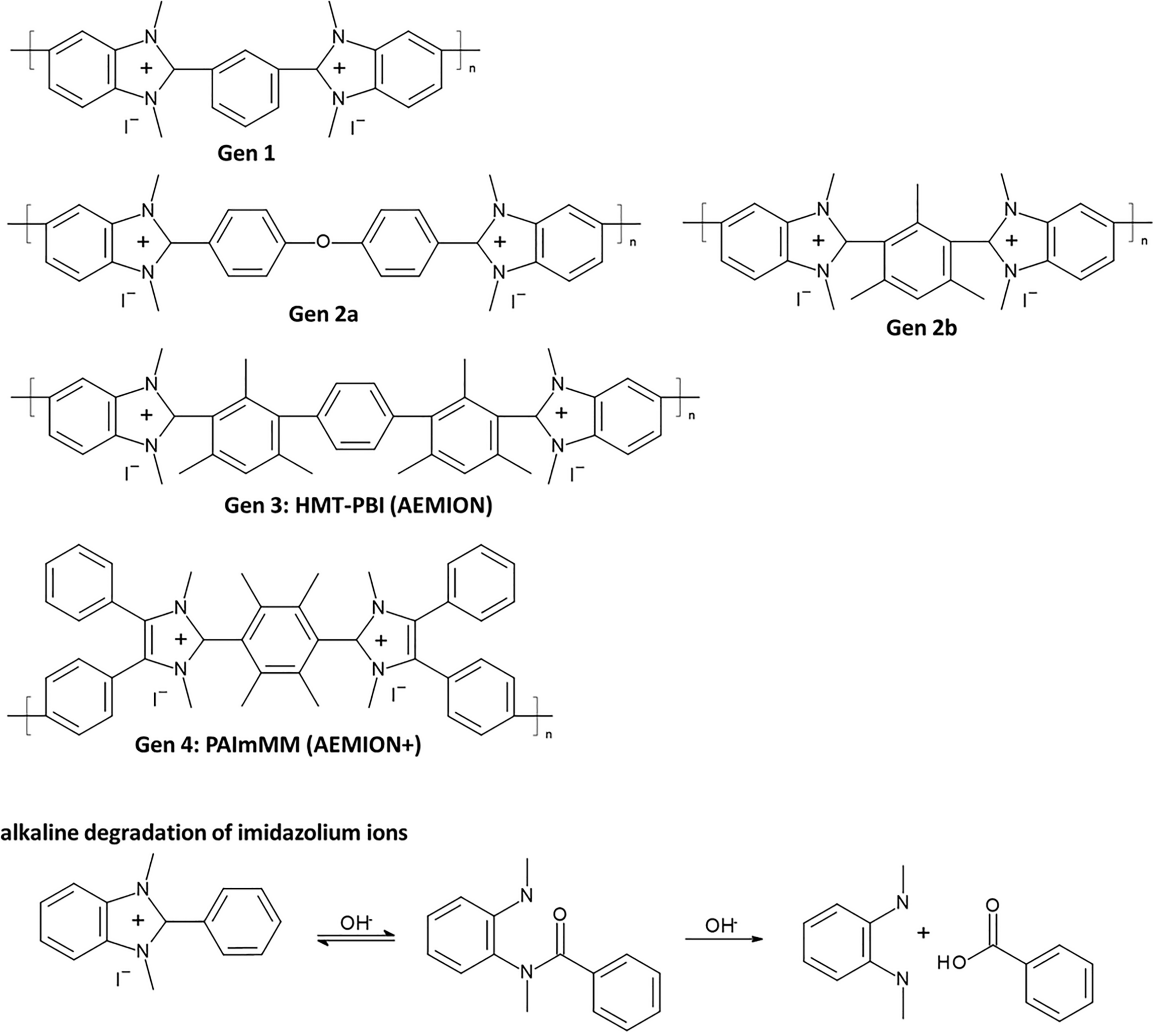
Development of polyimidazolium membranes and their main degradation pathway under alkaline conditions.

In 1993, Hu et al. reported the methylation of polybenzimidazole (PBI), which results in an ionene, i.e., a polymer which has charged groups as part of the polymer backbone (Figure 12, Gen 1).^140^ In 2011, Henkensmeier and Holdcroft independently reported the membrane properties of this material.^141,142^ Although methylated *m*-PBI forms strong, self-supporting AEMs, their hydroxide exchanged form rapidly disintegrates, because hydroxide ions react with the positively charged carbon atom in position 2, which results in imidazole ring opening followed by chain scission of the amide intermediate (Figure 12).^143^ To increase the alkaline stability, two strategies were investigated. One aimed to reduce the positive charge density on the C2 position by separating the charged moieties (Gen 2a).^144,145^ In another strategy, steric hindrance of the hydroxide attack was achieved by introducing methyl groups in the two *ortho* positions of the linking phenyl ring (Gen 2b). Because the phenyl ring and the imidazole ring prefer a nonplanar, nearly vertical orientation, one methyl group is located above the C2 position, the other below.^99,146^ This was a breakthrough and defined a new state of the art, for now alkaline stable AEMs were available for the first time. In a next development, the Holdcroft group developed HMT-PBI (Gen 3), which combines the two described strategies: (1) electronic stabilization of the charged system and (2) steric protection of the C2 position.^147^ Cross-linking with dichloroxylene conveniently allows to adjust the swelling without reducing the number of ionic sites.^148^ Membranes are produced by combining the unmethylated precursor polymer and the cross-linker and casting them into a membrane, which then is transferred into the ion conducting form by immersion in a solution which contains the alkylation reagent.^148,149^ The HMT-PBI technological platform was transferred to a newly founded company, IONOMR, which commercializes the membranes under the trade name AEMION. The newest generation of AEMION membranes (AEMION+) is a poly(bis-arylimidazolium) (Gen 4),^150^ which is claimed to have a half-life in 10 M KOH at 100 °C of >5000 h.^119^ Although the exact structure of AEMION+ is not disclosed and polymers with *N*-butyl groups would be more stable than with *N*-methyl,^150^ the degree of methylation of AEMION+ was mentioned in a recent work,^151^ indicating that PAImMM could be correct. Considering the trade-off between conductivity (highest with methylated nitrogen) and stability (highest with butyl), mixed methyl/butyl imidazolium ions (PAImMB) could be the most attractive structure.

**Figure 13:**
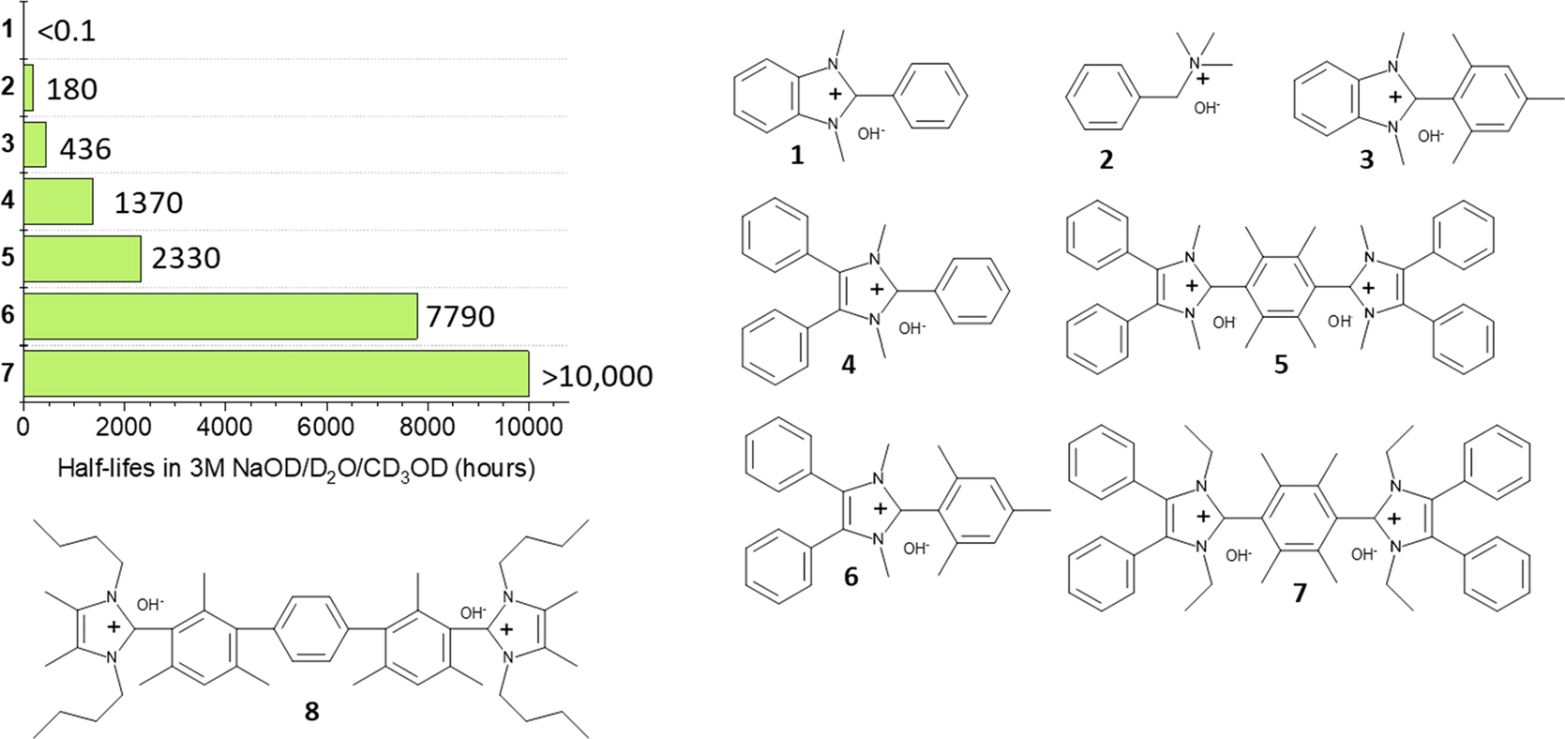
Half-lives of cationic model compounds in 3 M NaOD/D_2_O/CD_3_OD.^150^

A stability study confirmed the results from Marino and Kreuer^130^ that dimethylated 2-phenylbenzimidazolium hydroxide (**1**) is much less stable than benzyltrimethylammonium hydroxide (**2**) (Figure 13).^150^ Steric protection of the C2 position in **3** increases the lifetime above that of **1**. Long and Pivovar showed that imidazolium ions with methyl groups in position 4 and 5 have an enhanced stability and predicted that larger substituents could be even more stable.^152^ Consecutively, Hugar et al. investigated 4,5-diphenylimidazolium and reported a high stability, although 4,5-dimethyl imidazolium was slightly more stable, probably because of the opposite inductive effects of phenyl (electron withdrawing) and methyl (electron donating).^153^**6** is more stable than **5**, because the linking phenyl ring has to stabilize two positive charges in **5**. Changing *N*-methyl to *N*-ethyl further increases the stability, presumably a combined effect of steric protection and larger inductive effect (**5** < **7**). Hypothetically, a polymer containing the motif **8**, made by substituting the linking phenyl ring in **7** for a hexamethylated terphenyl (as in HMT-PBI, AEMION), changing the phenyl rings in positions 4 and 5 to methyl groups and the *N*-methyl groups to *N*-butyl groups could promote alkaline stability far beyond state-of-the art, but a polymer with that structure has not been realized yet.

Phase separation of (a) polymer backbones into mechanically stabilizing hydrophobic domains and (b) ionically functionalized side chains into ion conducting hydrophilic domains is a key feature of many membranes, and is especially pronounced for perfluorinated membranes like Nafion.^154^ Because the positively charged moieties of HMT-PBI are part of the backbone, separation of hydrophilic and hydrophobic polymer moieties into domains is hindered. However, the bulky, rigid nature of the polymer chains prevents efficient chain packing, and dry membranes have about 10% unoccupied free volume, which forms a percolating system. With increasing humidity, first the free volume is filled with water, then the polymer chains are forced apart.^155^ This allows high hydroxide conductivities, for example 100 mS/cm at 40 °C, 90% relative humidity, and even higher values should be seen if tested in liquid water.^156^

HMT-PBI membranes show excellent alkaline stability, high conductivity and very competitive mechanical properties (e.g., tensile strength of 50 MPa and Young’s modulus of 76 MPa and elongation at break of 940% for a dry, chloride form HMT-PBI membrane^147^) and perform well in electrolyzer systems. In one work, binder-free noble-metal free electrodes were prepared by plasma-spraying Ni, Al, and Mo on a porous substrate, and a performance of 2.086 V at 2 A cm^–2^ with 1 M KOH feed solution and 60 °C operation temperature was achieved.^157^ In another work, four AEMION membranes (AF1-HNN8-25, AF1-HNN8-50, AF1-HNN5-25, and AF1-HNN5-50; IEC is 1.4–1.7 mequiv OH/g for the 5er series and 2.1–2.5 mequiv OH/g for the 8er series; last digits indicate the thickness) were tested at the same conditions but with Pt/C and Ir black as catalyst and FAA3 ionomer as binder. The optimized performance of 1.82 V at 2 A cm^–2^ was obtained with the AF1-HNN8–50 membrane. Impedance analysis before and after 17 h of operation revealed that the main reason for increasing cell voltage was severe catalyst layer degradation.^158^ Khataee et al. tested the same membranes for 100 h. They reported a very minor, not quantifiable membrane degradation within this time but also reported that the membrane disintegrated when immersed in 2 M KOH at 90 °C for 1 month.^159^ This indicates that HMT-PBI membranes are not fully suitable for use in long-term operation.

Ionomr’s newest generation membrane type, the PAImMM-based AEMION+ membrane AF2-HWP8–75-X (75 μm thick, un-cross-linked, reinforced with a woven polyolefin support, IEC 2.3–2.6 mequiv/g) was tested in combination with IrO_*x*_ and Pt/C as catalysts and Nafion as binder. With 1 M KOH feed, a 5 cm^2^ cell operated at 80 °C reached a performance of about 1.9 V at 2 A cm^–2^.^151^ In another cell setup (50 cm^2^, apparently binder-free, commercial expanded mesh catalyst coated electrodes which are not disclosed in detail, 1 M KOH, 70 °C), the electrolyzer was operated for 8900 h at 200 mA cm^–2^. Most remarkably, the voltage remained in the range of 2 V, with a voltage increase rate of 18 μV h^–1^. In another test, the cell design was altered to eliminate effects of the Ni porous transport layer on cell voltage. A 5000 h test at 600 mA cm^–2^ and an initial voltage of about 1.9 V showed a voltage increase rate of just 13 μV h^–1^. H_2_ crossover remained <0.4%.

The superior alkaline stability of Aemion+ over Aemion seen during elecytrolyzer operation is also revealed by their ex-situ stabilities in 3 M KOH at 80 °C. While the chloride conductivity of Aemion dropped 72% within 7 days (along with a drop of IEC), Aemion+ retained 61% of its chloride ion conductivity, and no drop in IEC was observed.^160^

### 3.4 Polymers and Membranes Prepared by Polyhydroxyalkylations

**Figure 14:**
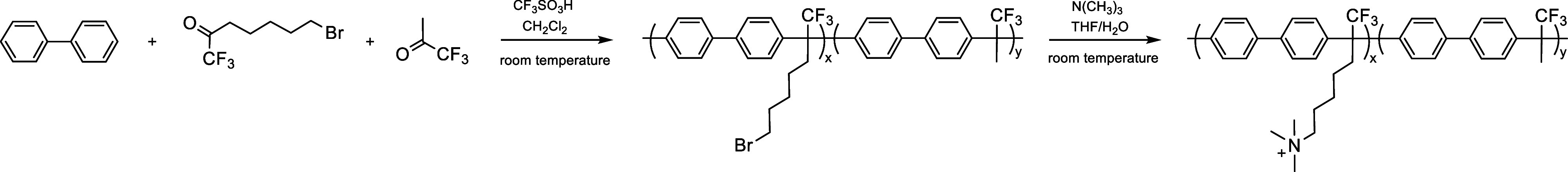
Preparation of poly(biphenyl alkylene)s by polyhydroxyalkylation and quaternization carried out by Bae et al.^169^

**Figure 15:**
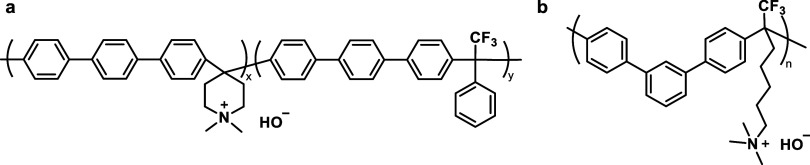
PiperION by Versogen (a) and Orion CMX by Orion polymer (b) are two commercial AEMs prepared by polyhydroxyalkylations.

As already mentioned above, the early development of AEMs for alkaline applications focused almost exclusively on aromatic polymers, such as poly(ether sulfone)s and poly(ether ketone)s, often functionalized by benzyltrimethylammonium (BTMA) cations.^112,118,161−163^ However, it was soon discovered that membranes based on these polymers readily degrade under alkaline conditions, mainly because the ether links are highly activated for aqueous hydrolysis by the presence of the strongly electron-withdrawing sulfone and ketone groups, respectively, and possibly by nearby quaternary ammonium (QA) cations.^114,164−168^ For example, Arges and Ramani demonstrated that BTMA cations attached close to ether bonds in polysulfones trigger fast alkaline degradation of the polymer backbone through both quaternary carbon and ether hydrolysis.^114^ Largely in response to the poor alkaline stability of the ether-containing polymers, Bae and co-workers in 2015 for the first time synthesized a series of high molecular weight cationic poly(arylene alkylene)s by superacid-mediated polyhydroxyalkylation of biphenyl and a mixture of two ketones, followed by quaternization (Figure 14).^169^ This breakthrough study demonstrated the excellent properties of these aryl ether-free polymers as AEMs, including high alkaline stability and conductivity. Following the initial report by Bae et al., a large variety of aromatic polymers have been prepared by polyhydroxyalkylations, and these polymers have strongly emerged as a class of durable high performance membrane materials for alkaline water electrolyzers, as well as for other electrochemical energy systems such as fuel cells and flow batteries.^163^ So far, two different kinds of AEMs prepared by polyhydroxyalkylations have been commercialized, namely PiperION by Versogen and Orion CMX by Orion Polymer (Figure 15).

**Figure 16:**
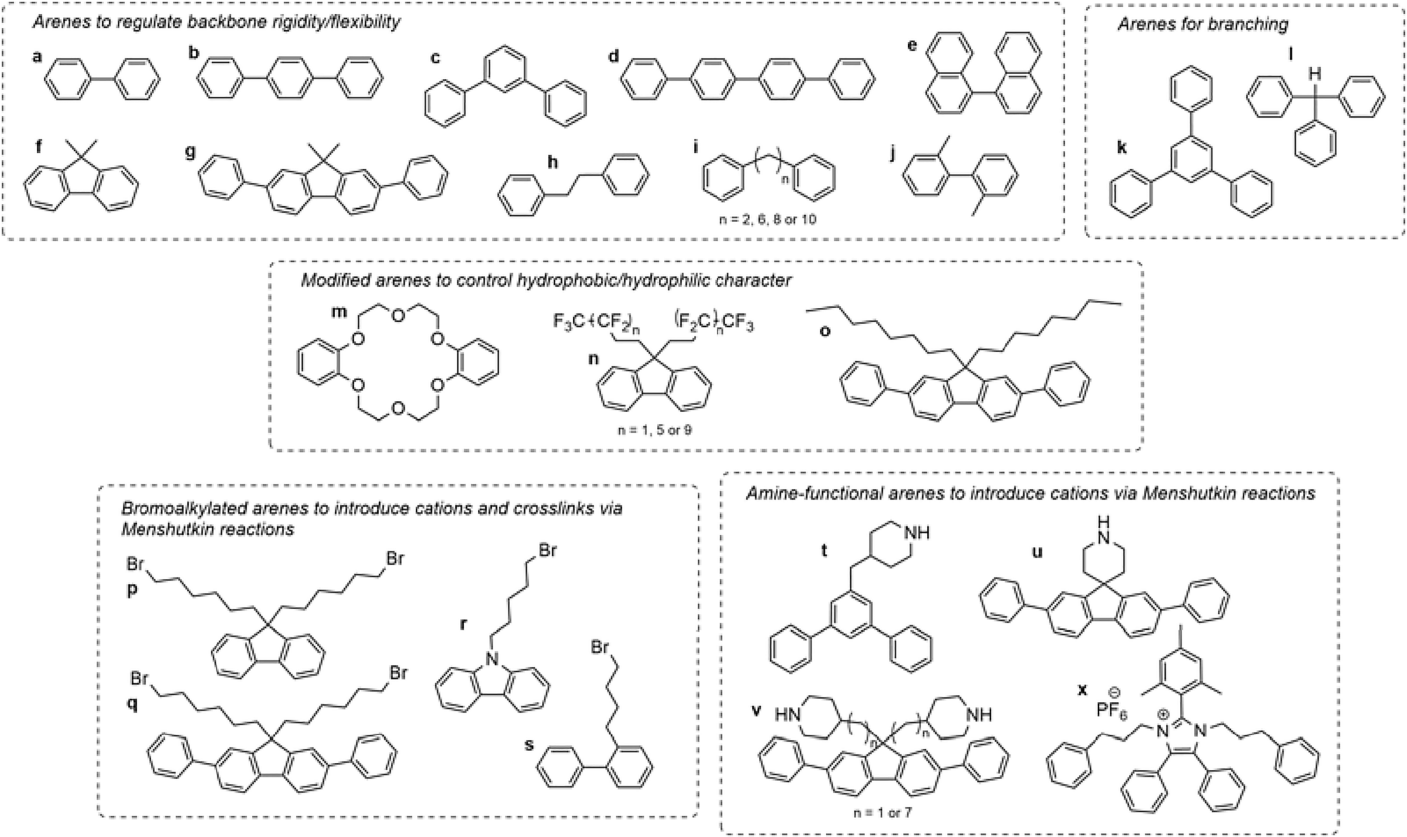
Examples of arene monomers with different functionalities used in polyhydroxyalkylations to prepare AEM materials.

**Figure 17:**
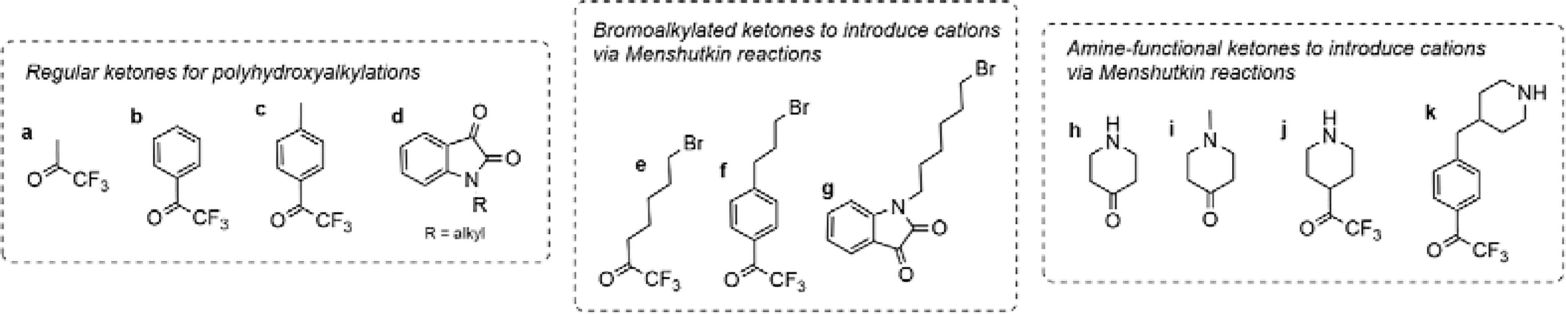
Examples of ketone monomers with different functionalities used in polyhydroxyalkylations to prepare AEM materials.

Polyhydroxyalkylations are Friedel–Crafts type polycondensations where generally an electron-rich arene compound reacts with a suitable ketone or aldehyde in a superacidic medium to form a molecularly rigid aromatic polymer without any heteroatoms or benzylic hydrogens in the backbone structure, which generally provides a very high alkaline stability.^170^ Moreover, the aldehyde or ketone monomer forms regular alkylene units with sp^3^-hybridized carbon atoms along the backbone, which provides chain flexibility to the backbone that in turn facilitates polymer solubility and membrane processability. A large number of commercially available arene (Figure 16) and ketone (Figure 17) compounds are suitable as monomers, and an even larger number of compounds can be readily synthesized and used in polyhydroxyalkylations. This provides great flexibility when it comes to tuning properties such as backbone flexibility and ionic content of the polymers and AEMs by the monomer choice and by copolymerization of monomer mixtures.^163,170^ In addition, the polymers can be modified after the polymerization by, e.g., introducing different cationic groups or by covalent cross-linking, as discussed in more detail below. Hence, the polymers are usually prepared to contain either alkyl halide or amine (secondary or tertiary) groups which are subsequently used to introduce the ammonium cations through Menshutkin reactions with amines and alkyl halides, respectively. The same reactions can also be used to introduce covalent cross-links by using corresponding difunctional compounds. Another important feature of the polyhydroxylalkylation reaction is that polymers with very high molar masses can be obtained after careful tuning and optimization of the conditions. To reach a sufficiently high molar mass is important for the film forming ability of the polymer and the viscoelastic properties of the AEM, which to a large degree controls the mechanical properties and water uptake, especially at high ionic contents.

Hydroxyalkylation reactions between different carbonyl compounds and electron rich arenes are since long widely used for industrial production of different chemicals, such as bisphenol A which is formed in a reaction between acetone and phenol catalyzed by a strong acid. The chemical applicability of the hydroxyalkylation reaction was significantly widened by the work on superelectrophilic activation by Nobel prize winner George Olah and his co-workers, who demonstrated that the reactivity of some specific electrophiles increased significantly when in contact with Brønsted or Lewis superacids by the formation of protonated and highly reactive “superelectrophiles”.^171^ This breakthrough chemistry enabled high-yield and specific hydroxyalkylations of less reactive arenes^172^ and the exploration of polyhydroxyalkylations to prepare polymers, which was first demonstrated by Zolotukhin et al.^173−176^ The latter research group has since continued to further investigate and develop the polyhydroxyalkylation reaction.^177−186^

**Figure 18:**
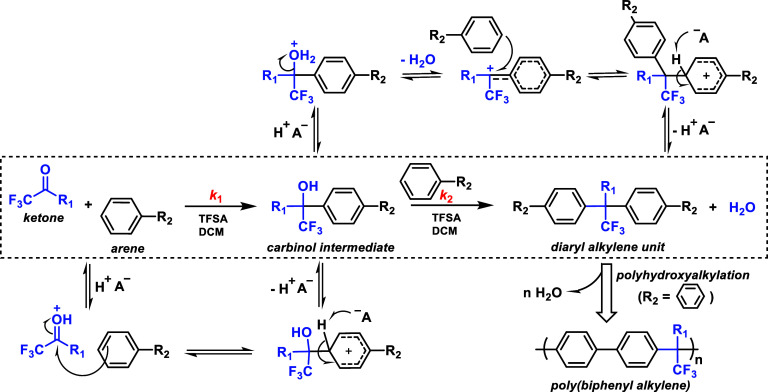
Principal mechanism of the Friedel–Crafts type hydroxyalkylation reaction exemplified with a trifluoromethyl ketone and an arene monomer. If R_2_ = Ph, the corresponding polyhydroxyalkylation results in a poly(biphenyl alkylene) in which R_1_ may potentially contain a cation or a group that can be transformed into a cation. (H^+^A^–^ = TFSA = triflic acid, DCM = dichloromethane).

In order to develop new high-performance electrolyzer AEM materials by polyhydroxyalkylations, it is useful to consider the mechanism behind this reaction to, for example, promote the formation of high-molar mass polymers and depress side reactions.^168,169^ The general mechanism is outlined in Figure 18, where a trifluoromethyl ketone reacts with an electron-rich arene in two steps to form a diarylalkylene product in a mixture of trifluoromethanesulfonic acid (TFSA) and dichloromethane (DCM).^180^ Each step is associated with a rate constant, *k*_1_ and *k*_2_, respectively.^180^ In the first step, the trifluoromethyl ketone is protonated by the superacid (TFSA) to produce a highly reactive superelectrophile, which then reacts with the arene to yield a carbinol intermediate. Subsequently, the intermediate is protonated to form a tertiary carbocation after the formation of water, which in turn condenses with a second arene to form the diarylene alkylene product. By using a difunctional arene monomer (e.g., biphenyl with R_2_ = Ph), a poly(arylene alkylene) is produced. The rate constants *k*_1_ and *k*_2_ depend strongly on the monomers used and the reaction conditions applied.^180^ For example, the *k*_1_/*k*_2_ ratio for the reaction between 1,1,1-trifluoroacetone and anisole in TFSA is 350 (*k*_1_ > *k*_2_), but with benzene as nucleophile the ratio is 10^–4^ (*k*_1_ ≪ *k*_2_).^180^ The ratio can also be tuned by changing the acidity of the reaction solution. If the acidity is increased, *k*_1_ will decrease and *k*_2_ remains largely unchanged so that the ratio decreases.^180^ Hence, by careful reaction design either a mono- or a diarylated product can be obtained. In addition, if *k*_1_ < *k*_2_, the first step will be rate determining, meaning that the polyhydroxyalkylation reaction can be accelerated by nonstoichiometric conditions.^180^ This is rare for polycondensation reactions, which generally require strict stoichiometric conditions to reach high molar masses. With an excess of the carbonyl monomer, the polymerization rate will increase with increasing molar imbalance between the monomers. Zolotukhin and co-workers have shown that with a 15–20% excess, the reaction rate increases dramatically to complete polymerizations within minutes.^180^ Yet, if the molar excess of the carbonyl monomer is raised too high, side reactions may emerge to result in cross-linking of the product.

#### 3.4.1 Membranes Based on Poly(Arylene Piperidinium)

Two of the most employed ketones in the preparation of AEMs by polyhydroxyalkylation are 4-piperidone and *N*-methyl-4-piperidone, forming the basis for the poly(arylene piperidinium) class of membrane polymers. Both these monomers are commercially available and generally highly reactive in polyhydroxyalkylations with arenes such as biphenyl and terphenyls. This enables a straightforward pathway to rigid ether-free aromatic polymers functionalized with secondary and tertiary piperidine groups, respectively. Klumpp et al. have demonstrated that different piperidones can react with arenes to form diarylpiperidines in good to excellent yields (80–99%) in mixtures of TFSA and dichloromethane.^187^*N*-Substituted 4-piperidones were found to produce the highest yields. Reactions between mono- and diphenylated piperidone, respectively, with chlorobenzene indicated that at least one of the electrophilic aromatic substitution steps was reversible. Consequently, both reactions resulted in a mixture of three products, i.e., diphenyl piperidine, dichlorophenyl piperidine and chlorophenyl–phenylpiperidine.^187^ Zolotukhin et al. originally investigated the polyhydroxyalkylation of piperidone and electron-rich arene monomers to produce poly(arylene piperidine)s.^178^

**Figure 19:**
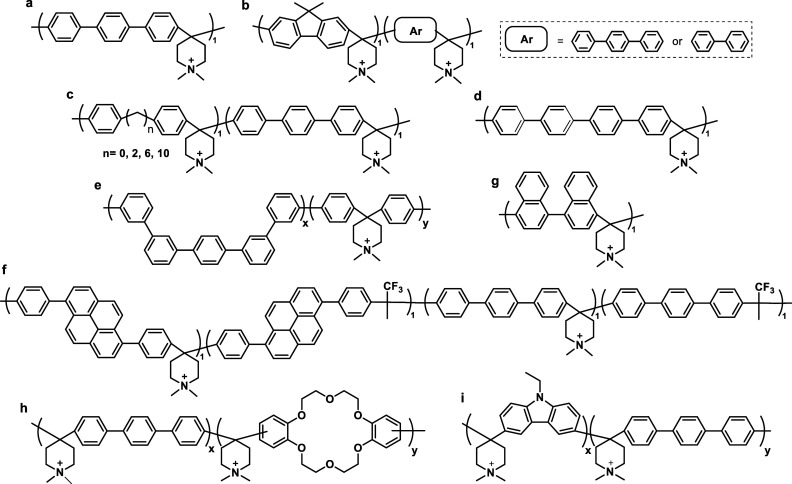
Examples of poly(arylene piperidinium)s with different arylene groups: (a) *p*-terphenyl, (b) 9,9-dimethylfluorene, (c) α,ω-diphenyl alkylene, (d) *p,p*-quaterphenyl, (e) *m,p,m*-quinquephenyl, (f) 1,6-diphenylpyrene, (g) 1,1′-binaphthyl, (h) dibenzo-18-crown-6, and (i) 9-ethylcarbazole.

The first reported poly(arylene piperidinium)s targeted for AEMs were prepared in TFSA-mediated polyhydroxyalkylations of *N*-methyl-4-piperidone and bi- and *p*-terphenyl, respectively, by Jannasch et al. (Figure 19a).^188^ After quaternization, AEMs with dimethylpiperidinium cations displayed high alkaline stability with a mere 5% ionic loss at 90 °C in 2 M aq. NaOH after 15 days. Longer *N*-alkyl tethers (C4–C8) strongly promoted Hofmann ring-opening elimination reactions. The AEMs reached a hydroxide conductivity of 89 mS cm^–1^ at 80 °C.^188^ In a parallel study, Yan et al. studied poly(arylene dimethylpiperidinium)s prepared using *N*-methyl-4-piperidone, 2,2,2-trifluoroacetophenone, and bi- and *p*-terphenyl, respectively, (Figure 15a) where the IEC was tuned by the ratio of the two ketones.^189^ AEM based on these high molar mass polymers displayed excellent mechanical properties and maintained their conductivity and flexibility after 1,000 h in 1 M aq. KOH at 100 °C, and a *p*-terphenyl based AEM reached 193 mS cm^–1^ at 95 °C.^189^ This work formed the basis for the commercial PiperION membrane. Concurrently, Zhuang et al. independently prepared and studied poly(*p*-terphenyl piperidinium) AEMs and reported an ionic loss of only 5% over 210 days in 1 M NaOH at 80 °C, and a hydroxide conductivity of 137 mS cm^–1^ at 80 °C.^190^

After these initial and encouraging results, a large number of studies have focused on structure–property relationships of copolymers, and on the control of AEM properties by, e.g., tuning the IEC and backbone flexibility. For example, Lee and co-workers carried out polyhydroxyalkylations of dimethylfluorene and *N*-methyl-4-piperidone with either biphenyl or *p*-terphenyl (Figure 19b).^191^ AEMs based on these copolymers showed high hydroxide conductivity (208 mS cm^–1^ at 80 °C), low H_2_ permeability, and a 2,000 h durability in 1 M NaOH at 80 °C. The same research group later reported on electrolyzer results obtained with poly(fluorenyl-*co*-aryl piperidinium)-based anhydrous cathodes (only fed by water produced at the anode) and an anode fed with 2 M aq. KOH solution.^192^ Using platinum-group-metal (PGM) catalysts, the system achieved a current density of 7.68 A cm^–1^ at 2 V, while reaching 1.62 A cm^–1^ at 2 V with non-PGM catalysts. In both cases, the systems operated under 0.5 A cm^–1^ at 60 °C during more than 1,000 h.^192^ With the aim to reduce the phenyl content, the phenyl adsorption to catalysts, and backbone rigidity, Lee et al. have also prepared copolymers with 1,2-diphenylethane (bibenzyl) (Figure 19c [*n* = 2]) and found that the water content increased with the diphenylethane content.^193^ This enabled the copolymers to be tuned for high water contents for use as ionomer in the cathode and, alternatively, for moderate water uptake, low H_2_ permeability, and high dimensional stability and conductivity for use as AEMs.^193^ Using microporous polyethylene substrates, Lee et al. have also prepared mechanically reinforced composite membranes based on their dimethylfluorene- and diphenylethane-based copolymers to significantly enhance the tensile strength and elongation at break.^194,195^

Zhu et al. have compared poly(arylene piperidinium)s and AEMs prepared with *p*-terphenyl, *m*-terphenyl, or a mixture of biphenyl and *p*-terphenyl.^196^ All the AEMs had morphologies with seemingly well-connected microphase domains and a high and similar alkaline stability.^196^ Overall, the AEM based on *m*-terphenyl gave the most favorable properties, including a conductivity of 144 mS cm^–1^ at 80 °C. In studies of poly(terphenyl piperidinium)s with different compositions of *p*- and *m*-terphenyl units, Kim et al. found that a copolymer with a 50–50 composition gave AEMs with the highest conductivity (130 mS cm^–1^ at 80 °C), the lowest swelling and the best mechanical properties, at least partly due to a well-defined cocontinuous morphology.^197^

Further experiments have demonstrated the possibilities of using poly(arylene piperidinium) AEMs for water electrolysis applications. He and co-workers have reported a current density of 1,064 A cm^–1^ at 2.5 V and 50 °C for an electrolyzer cell with Pt/IrO_2_ cathode and Ni-foam anode fed with 1 M aq. KOH solution operating with an poly(*m*-terphenyl piperidinium)-based AEM.^198^ The high frequency resistance was 0.165 Ω cm^2^ at 1.8 V, and the voltage remained at 2.1 V during more than 500 h at 200 mA cm^–2^. In another piece of work, Mustarelli et al. investigated high-IEC poly(biphenyl piperidinium) AEMs with thicknesses between 15 and 60 μm in an electrolysis cell with Pt cathode and Ni-foam anode, fed with 1 M aq. KOH solution.^199^ The performance with the thinnest AEM (2.8 A cm^–1^ at 2.2 V and 60 °C) was found to exceed that of the commercially available PiperION membrane under the same conditions. However, the H_2_ crossover was not measured and can be expected to be high for the former AEM. The Yan group fabricated a Ni foam anode with vertically aligned fluoride-incorporated oxyhydroxide nanosheets, which was integrated with a poly(arylene piperidinium) AEM in a water-fed electrolyzer cell.^200^ The cell achieved a performance of 1,020 mA cm^–2^ at 1.8 V and 90 °C. Kim et al. have employed poly(biphenyl piperidinium) AEMs and poly(fluorene biphenyl indole) PEMs to assemble cells with bipolar membranes which operated steadily below 1.5 V in the current range 0–1.5 A cm^–2^.^201^ The best-performing cell reached a voltage of 0.75 V at 700 mA cm^–2^ and 70 °C.

##### 3.4.1.1 Alternative Arene Monomers

Several research groups have investigated the use of alternative arene monomers to modify the backbone structure by extending the aromatic sequence,^202,203^ introducing aliphatic segments,^204^ incorporating fused aromatic rings^205,206^ and by introducing hydrophilic heteroatom groups,^177−181^ respectively, in order to tailor the properties of poly(arylene piperidinium) AEMs and to introduce new functionalities. For example, Li et al. prepared a poly(*p*-quaterphenyl piperidinium) with regularly spaced cations along a rigid backbone (Figure 19d), and compared the properties with copolymers based on bi- and *p*-terphenyl, respectively, having more flexible backbone with randomly spaced cations, all at the same IEC value.^202^ AEMs based on the former polymer showed a pronounced microphase separated morphology, high conductivity (119 mS cm^–1^ at 80 °C), and an ability to be cast into mechanically strong thin membranes (4 μm). An electrolyzer cell assembled with the quaterphenyl membrane reached a current density of 1,544 mA cm^–2^ at 2 V and 85 °C with circulating 1 M aq. KOH solution, a factor 1.3–1.4 times higher than when the copolymer AEMs were used.^202^ However, running at 200 mA cm^–2^ at 60 °C, a performance loss of 0.11 mV h^–1^ was observed over 402 h, which could be attributed to ionic loss by Hofmann β-elimination and nucleophilic substitution reactions. Ding and co-workers further extended the aromatic segment and prepared poly(quinquephenylene-*co*-diphenylene piperidinium) (Figure 19e) AEMs with high chemical stability and mechanical strength.^203^ Following a different synthetic strategy, Lee et al. have investigated the influence of an extended flexible aliphatic segment and prepared copolymers based on *p*-terphenyl and diphenylalkylenes with different numbers of methylene units (*n* = 0, 1, 2, 6, and 10) in-between the phenyl groups (Figure 19c).^204^ They found that the corresponding AEMs had high alkaline and oxidative stability and that long alkyl chains (*n* = 6, 10) improved the dimensional stability and the H_2_ barrier properties, while short alkyl chains favored conductivity (>150 mS cm^–1^ at 80 °C).

Xu and co-workers prepared a diphenylpyrene monomer and prepared copolymers with *p*-terphenyl, methylpiperidone, and 1,1,1-trifluoroacetone (Figure 19f).^205^ The presence of π–π stacking of the pyrene units was verified by X-ray scattering, and these polymers produced AEMs with high mechanical strength, dimensional stability and a conductivity reaching 153 mS cm^–1^ at 90 °C. Liu et al. instead used 1,1′-binaphthyl in a polyhydroxyalkylation with methylpiperidone and reported that AEMs based on the resulting poly(binaphthyl piperidinium) (Figure 19g) had a high alkaline stability and a distinct microphase separation leading to higher conductivity (135 mS cm^–1^ at 80 °C) than a corresponding AEM based on quaterphenyl.^206^

Li et al. incorporated hydrophilic crown ether units in the backbone polymer by polyhydroxyalkylation of dibenzo-18-crown-6, *p*-terphenyl, and methylpiperidone (Figure 19h).^207^ The crown ether units induced a high water uptake which promoted conductivity and alkaline stability of the AEMs in comparison with corresponding polymers without crown ethers. A water electrolyzer cell based on the crown ether AEM displayed a current density of 2,000 mA cm^–1^ at 2.1 V with circulating 1 M aq. NaOH solution at 80 °C.^207^ Still, the presence of the alkyl ether units raises questions regarding the long-term stability of these materials. Going against the trend of aryl ether-free backbones, He and co-workers prepared copolymers using the reactive and low-cost diphenyl ether monomer, and reported solubility in acetone–water mixtures and AEMs with good alkaline stability and conductivity (88 mS cm^–1^ at 80 °C).^208^ Still, the presence of the diphenyl ether units may potentially lead to chain scission under harsh alkaline conditions. Copolymer AEMs based on *N*-ethylcarbazole, *p*-terphenyl, and methylpiperidone (Figure 19i) were synthesized and studied by Wei et al.^209^ They reported high conductivity (205 mS cm^–1^ at 90 °C) and mechanical strength, low H_2_ permeability, as well as high alkaline stability. In addition, different copolymers based on isatin and methylpiperidone have also been investigated.^209,210^ For example, Li et al. prepared copolymer AEMs based on isatin, *p*-terphenyl, and *N*-methylpiperidone.^211^ An electrolyzer cell fed with 1 M aq. NaOH solution recorded a current density of 910 and 1,000 mA cm^–2^ at 55 and 75 °C, respectively, at 2.2 V. However, after 120 h operation at 400 mA cm^–2^ and 55 °C, a significant cationic loss by Hofmann β-elimination and nucleophilic substitution was observed.^201^

In conclusion, a wide variety of different arene monomers can be employed to efficiently tune polymer chain flexibility and the IEC of the AEM. The former parameter typically influences polymer solubility, membrane formation, and the mechanical properties. In addition, the nature of the arene monomer may also control the level of microphase separation in the AEM, e.g., long monomers will introduce blockiness and monomers with fused aromatic rings may give rise to π–π stacking.

##### 3.4.1.2 Modified Piperidinium-Based Cations

**Figure 20:**
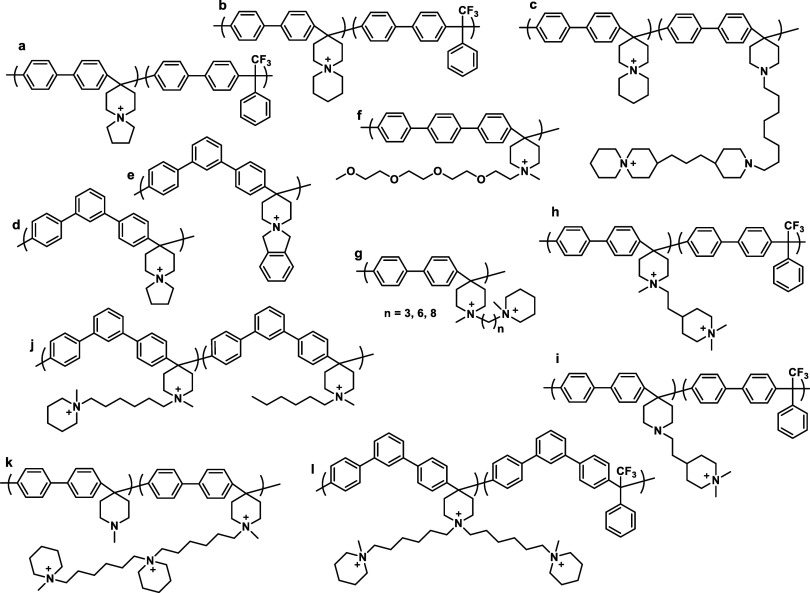
Examples of poly(arylene piperidinium)s with modified piperidinium cations: (a–e) *N*-spirocyclic cations, (f) *N*-tethered hydrophilic side chain, and (g–l) various *N*-tethered cationic side chains.

Poly(arylene piperidine)s prepared from piperidone have been cycloquaternized (cycloalkylated) by using dihaloalkyls to introduce *N*-spirocyclic cations.^212−215^ For example, Jannasch and co-workers carried out cycloquaternizations of poly(biphenyl piperidine)s using 1,4-dibromobutane and 1,5-dibromopentane to introduce spirocyclic 5-azoniaspiro[4.5]decane (ASD) (Figure 20a) and 6-azoniaspiro[5.5]undecane (ASU) (Figure 20b) cations, respectively.^212^ At 80 °C, the AEMs reached ∼100 mS cm^–1^ and degradation studies showed that the ring directly attached to the backbone was more sensitive toward Hofmann β-elimination than the outer ring of the spiro-cations. Zhu et al. could improve dimensional stability by preparing cross-linked polymers containing ASU cations, but this led to decreased alkaline stability.^213^ Zhu and co-workers further synthesized biphenyl polymers with ASU cations attached both directly to the backbone and via long flexible alkyl spacers (Figure 20c) and found that the presence of the latter cations led to enhanced microphase separation and increased conductivity, up to 117 mS cm^–1^ at 80 °C.^214^ Lammertink et al. studied *m*-terphenyl-based polymers functionalized with ASD-type cations (Figure 20d and e) and found that increasing the size of the cationic group decreased the water uptake but concurrently led to both reduced conductivity and alkaline stability.^215^

Several research groups have prepared and studied poly(arylene *N*-methylpiperidine)s quaternized with hydrophilic^216−218^ nonionic alkyl halides and with alkyl halides containing one^219−228^ or more^229−231^ quaternary ammonium groups to introduce additional cations on side chains to increase the IEC. For example, Li and co-workers introduced hydrophilic nonionic *N*-tethered tetra(ethylene oxide) pendants and found improved ex-situ alkaline stability in comparison with a regular poly(*p*-terphenyl *N*,*N*-dimethylpiperidinium) control AEM (Figure 20f).^216^ In addition, the oligo(ethylene oxide) chains crystallized in the AEM during casting, which enhanced the hydrophilic–hydrophobic phase separation, leading to enhanced conductivity. An electrolysis single-cell (IrO_2_ anode, PtRu/C cathode) fed with 1 M aq. KOH solution at 80 °C revealed a current density of 1.1 A cm^–1^ at 2.0 V, which was lower than for the control AEM (5.6 A cm^–2^, 2.0 V), mainly because of the lower high-frequency resistance at high current density of the latter membrane.^216^ Zhu et al. studied high-IEC poly(biphenyl piperidinium) where each piperidinium ring carried an additional piperidium cation via an *N*-alkyl chain with 3, 6, and 8 methylene units, respectively (Figure 20g).^219^ The AEM with the hexyl chains showed the most pronounced microphase separation and highest water uptake, and also produced the highest conductivity (117 mS cm^–1^, 80 °C, IEC = 3.78 mequiv g^–1^). Jannasch and co-workers tethered poly(biphenyl *N*-methylpiperidine) and poly(biphenyl piperidine) with bromoalkylated *N*,*N*-dimethylpiperidinium and spirocyclic ASU cations, respectively, to obtain di- and monocationic moieties along the backbone (Figure 20h and i, respectively).^224^ Benefiting from a high local ionic concentration, AEMs with the dicationic moieties showed very high conductivity (170 mS cm^–1^, 80 °C), but suffered from limited alkaline stability of the piperidinium rings directly attached to backbone. AEMs with the monocationic moieties had a much higher alkaline stability and still reached high conductivity (131 mS cm^–1^, 80 °C).^224^ Zhang et al. have prepared poly(*m*-terphenyl piperidinium) *N*-tethered with both nonionic hexyl chains and hexyl chains with terminal piperidinium cations (Figure 20j) with the aim to promote ionic clustering and ion transport properties.^223^ An AEM grafted with 32% ionic chains and 8% nonionic chains showed the best conductivity (112 mS cm^–1^, 80 °C). When this AEM was employed in an electrolyzer cell fed with 1 M aq. KOH solution at 60 °C, with a nonplatinum group metal anode, a current density of 0.752 A cm^–2^ was recorded at 2.2 V.^223^

Liu et al.^230^ tethered long and flexible *N*-alkyl side chain containing two piperidinium cations onto poly(biphenyl piperidinium) (Figure 20k), and reported a conductivity of 156 mS cm^–1^ at 80 °C) and a conductivity loss of merely 5% after storage in 2 M aq. NaOH for 1080 h at 80 °C. Using a similar approach, Zhu and co-workers introduced long *N*-alkyl side chains with three quaternary ammonium cations into *N*-positions of poly(*m*-terphenyl piperidinium), and produced AEMs with distinct hydrophilic–hydrophobic phase separation that reached a slightly higher conductivity than the AEMs reported by Liu et al., 164 mS cm^–1^.^229^ Moreover, Ling et al. instead tethered each piperidinium ring in poly(*m*-terphenyl piperidinium) with two alkyl chains with terminal piperidinium cations, and produced AEMs with high alkaline stability which recorded 117 mS cm^–1^ at 80 °C (Figure 20l).^231^ In addition to attaching hydrophobic alkyl chains with cationic groups to piperidine rings, there are a few reports on the attachment of alkyl chains carrying terminal anionic (sulfonate) groups to form amphoteric membranes.^232,233^ In some cases cations have been tethered via hydrophilic side chains, i.e., di(ethylene oxide)^234,235^ and hydroxyl-containing^236,237^ side chains, to increase the size of the hydrophilic (ionic) phase domain to improve its percolation and conductivity.

To conclude, alkylation of the piperidine group offers a straightforward possibility to control and improve the alkaline stability by, e.g., forming spirocyclic cations. Alkylation strategies can also be employed to introduce two or more cations via the piperidine group in order to increase the IEC, and to increase the local ionic concentration in the AEM to favor ionic clustering, and thus the ionic conductivity.

##### 3.4.1.3 Introduction of Side Chains

**Figure 21:**
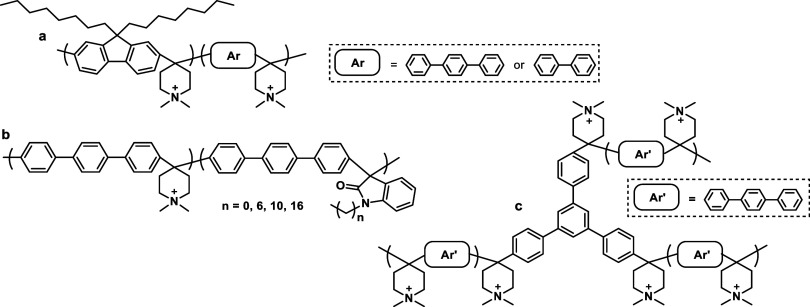
Poly(arylene piperidinium)s with alkyl side chains (a and b) and branching sites (c).

Tethering flexible hydrophobic side chains on the backbone, away from the piperidinium cations, is a convenient strategy to increase solubility and dimensional stability and enhance microphase separation of the AEM. This frequently leads to higher conductivities than for the corresponding nontethered materials, despite a higher IEC of the latter. These side chains are commonly introduced via alkylated monomers prepared in S_N_2 reactions of alkyl bromides with arenes and ketones containing acidic hydrogen atoms, such as fluorene,^238,239^ indole,^240^ and carbazole.^241^ Using molecular dynamics simulations of poly(*p*-terphenyl isatin-*co*-piperidinium) carrying hydrophobic nonionic alkyl side chains and hydrophilic alkyl side chains with terminal piperidinium cations, respectively, Fu et al. studied the mechanism of the hydroxide ion transport and the effect of the side chains in these AEMs.^242^ One of the main conclusions was that AEMs with side chains attached to the backbone separated from the cationic groups were superior ion conductors in comparison with AEMs with side chains directly linked to the piperidinium cations. Lee and co-workers have directly compared the effects of having hexyl and octyl side chains, respectively, attached to either piperidinium cations or to fluorene units (Figure 21a) in the backbone.^238^ The study showed that side chains tethered to the cations reduced the alkaline stability and the conductivity, whereas the side chains placed on the fluorene units brought improved dimensional stability, ionic conductivity (up to 134 mS cm^–1^), mechanical properties and electrochemical stability. Li et al. synthesized and investigated AEMs based on poly(arylene indole piperidinium) with different alkyl chains attached to the indole units (Figure 21b).^240^ They reported that the side chains induced a distinct microphase separation and that the highest conductivity (134 mS cm^–1^ at 80 °C) was reached by the decyl tethered AEM, despite having a lower IEC value than the nontethered control sample.

##### 3.4.1.4 Branching

An attractive and general approach to improve the mechanical properties of membranes is to introduce branching sites during the polymerization by adding a small balanced fraction of a multifunctional monomer. Adding too much will generally cause cross-linking and lead to insoluble products. Branching will in general enhance the average molar mass and increase the level of chain entanglement, both leading to improvements in dimensional stability and mechanical properties.^243^ When it comes to poly(arylene piperidinium)s, branching has been achieved by adding a few percent of 1,3,5-triphenylbenzene,^244−246^ triphenylmethane,^247^ or substituted fluorene^248^ as trifunctional branching agents. For example, Hu, Lee, and co-workers prepared branched poly(*p*-terphenylene piperidiniums) by adding 1–5 mol % triphenylbenzene during the polyhydroxyalkylation (Figure 21c).^244^ An AEM prepared with 2.5% branching agent showed the best properties, including a high tensile strength and elongation at break (>60 MPa and >35%, respectively) a high conductivity (145 mS cm^–1^ at 80 °C) and high alkaline stability (intact after 1500 h in 1 M KOH at 80 °C).

##### 3.4.1.5 Cross-linking

**Figure 22:**
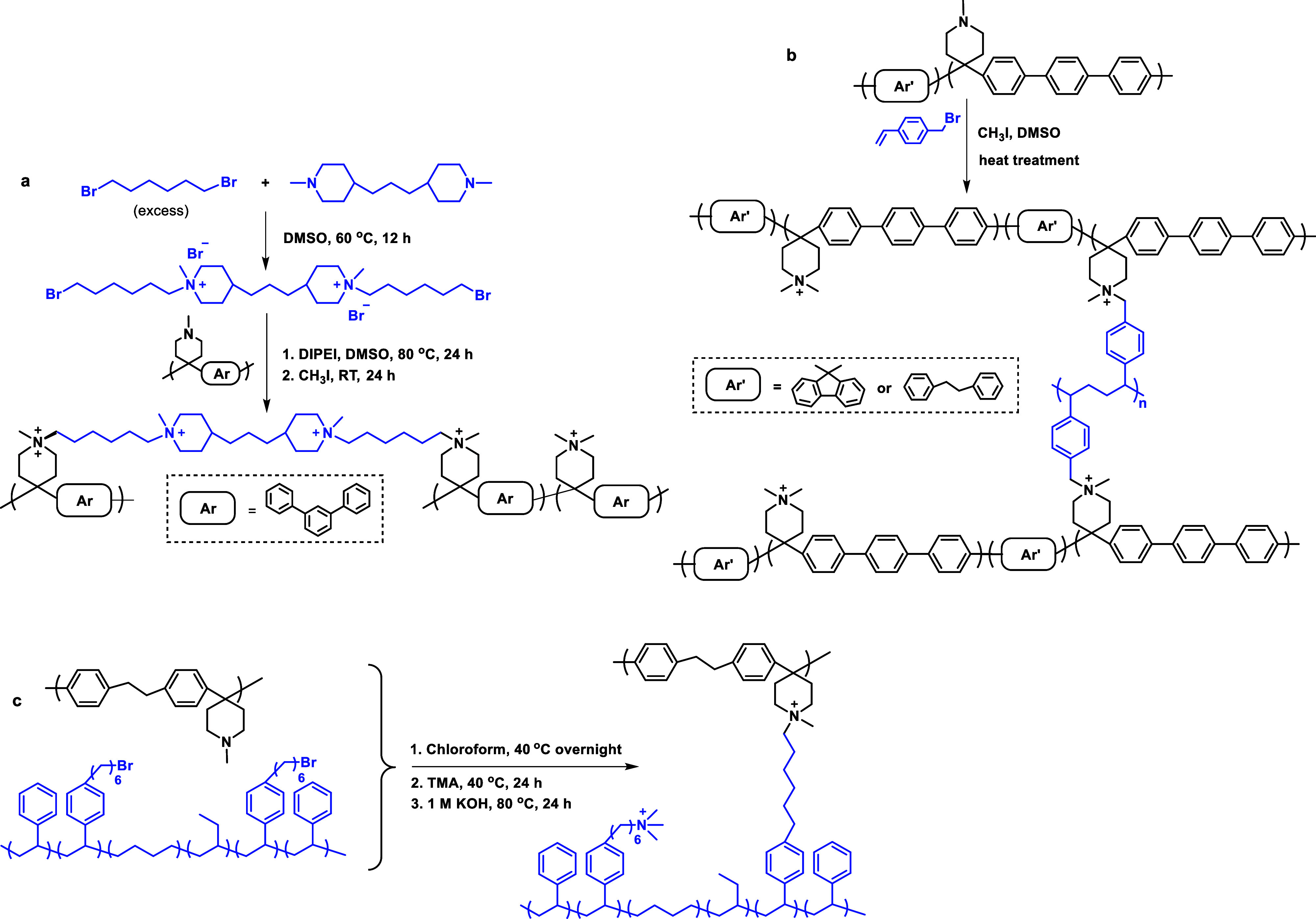
Poly(arylene piperidinium) AEMs cross-linked by (a) multicationic cross-links via Menshutkin reaction, (b) thermally activated reaction of styrenic groups during membrane casting, and (c) cross-linking by a blending approach using a bromoalkylated SEBS.

Introducing covalent cross-links in AEMs can efficiently reduce water uptake and swelling, improve mechanical properties, and increase the ionic content. Cross-linked poly(arylene piperidinium)s have usually been prepared via Menshutkin reactions between *N*-methylpiperidine groups and different α,ω-dibromoalkanes. The two components are typically cast together and the reaction takes place as the AEM is being formed. That usually means that the rate of the cross-linking reaction has to be carefully tuned with the rate of solvent evaporation in order to obtain good AEMs. Employing this scheme, AEMs have been cross-linked using hydrophobic,^249,250^ hydrophilic,^250^ and various dicationic^251−255^ α,ω-dibromoalkanes. The latter adds further cyclic or noncyclic quaternary ammonium cations in the AEM, and may increase the IEC while decreasing the water uptake which often favors the ionic conductivity. For example, Lammertink et al. introduced cross-links by quaternization using a α,ω-dibromoalkane containing a pair of piperidinium cations (Figure 22a), and found that the conductivity (up to 95 mS cm^–1^ at 80 °C) and dimensional stability increased simultaneously.^251^ In addition, a water electrolyzer cell with these AEMs exhibited a current density of 880 mA cm^–2^ at 2.2 V in 1 M KOH at 80 °C (Ni-based catalysts). During a durability test, the voltage remained almost stable at 1.81 V during 500 min at a current density of 100 mA cm^–2^ at 50 °C.^251^

Cross-linking has also been achieved using thermally initiated radical mechanisms. Lee et al. quaternized poly(*p*-terphenyl *N*-methylpiperidine) with 4-vinylbenzyl chloride to introduce styrenic groups that were then used to cross-link the AEM thermally during casting (Figure 22b).^256^ The AEMs combined high ion conductivity (>150 mS cm^–1^) and tensile strength (>80 MPa). Mayadevi et al. found that a terphenylene based polymer similar to structure **a** in Figure 19, but with a 50% molar ratio of *m*/*p*-terphenylene, had an optimized conductivity and tensile strength.^197^ Song et al. scaled up the synthesis of this polymer into the kg-level and, by applying the described cross-linking method with vinylbenzyl chloride, prepared membranes of 1 m width in a roll-to-roll process.^257^ The membrane was tested in a flow battery, an AEM fuel cell and an AEM WE, where a current density of 5.4 A cm^–2^ at 1.8 V at 90 °C was achieved. At 60 °C and 0.5 A cm^–2^, with 1 M KOH feed, the cell showed a stable performance with a voltage decay rate of just 15 μV h^–1^ over a time of 2,500 h. When the operating conditions were changed over the following 500 h to 1.0 A cm^–2^ at 80 °C, the voltage decay rate increased to 130 μV h^–1^.

Using a similar cross-linking strategy, Zhao and co-workers instead quaternized with 6-bromo-1-hexene and utilized thiol-ene click chemistry to form the cross-links.^258^ Finally, Kim et al. have introduced cross-links by quaternizing the *N*-methylpiperidine groups with surface-modified polyhedral oligomeric silsesquioxane nanoparticles to prepare composite membranes.^259^ They found that the hydration properties and the chemical stability improved significantly after cross-linking using the nanoparticles. Moreover, a water electrolyzer cell assembled with a composite AEM showed an overpotential of 1.72 V at 700 mA cm^–2^ when fed with 1 M KOH solution at 50 °C, which was significantly lower than for a corresponding cell with a commercial FAA-3-PK-140 membrane (2.68 V).

There are several reports on cross-linking achieved in a blending approach using polymers functionalized with benzylhalide or alkyl halide groups that react via Menshutkin reactions during membrane casting. Examples of these polyfunctional cross-linking polymers include poly(vinylbenzyl chloride),^260^ benzylbrominated poly(phenylene oxide)^261^ and bromohexylated^262−264^ and chloromethylated^265^ styrene-(ethylene-*co*-butylene)-styrene (SEBS) triblock copolymers. The latter is a type of thermoplastic elastomers that may introduce a separate rubber phase in the AEM to significantly enhance mechanical properties. For example, Kim et al. cross-linked poly(1,2-diphenylethane piperidinium) using a bromohexylated SEBS (Figure 22c) and found excellent mechanical properties at 40% cross-linking (including 70% elongation at break and a Youngs modulus of 486 MPa) in combination with a high hydroxide conductivity (up to 146 mS cm^–1^ at 80 °C) and stability (99% of the conductivity retained after 720 h in 2 M KOH at 80 °C).^264^ An electrolyzer cell operating with the membrane reached a current density of 1042 mA cm^–2^ at 1.8 V, which was 178% of the value obtained with a commercial FAA-3–50 AEM in the same study.

Hence, the results obtained by cross-linking demonstrate that this is a versatile and efficient strategy to tune and improve AEM properties. Perhaps most importantly, cross-linking can be employed to restrict membrane water uptake and swelling, and to improve mechanical properties by, e.g., increasing the modulus (stiffness) and reducing creep. Moreover, the IEC of the AEM can be increased and controlled by using ionic cross-links.

#### 3.4.2 Membranes Based on Poly(Arylene Alkylenes) Functionalized with Quaternary Ammonium Cations

An attractive strategy to prepare durable AEM polymers by hydroxyalkylation is to employ functional arene or carbonyl monomers that carry short bromoalkyl chains, or alternatively aminoalkyl chains, which can take part in Menshutkin reactions to introduce different quaternary ammonium cations. These functional monomers include trifluoromethyl bromoalkyl ketones,^139,169,266−273^ dibromoalkylated fluorenes,^274−283^ bromoalkylated carbazole^284,285^ and bromoalkylated isatin.^286−296^ The strategy follows the initial report on poly(arylene alkylene)s (Figure 14) for AEMs by Bae and co-workers.^169^ They synthesized a trifluoromethyl bromopentyl ketone and then polymerized it with biphenyl to obtain high-molar mass poly(biphenyl alkylene), before quaternizing the pendent bromopentyl chains with trimethylamine to obtain the final TMA-functional polymers. Their AEMs reached hydroxide conductivities of 120 mS cm^–1^ at 80 °C and showed excellent alkaline stability.^169^ Bae et al. continued to study the effect of different arene monomers, including biphenyl, *p*-terphenyl, and *m*-terphenyl, and found that the use of the latter monomer gave a more ordered morphology and a higher conductivity, possibly due to the higher chain flexibility of the corresponding polymer backbone.^139^ Using membrane electrode assemblies based on the same ionomers as in the respective AEM, water electrolyzer cells with these AEMs reached a current density of 400 mA cm^–2^ at 2.1 V and 50 °C, and displayed a good stabilization of the cell voltage during 360 min at 200 mA cm^–2^, although needing mechanical stabilization.^266^ These AEMs later formed the basis for the reinforced AEMs marketed by Orion polymers. Bae et al. have also evaluated these AEMs in fuel cells^267^ and vanadium flow batteries.^268^

**Figure 23:**
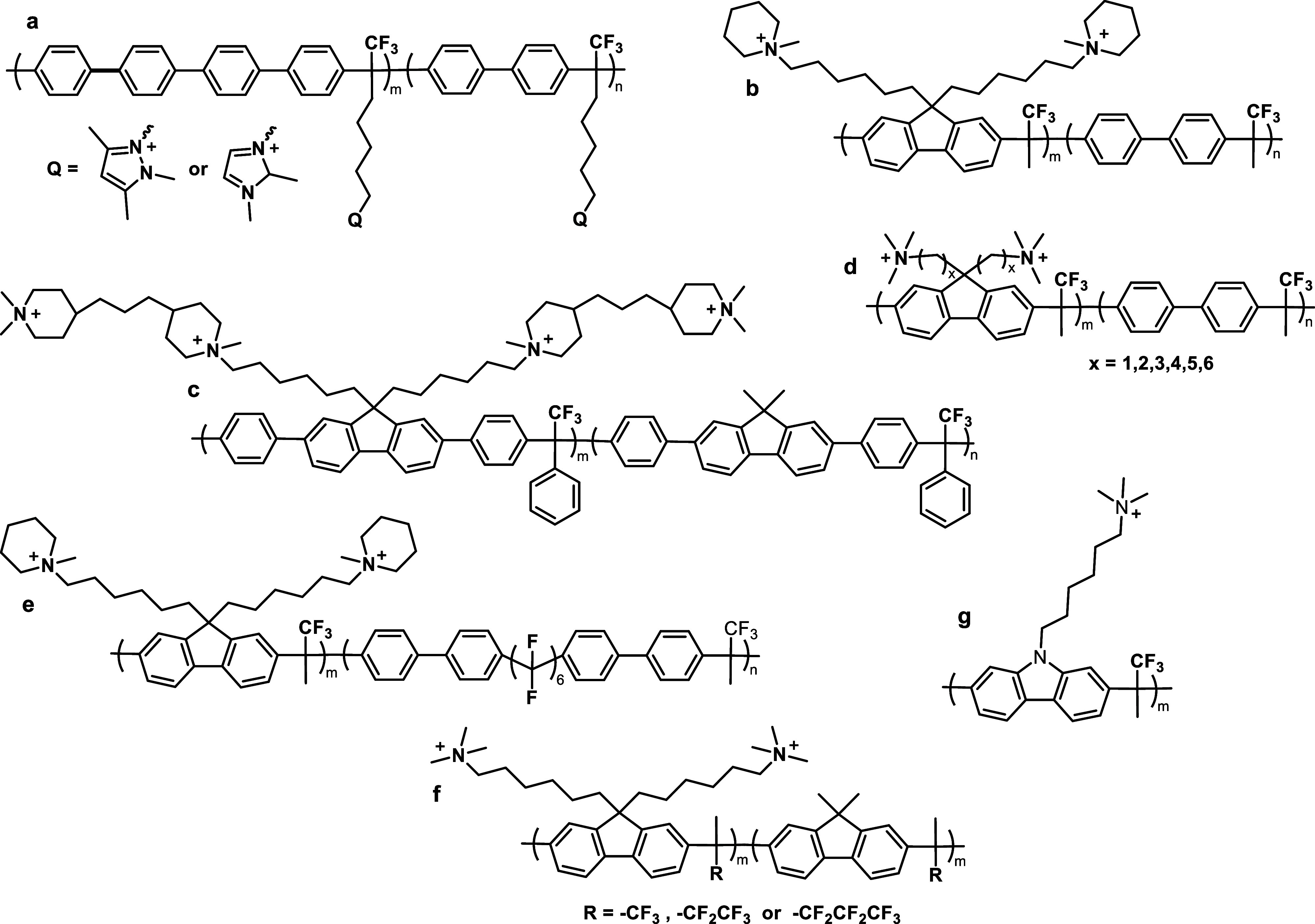
Poly(arylene alkylene)s with backbones based on (a) bi- and quaterphenyl, (b–f) fluorene, and (g) carbazole, all tethered with QA cations via flexible alkyl chains.

Wei, Ding, and co-workers have further studied poly(arylene alkylene) AEMs derived from the trifluoromethyl bromopentyl ketone monomer, and have investigated the influence of using quaterphenyl as comonomer,^269^ introducing dicationic cross-links,^270^ and branching,^271^ respectively. In addition, they have considered the influence of the alkyl chain length (*n* = 4, 5, 6) on the AEM properties and discovered that increasing length significantly improved the microphase separation and conductivity (up to 180 mS cm^−1^ at 80 °C), as well as alkaline stability, while the swelling decreased.^272^ Wei and Ding et al. also functionalized their polymers with pyrazolium cations as alternative ion exchange groups (Figure 23a) and reported higher alkaline stability than for imidazolium cations, which was rationalized by density functional theory.^273^

##### 3.4.2.1 Fluorene- and Carbazole-Based Poly(Arylene Alkylene)s

Fluorene is a tricyclic semiaromatic arene with weakly acidic protons in the C9-position. Deprotonation using for example aqueous NaOH produces the nucleophilic and aromatic fluorenyl anion, which has an intense orange color. This provides the opportunity to consecutively deprotonate and add two bromoalkyl chains to the C9-position. Hence, the use of an excess of an α,ω-dibromoalkane will result in a dibromoalkylated fluorene monomer that, before or after the polyhydroxyalkylation, can be used to introduce a pair of quaternary ammonium cations in a Menshutkin reaction. This enables an attractive and straightforward pathway to place cations on flexible alkyl spacers within close proximity into the polymers structure, which enables a high local ionic concentration to facilitate ionic clustering and conductivity.^297,298^ In an early work, Varcoe, He, and co-workers prepared poly(fluorene biphenyl alkylene)s tethered with pairs of piperidinium cations on C6 spacers (Figure 23b) and recorded a conductivity of 86 mS cm^–1^ at 80 °C, with no detectable degradation after 1200 h at 80 °C in 1 M NaOH.^274^ Jannasch and co-workers later prepared 2,7-diphenylated fluorene monomers to increase the reactivity in the polyhydroxyalkylation reaction, and found that poly(diphenylfluorene alkylene)s carrying double pairs of bispiperidinium cations reached up to 150 mS cm^–1^ at 80 °C (Figure 23c).^275^ Furthermore, it was shown that a limited ionic loss under harsh conditions (5 M NaOH, 90 °C) was mainly due to β-elimination in the spacer chains, and not in the piperidinium rings. The same group has also prepared and studied poly(diphenylfluorene alkylene)s functionalized with 1,1-dimethylpiperidinium^276^ and cage-like quinuclidinium^277^ cations via alkyl spacers. They reported exceptionally high alkaline stabilities for these very stable cations. For example, no ionic loss was found after 168 h at 90 °C in 5 M NaOH in the former case.

Li and Zheng et al. used different synthetic routes to obtain dibromoalkylated fluorenes with various lengths of the alkyl spacers (1–6 carbons) (Figure 23d).^278^ They found that the length of the spacer chain had a profound influence on the morphology and properties of the AEM based on poly(fluorene biphenyl alkylene)s. Hence, the alkaline stability and hydroxide conductivity increased concurrently with a decrease of the water uptake. The AEM carrying TMA cations on C6 alkyl spacers reached a conductivity of 154 mS cm^–1^ at 80 °C with no degradation observed after 30 days in 2 M NaOH at 80 °C.^278^ Later, the same group also prepared and studied polyfluorenes tethered with flexible alkyl chains carrying 1, 2, or 3 cations, respectively, and reached 203 mS cm^–1^ at 80 °C with a blocky monomer distribution leading to very high local ionic concentrations in the AEM.^279^ Several research groups, including those of Miyatake^280^ (Figure 23e), Kim and Park^281^ (Figure 23f), and Lee and Klok,^282^ have introduced perfluoroalkyl segments in polyfluorenes in order to control the water uptake and improve the microphase separation to enhance the hydroxide conductivity. Also, Xu et al. have utlized bromoalkyl chains tethered to the fluorene units of poly(fluorene *p*-terphenyl alkylene) in a Menshutkin cross-linking reaction to simultaneously introduce quaternary ammonium cations and hydrogen-bonding urea units.^283^

Carbazole is a tricyclic arene compound with an acidic proton in the N9-position, and is thus related to fluorene. Similar to fluorene, this heterocyclic compound can be deprotonated by KOH to form the nucleophilic carbazolate anion which can react with, e.g., dibromoalkanes to produce bromoalkylated carbazole monomers.^284,285^ This has been exploited by Lee et al., who polymerized *N*-bromohexylcarbazole with a trifluoromethyl ketone, and then quaternized to obtain terminal TMA cations (Figure 23g).^284^ The AEMs showed distinct microphase separation and high conductivity (154 mS cm^–1^ at 80 °C). Evaluations in water electrolysis cells at 70 °C showed that the carbazole AEMs produced a very high current density, 3.5 A cm^–2^ at 1.9 V.^284^

In conclusion, fluorene- and carbazole-based monomers are quite reactive in polyhydroxyalkylations and can be readily modified, either before or after the polymerizations, to carry ionic groups on alkyl spacers. Alternatively, other functional groups can be introduced, including hydrophobic segments to enhance phase separation and reduce water uptake, or reactive groups for cross-linking, as discussed above.

##### 3.4.2.2 Isatin-Based Poly(Arylene Alkylene)s

**Figure 24:**
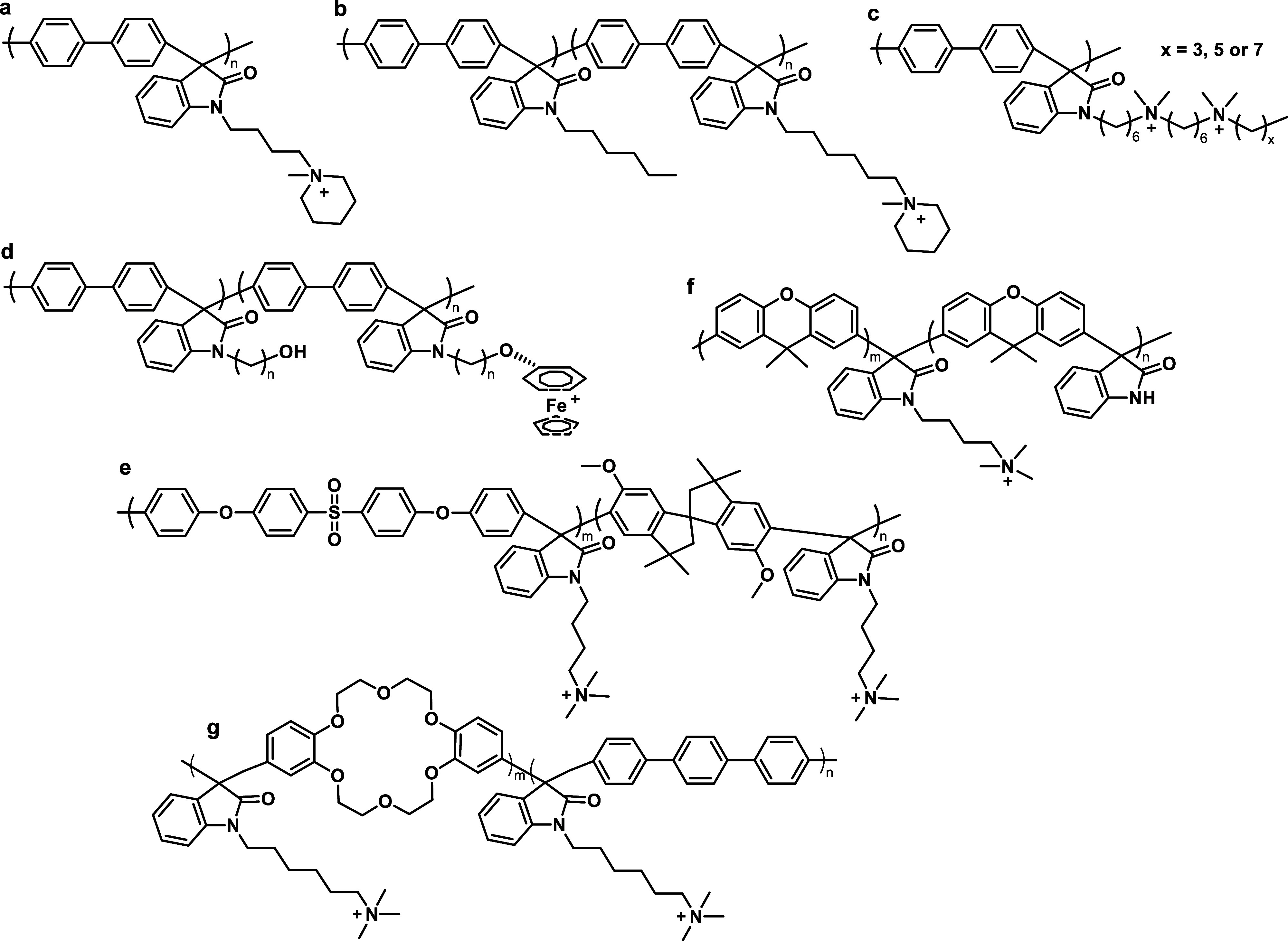
Poly(arylene oxindole)s tethered with QA cations via flexible alkyl chains.

Isatin is an accessible weakly acidic heterocyclic compound that can be conveniently bromoalkylated and utilized as a highly reactive ketone monomer in polyhydroxyalkylations to produce AEMs. Alternatively, alkylation can be performed on the oxindole units formed by isatin during the polymerization to introduce suitable functionalities or cations. Varcoe, He, and co-workers polymerized bromoalkylated isatin and biphenyl, followed by quaternization to obtain pendent piperidinium cations (Figure 24a).^286^ By extending the alkyl chain length from 1 to 5 carbon atoms, they found that both the microphase domain size and the conductivity increased, to reach 74 mS cm^–1^ at 80 °C. In addition, longer alkyl chains resulted in reduced water uptake and improved alkaline stability,^286^ which agrees well with the results obtained with fluorenes and trifluoromethyl ketones discussed above. Other investigations of AEMs based on bromoalkylated isatines have focused on the influence of hydrophobic hexane side chains^287^ (Figure 24b), different *N*-alicyclic cations^288^ (pyrrolidinium, piperidinium), various pendent dicationic alkyl chains^289,290^ (Figure 24c), as well as cross-linking with dicationic alkyl chains.^291^ In addition, poly(biphenyl oxindole) has been hydroxyalkylated and then functionalized with ferrocenium cations (Figure 24d).^292^ However, AEMs based on these polymers showed limited alkaline stability with almost 20% conductivity loss over 1,000 h in 1 M KOH at 60 °C.

Several research groups have incorporated rigid bulky units, like cyclodextrin,^293^ or contorted backbone structures with fused rings, such as spirobiindane^294^ (Figure 24e) and xanthene^295^ (Figure 24f), respectively, into isatin-based polymers with the aim to induce microporosity in the AEMs for increased conductivity. For example, Zhang and co-workers prepared a polymer from isatin and 9,9-dimethylxanthene, which was then bromoalkylated to introduce TMA cations (Figure 24f).^295^ The AEMs displayed both microporosity and microphase separation and reached a conductivity of 205 mS cm^–1^ at 80 °C with a degree of swelling of less than 15%. Similarly, He et al. tethered poly(isatin terphenyl) containing hydrophilic crown ether (18-crown-6) with TMA-terminated hexyl chains, and reported AEMs with interconnected ion conducting channels leading to a conductivity of 112 mS cm^–1^ at 80 °C (Figure 24g).^296^

##### 3.4.2.3 Special Monomers and Polymerizations

**Figure 25:**
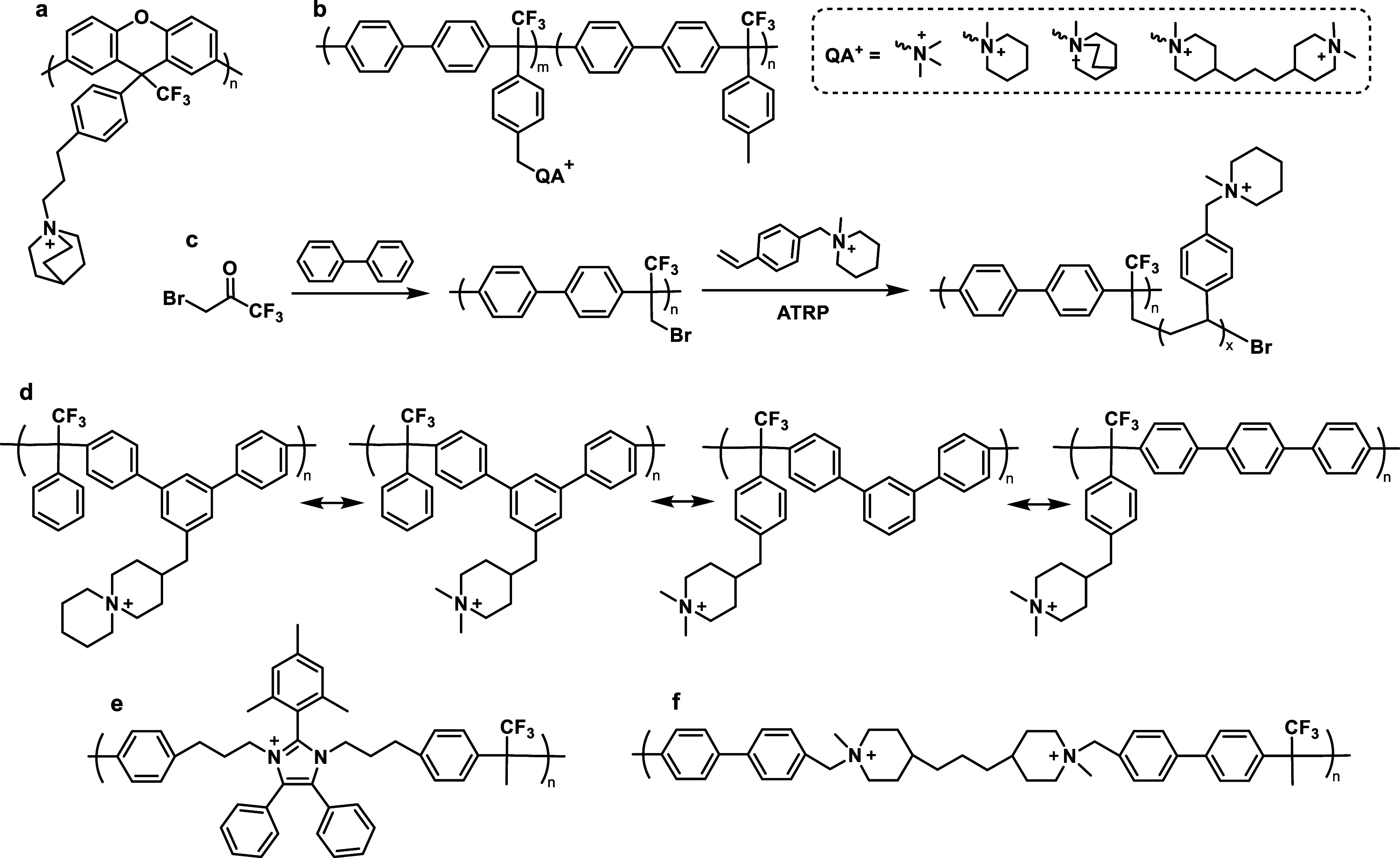
Poly(arylene alkylene)s with (a) xanthene backbones, (b) benzylic QA cations, (c) cationic side chains prepared by ATRP, (d) different backbone configuration and QA placement, and (e) protected imidazolium cations in the backbone (ionene), and (f) bis-piperidinum cations in the backbone (ionene).

Arene and ketone monomers functionalized with bromoalkyl and amine groups, respectively, ready for use in quaternization reactions have also been prepared in alternative routes in order to simplify synthesis or to study new AEM properties. For example, Narducci et al. used lithiation chemistry to prepare a bromoalkylated biphenyl monomer^299^ (Figure 16s), and Jannasch et al. prepared bromoalkylated 2,2,2-trifluoroacetophenone (Figure 17f) in a one-step Friedel–Crafts reaction and used the monomer in a polyhydroxyalkylation with 4,4′-biphenol to produce poly(xanthene)s (Figure 25a).^300^ A xanthene AEM tethered with quinuclidinium cations had a conductivity above 100 mS cm^–1^ at 80 °C, and displayed high alkaline stability with no structural change or ionic loss detected by NMR analysis after 720 h in 2 M NaOH at 90 °C. In another approach, a polymer was prepared based on 4-methyl-2,2,2-trifluoroacetophenone and biphenyl or *p*-terphenyl, after which some of the methyl groups were transformed into bromomethyl groups (Figure 25b).^301^ After quaternization, the AEMs reached conductivities of 133 mS cm^–1^ but had limited alkaline stability because of nucleophilic attack on the benzylic cations. Following a different synthetic strategy, Zhu et al. used 3-bromo-1,1,1-trifluoroacetone in a polyhydroxyalkylation with biphenyl where the resulting bromoalkyl groups along the polymer backbone were utilized as initiator sites for atom transfer radical polymerization (ATRP) of styrenic piperidinium cations (Figure 25c).^302^ The resulting comb-shaped copolymers had a phase separated microstructure, and, with cross-linked particles added, AEMs reached a chloride conductivity of 65 mS cm^–1^ at 60 °C. In general, ATRP brings rich possibilities to prepare graft copolymers to employ “phase engineering” to tailor AEM morphology and properties.^303^

With the primary aim to investigate the influence of backbone flexibility and cation placement on the AEM stability, Jannasch et al. functionalized both *m*-terphenyl and 2,2,2-trifluoroacetophenone with *N*,*N*-dimethylpiperidinium cations (Figure 25d).^304^ The study indicated increased stability with increased flexibility of the backbone and increased local mobility of the cation. The best-performing AEM displayed a conductivity of 146 mS cm^–1^ at 80 °C, with less than 5% ionic loss after 720 h in 2 M NaOH at 90 °C.^304^ Zhang and co-workers prepared a diphenylalkylated protected imidazolium monomer (Figure 16x), similar to the cations found in the commercial AEMION-type AEMs, and copolymerized it with biphenyl and 1,1,1-trifluoroacetone to obtain linear high molar mass poly(arylene imidazolium) ionene AEMs (Figure 25e).^305^ Although the AEM displayed a well-defined phase separation, the conductivity only reached a moderate 70 mS cm^–1^ at 80 °C, which was most probably due to the limited IECs (1.17–1.42 mequiv. g^–1^). On the other hand, the AEM showed no apparent degradation after immersion in 10 M NaOH at 80 °C during 2,400 h, indicating an excellent stability of the protected imidazolium ionenes.^305^ Using a similar approach, Yang et al. have prepared and polymerized a diphenyl-functionalized bis-piperidinum monomer with 1,1,1-trifluoroacetone to produce a poly(arylene piperidinium) ionene (Figure 25f).^306^ The corresponding AEM had an IEC of 2.25 mequiv. g^–1^ and reached a hydroxide conductivity of 45 mS cm^–1^ at 80 °C and 26% water uptake. No degradation was detected by NMR analysis after storage during 480 h in 1 M KOH at 80 °C.

In conclusion, there are ample opportunities to molecularly design and synthesize new monomers to enable the preparation of polymers that incorporate alkali-stable cations either directly in the backbone or placed on side chains. Synthetic strategies by which monomers are synthesized by functionalizing an ionic group or segment with two aromatic groups, such as phenyl or biphenyl, are especially versatile and straightforward in the synthesis of ionenes, as seen in Figure 25e and f.

### 3.5 Poly(Styrene)-Based Membranes

**Figure 26:**
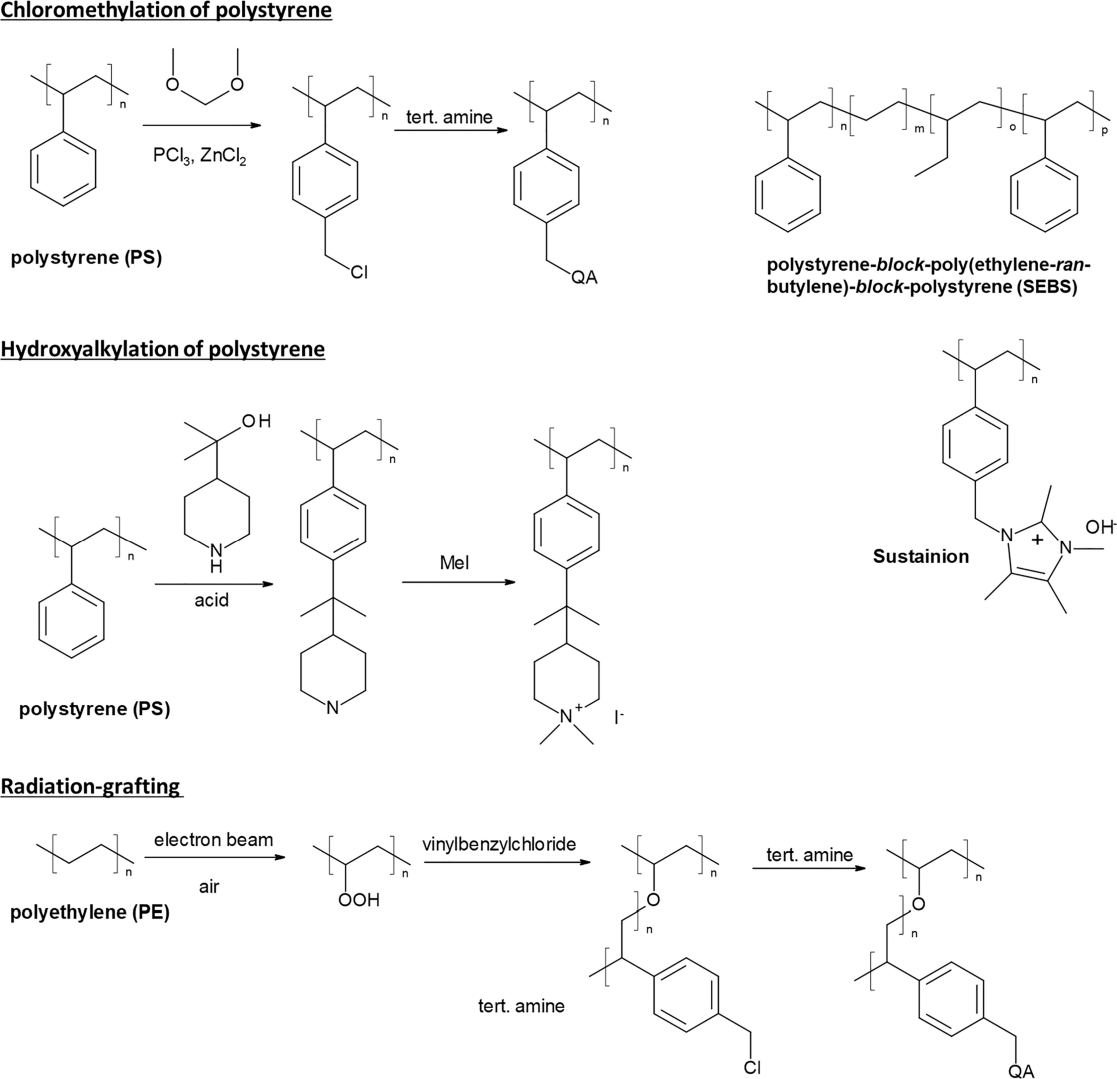
Styrene-based polymers and synthesis of AEMs. QA: Quaternary ammonium.

Polystyrene is a cheap commodity polymer. It has only carbon atoms in the backbone, which should result in high alkaline stability, and the phenyl rings can be selectively chloromethylated in the 4 position and subsequently functionalized by reaction with a tertiary amine or imidazole.^132−134,307^ The same chloromethylated polymers can be obtained also by direct polymerization of 4-vinylbenzyl chloride.^308,309^ Another convenient approach is hydroxyalkylation or in general a Friedel–Crafts alkylation of the 4 position.^310−312^ Chemical structures and synthesis paths are shown in Figure 26. The drawback of polystyrene is its intrinsic brittleness. To improve the mechanical properties, polystyrene can be radiation grafted on aliphatic vinyl polymers like ETFE^313^ or PE,^314,315^ or it can be copolymerized. Examples for block copolymers are SES^316^ or SEBS,^133,307^ which are commercially available, rubbery materials.

A prominent commercial membrane based on polystyrene is Sustainion (Table 3, #1–#4).^131,132^ These membranes are produced by copolymerization of styrene, vinylbenzyl chloride and divinylbenzene, followed by reaction with 1,2,4,5-tetramethylimidazole (TMI). As shown by Hugar et al., TMI-based imidazolium ions have a very high alkaline stability.^152^ Swelling of Sustainon membranes is controlled by cross-linking the polystyrene backbone with divinylbenzene and allowing water traces to be present in the system; according to Rich Masel, these traces compete with TMI and consecutively react with one chloromethyl group to a benzyl alcohol, which forms an ether with a second chloromethyl group. The intrinsic brittleness of polystyrene is tackled by reinforcing it with a porous support and/or doping the membrane with ethylene glycol. This high boiling point solvent plasticizes the membrane and thus facilitates handling. Before use, the producer advises to remove the plasticizer by immersion in 1 M KOH solution overnight. When the membrane is assembled immediately in the wet state, it does not form cracks.

**Table 3:** Properties of Styrene-Based Membranes and Electrolyzer Performances

#	membrane	IEC (mmol/g)	conductivity (mS/cm)	cell performance	ASR (Ω cm^2^)	reference
1	Sustainion 37–50	1.1	72 (1 M KOH, 25 °C)	60 °C, 1 M KOH, NiFe_2_O_4_ anode, NiFeCo cathode:	0.045 (1 M KOH, 60 °C; cell)	Z. Liu et al. (2017)^319^
			116 (1 M KOH, 60 °C)	1 A/cm^2^: ca. 1.9 V, tested for 1950 h, average voltage increase 5 μV/h		
2	Sustainion 37–50 grade T	1.1	-	50 °C, 1 M KOH, ternary NiFeV	-	B. Motealleh et al. (2021)^131^
				layered double hydroxide anode, Pt/C cathode:		
				2.1 A/cm^2^: ca. 1.8 V, tested for 100 h		
3	Sustainion 37–50 grade T	1.1	-	60 °C, 1 M KOH, NiFe_2_O_4_ anode, Raney nickel cathode: 1 A/cm^2^: 1.85 V with a degradation rate 0.7 μV/h over 12,180 h	0.08 (1 M KOH, 60 °C; cell)	B. Motealleh et al. (2021)^131^
4	Sustainion 37–50 grade T	1.1	-	42–45 °C, 1 M KOH, Ni_0.75_Fe_2.25_O_4_ anode, Pt/C cathode: 1.9 V	-	J. Lee et al. (2021)^320^
				at 2.0 A cm^–2^, but ca. 200 mV increase over 21 h at 500 mA/cm^–2^		
5	SEBS-CH_2_-DABCO	0.76 (by UV/vis)	75 (OH form in water, 30 °C; pretreatment: 1 week in 10 wt % KOH)	50 °C, 10 wt % KOH, nickel foam as anode, cathode. 300 mA/cm^2^ → 2.27 V, 150 h test	1.87 (5 wt % KOH, 40 °C, cell)	J. Hnát et al. (2017)^133^
6	SEBS-CH_2_-DABCO	0.75	Water, OH form:	Operation up to 80 °C in 21 or 31 wt % KOH for several days showed no degradative effect on structural integrity, ASR and gas purity	217 μm thick membrane, cell:	J. Brauns et al. (2021)^94^ J. Žitka et al. (2019)^135^
			56 (30 °C)		21 wt % KOH:	
			75 (50 °C)		0.38 (50 °C)	
			79 (70 °C)		0.33 (60 °C)	
			21 wt % KOH:		31 wt % KOH:	
			45 (25 °C)		0.30 (50 °C)	
					0.26 (60 °C)	
			31 wt % KOH:			
			33 (25 °C)			
7	SEBS-CH_2_-*N*-methylpiperidinium	1.19 (titration)	Water, OH form: 10 (30 °C)	50 °C, 1 M KOH, IrO_2_ anode, Pt/C cathode, 400 mA/cm^2^ → 2.08 V, 105 h	0.33 (1 M KOH, 50 °C, at 1.8 V)	X. Su et al. (2020)^307^
		1.65 (theory)	14 (50 °C)			
			17 (60 °C)			
8	cross-linked SEBS-CH_2_-TMA/SEBS-CH_2_-*N*-alkyl piperidinium	1.06	20.8 in 1 M KOH	60 °C, 0.1 M KOH, Ir anode, Pt/C cathode, 2 V → 680 mA/cm^2^, degradation rate ≈1 mA/cm^2^ h^–1^	n/a	Z. Xu et al. (2023)^321^
9	radiation grafted PE-*g*-VBC, functionalized with TMA	2.3	OH form, 100% relative humidity:	60 °C, 0.1 M NaOH, NiCo_2_O_4_ catalyst, 1 A/cm^2^ → 1.97 V (from polarization curve; strong degradation was observed, presumably due to loss of catalyst particles)	0.13 (50 °C, 100% relative humidity, membrane)	G. Gupta et al. (2018)^315^
			90 (50 °C)			

SEBS copolymers have a hydrophobic poly(ethylene-*co*-butadiene) block capped on both ends with a polystyrene block. After ionic functionalization of the polystyrene units, SEBS easily phase separates into hydrophobic and hydrophilic domains, and water interacting with the hydrophilic groups results in strong plasticization. One TMA-modified SEBS membrane had a tensile strength of 14 MPa at 50 °C under dry conditions, but just 1 MPa at 90% relative humidity.^317^ Presumably, contact with liquid water will plasticize the membrane even more, and it appears that cross-linking is inevitable. Similar to Sustainion, such a cross-linking could be by reacting the chloromethylated SEBS with trace amounts of water; but also strong drying of the chloromethylated form may result in cross-linking, either mechanically by chain entanglement or chemically when chloromethyl groups react with phenyl groups under formation of a methylene bridge. Another reactive site is the C–C double bond present when butadiene is polymerized; hydrogenation removes the C=C double bond (one needs to check the exact nature of commercial SEBS), and the resulting more regular structure increases the degree of crystallization of the hydrophobic phase and thus enhances the mechanical strength of SEBS-based AEMs.^318^ Recently, DABCO-modified SEBS membranes similar to #5 and #6 in Table 3 became commercially available under the trade name Hollex through the Czech company Tailormem.

### 3.6 Reinforcement Strategies to Strengthen AEM

By increasing the density of ionic groups (i.e., the ion exchange capacity), high conductivities can be reached. The drawback is that high IEC values also result in high water uptake, and thus large swelling and softening of the membranes. Porous support materials can increase the tensile strength and Young’s modulus of membranes and thus ease handling (which can be an issue when moving to large industrial-scale areas), resistance against differential pressure, and prevent dimensional changes.

In fuel cells, changes in the relative humidity of the gas streams reversibly swells and shrinks the membranes, resulting in detrimental mechanical stresses. In electrolyzers, changes from dry to wet states should ideally only occur during the initial start-up, if membranes were assembled in the dry state and are contacted for the first time with the feed solution. For lab based single electrolysis cells, this effect can be addressed by assembling wet membranes. For large areas or stacks, preswelling with a high-boiling point solvent like ethylene glycol can efficiently minimize the dimensional changes during assembly.^322^

Dimensional changes of wet AEMs are also expected, when the temperature alternatingly increases and decreases. Especially at lower temperatures, e.g., in the range of room temperature to 40 °C, many membranes show a spring-like behavior and noticeably swell and shrink according to changes in the temperature.^128^ However, above a certain temperature, when the AEM is strongly swollen, the response to decreasing temperatures slows down, and for some membranes it may take several weeks to get back close to the original dimension. Therefore, it appears that swelling and shrinking can be prevented if membranes are assembled in a preswollen state, always kept in contact with water, and if the operating temperature is above the point at which the membrane loses its spring-like properties. Unfortunately, at this point, membranes are strongly plasticized and lose their mechanical strength. For example, a 20 μm thick Versogen membrane showed tensile strength values of 25, 12, and 9 MPa in the dry state, in water at 30 °C, and in water at 60 °C, respectively, a Young’s modulus of 657, 240, and 85 MPa, and an elongation at break of 14%, 17%, and 23%.^128^ To strengthen the soft membranes, practically all membrane producers offer or develop membranes with a porous reinforcement.

Commonly used reinforcements are made of PTFE or hydrocarbons like PEEK or polyolefins, and morphologically are fabrics, nonwoven or stretched films. As mentioned, hydrolysis is not expected to play a major role, because hydroxide ions cannot enter into the hydrophobic materials. However, materials like PVDF, which react rapidly with hydroxides under formation of hydrophilic groups, may degrade rapidly.^97^

Engineering challenges are the pore filling process and the formation of a stable interface. While hydrophobic support materials are expected to show a long lifetime in aqueous alkaline conditions, it can be difficult to wet their pores with viscous, polar polymer solutions. This can be tackled by prewetting the surface of porous support films with solvents or tensides,^323,324^ by chemical etching (for example contacting porous Teflon with a sodium-naphthalene solution for 5 s,^325^) or plasma treatments.^326^ Once the pores are filled with the polymer solution, it has to be ensured that evaporating solvent does not result in pores. This can be addressed by embedding the porous support inside of a freshly casted polymer solution film, or by casting first solution on one side, and after drying, on the other. However, thick porous supports may still show some unfilled volumes. The other challenge is to maintain the matrix|support interface. It is known from the fuel cell field that repeated swelling and shrinking delaminate the matrix and the support, and the voids are pathways for increased gas crossover.^325^ A similar mechanism is expected for reinforced AEM. Indeed, the water vapor permeation through some reinforced AEMs was higher than through the nonreinforced membrane.^128^

An elegant way to solve the issue in the PEM fuel cell field is the use of PBI nanofiber mats as a reinforcement for Nafion, which results in a stable interface due to ionic interactions between the sulfonic acid groups and the imidazole groups.^327^ For AEM, PBI nanofiber mats offer an additional advantage. Many AEMs can be cast from haloalkylated precursor polymers, which are quaternized by immersion in an amine solution. When such precursor polymers are pore filled into PBI fiber mats, they can react with the PBI imidazole groups on the fiber surface, forming covalent bonds between matrix and support (Table 4, #5).^121^ In another work, Abouzari-Lotf et al. prepared electrospun nanofibermats of PP and Nylon, irradiated them, and established covalent bonds by filling the pores with a vinylbenzylchoride solution, which then started to grow polymer chains from the irradiated nanofiber surfaces. Consecutively, some of the units were cross-linked by 1,6-diaminooctane, the remaining units were quaternized by either TMA or 1,2-dimethylimidazole, and the still porous membranes were densified by compression between ETFE sheets in a hot press (Table 4, #6, #7).^328^

**Table 4:** Reinforced Membranes and Their Properties^a^

#	membrane type	support material	thickness (μm)	conductivity (Cl^–^ form, mS cm^–1^)	conductivity (OH^–^ form, mS cm^–1^)	reference
1	Sustainion	PTFE	ca. 50	30 (1 M KCl, 30 °C)	80 (1 M KOH, 30 °C)	dioxide materials
	X37-50 grade T					
2	Fumasep	polyketone mesh	75	4.5–6.5	49 (30 °C)	Fumatech, H. Khalid et al. (2022)^128^
	FAA3-PK-75				121(60 °C)	
3	AEMION+	woven polyolefin	85 ± 9	6–7 (RT)	na	Ionomr, M. Moreno-González (2023)^151^
	AF2-HWP8-75-X					
4	PiperION	ePTFE	15	na	77 (30 °C)	Versogen, H. Khalid et al. (2022)^128^
	PI-15				194 (60 °C)	
5	PBI/mTPN (PBI-reinforced Orion Polymer TM1)	Electrospun PBI nanofibermat	ca. 50	-	73 (30 °C)	M. Najibah et al. (2021),^121^ H. Khalid et al. (2022)^128^
					151 (60 °C)	
6	1,6-diaminooctane cross-linked polyvinylbenzylchloride, EB-grafted on the surface of nanofiber mats, quaternized with either TMA or 1,2-dimethylimidazole	electrospun Nylon (PA-66)	14 (Im)	-	Im:	E. Abouzari-Lotf et al. (2021)^328^
			15 (TMA)		40 (30 °C)	
					130 (80 °C)	
					TMA	
					47 (30 °C)	
					126 (80 °C)	
7	same	electrospun *syn*-PP	17 (Im)	-	Im:	E. Abouzari-Lotf et al. (2021)^328^
			15 (TMA)		47 (30 °C)	
					149 (80 °C)	
					TMA	
					41 (30 °C)	
					133 (80 °C)	
**8**	PE-reinforced poly(fluorenyl-co-terphenyl piperidinium	polyethylene	16	-	32 (30 °C)	H.H. Wang et al. (2022)^195^
**9**	PE-reinforced poly(-diphenylethyl-co-terphenyl piperidinium)	polyethylene	21	-	77 (80 °C)	C. Hu et al. (2022)^194^
**10**	polyphenylene-based arylimidazolium	polyethylene	45	-	43 (RT)	A. M. Ahmed Mahmoud et al. (2022)^329^
					84 (80 °C)	
**11**	aliphatic dimethyl pyrrolinidium-based AEM cross-linked with piperazinium	PTFE	27	39 (30 °C)	134 (30 °C)	Z. Wang et al. (2020)^330^
				80 (70 °C)	288 (70 °C)	

a Information is taken from specification sheets and/or provided references.

From the various reinforcement materials shown in Table 4, PTFE has the highest chemical resistance, but raises issues for catalyst recycling, because burning the degraded MEA to concentrate the metals would release not only CO_2_ and water, but also HF.^18^ For this reason, polyaromatics and polyolefins are preferred and potentially also cheaper. Some materials are available as woven fabrics with well-defined mesh dimensions. The advantage is the high strength of the usually rather thick reinforcing fibers and the large space between the threads, which can be easily filled with the ion conducting matrix. On the other hand, the high rigidity of thick fibers could potentially enhance delamination, and the crossing points, at which two fibers overlap, are thick and may reach the membrane surface.

Nonwoven materials made by electrospinning result in very narrow fiber diameters and therefore show a high surface/fiber volume ratio, and individual fibers may have some mobility, which should prevent rapid delamination. Depending on the process parameters, many electrospun nanofiber mats lack connection points between the fibers, and have a fluffy nature. Post-treatments like solvent-welding or calendaring can increase the fiber–fiber interactions and thus the overall robustness of the fibermat.^331^ From a practical perspective, it appears that electrospinning is a slow and thus costly process for mass production.

Widely available materials are porous polyolefins like porous PE or PP, which are mass produced as separator for battery applications. Typically, PE is produced by casting a polymer/solvent mixture, thermal phase separation by reducing the temperature after casting, followed by biaxial stretching of the film to the desired pore structure, and removal of the solvent. PP films are typically dry-processed by melt-extrusion followed by biaxial stretching.^332^

An equation which describes capillary phenomena and thus is closely related with the efficiency of pore filling processes is the Lucas-Washburn equation (eq 2).^307,333,334^
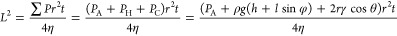
2

In this equation, *L* is the penetration length, *P*_A_ is the atmospheric pressure, *P*_H_ is the hydrostatic pressure from the liquid on the pore, and *P*_C_ is the pressure of the capillary force. *L*^2^ is proportional to the sum of the different pressure contributions (∑ *P*), square of the pore radius (*r*), and the time (*t*). The capillary force parameter is related to the mean pore radius (*r*), viscosity (η) of the solution, and the surface tension (γ). Based on the Lucas-Washburn equation, pore filling is more efficient (i.e., faster or more complete for a given process time) when the polymer solution has a low viscosity (low polymer concentration) and low surface tension, and for porous supports with large pore diameter.^195^ A high porosity is also wanted to minimize the losses in conductivity. Higher polymer concentrations of the casting solution are preferred to prevent defects when the solvent evaporates. Presumably, unwanted voids and waviness can be prevented if the porous substrate yields (shrinks) in thickness direction during solvent evaporation but is rigid in the area.

In summary, while delamination of porous supports and the ion-conducting matrix can increase gas crossover, porous supports help to reduce swelling/shrinking, creep, increase the mechanical strength. The latter point not only increases lifetime, but also can allow to manufacture thinner membranes, to optimize membrane resistance. While many works report progress on new AEM materials, work on reinforced composite membranes is comparatively scarce, but necessary to optimize membranes toward large-scale commercialization. Many targets need to be reached: (1) A high porosity and (2) large pore diameter for efficient pore filling and low resistance, (3) a cheap production process, (4) good wettability for efficient pore filling, and (5) formation of a strong, preferentially covalent matrix|support interface to prevent delamination during cell operation.

## 4 Key Performance Indicators and How to Assess them

### 4.1 Key Performance Indicators

A recent review by Chatenet et al. provides information on state-of-the art alkaline and AEM electrolysis systems and target values for 2050.^335^ Those properties which are partially or fully related to the diaphragm or membrane are shown in Table 5. It is noteworthy that several targets merge for the two systems. Regardless if an alkaline or an AEM electrolysis system is used in 2050, the nominal current density should be >2 A cm^–2^, the cell pressure >70 bar, the H_2_ purity >99.9999%, the electrical efficiency should be <42 kWh/kgH_2_, and the lifetime should reach 100,000 h. Several values are closely related. In order to reach a high current density, the area specific resistance must be low, i.e., the interelectrode distance should be small which requires a relatively thin membrane with high conductivity. This conversely affects the gas crossover and thus the H_2_ purity. Furthermore, H_2_ crossover is the sum of diffusion, convection and electro-osmotic transport. Therefore, the crossover increases not only with the pressure, especially in the case of differential pressure, but increases also with the current density, because solvated hydroxide ions transport dissolved hydrogen to the anode.

**Table 5:** Target Values for Electrolyzers as Summarized in Ref (335)

	2022		2050	
system	alkaline	AEM	alkaline	AEM
nominal current density	0.2–0.8 A cm^–2^	0.2–2 A cm^–2^	>2A cm^–2^	
operating temperature	70–90 °C	40–60 °C	>90 °C	80 °C
cell pressure	<30 bar	<35 bar	>70 bar	
load range	15–100%	5–100%	5–300%	5–200%
H_2_ purity	99.9–99.9998%	99.9–99.9999%	>99.9999%	
electrical efficiency (stack)	47–66 kWh/kgH_2_	51.5–66 kWh/kgH_2_	<42 kWh/kgH_2_	
lifetime (stack)	60,000 h	>5000 h	100,000 h	
stack unit size	1 MW	2.5 kW	10 MW	2 MW
electrode area	10,000–30,000 cm^2^	<300 cm^2^	30,000 cm^2^	1,000 cm^2^

For membranes and diaphragms, some key properties are conductivity, area specific resistance, H_2_ permeability, H_2_ crossover, water transport, dimensional stability during assembly into the stack and operation, bubble point, mechanical properties (tensile strength, elongation at break), alkaline stability, and wettability.

Table 6 shows properties of some representative membranes. While most properties are relevant for all separator membrane types, some properties are more relevant for porous separators, for which the physical and chemical properties of the pore system are crucial in determining the overall performance of the separator. Physical properties such as the average pore size, porosity, and tortuosity play a significant role in determining the separator’s performance. The wettability is a representative chemical property that affects the affinity of the liquid electrolyte to the separator’s pore system. These properties determine key performance indicators such as bubble point pressure, ohmic resistance, and dissolved H_2_ permeability. For example, a smaller average pore size is favorable for gas-tightness but can increase the ohmic resistance. Higher wettability can be achieved by increasing the ratio of inorganic materials in the separator, resulting in reduced ohmic resistance but weaker mechanical stability and higher gas permeability.^336^ Therefore, optimizing the pore system and wettability is crucial since these parameters directly affect both ionic conductivity and gas separation properties.

**Table 6:** Properties of Some Representative State-of-the Art Separators and Membranes^a^

	diaphragms	membranes
conductivity	200 mS/cm for Zirfon in 30 wt % KOH at room temperature^97^	33 mS/cm in 31 wt % KOH, 25 °C, DABCO-modified SEBS^94^
	300 mS/cm Zirfon-type membrane Z80 in 30 wt % KOH^67^	78 mS/cm in 19% KOH, RT, mTPN^121^
	288 mS/cm in 30 wt % KOH for “C100” cerium oxide–PSU composite^58^	93 mS/cm in 25 wt % KOH and 71 mS/cm in 30 wt % KOH for *m*-PBI^95^
	349 mS/cm measured at 200 mA/cm^2^ in 25 wt % KOH at room temperature after 1 month immersion in 25 wt % KOH^50^	248 mS/cm in 25 wt % KOH for PTFE-reinforced gel-PBI^97^
area specific resistance	0.25 Ω cm^2^^97^–0.3 Ω cm^2^^58^ in 30 wt % KOH for 500 μm thick Zirfon Perl	0.65 Ω cm^2^ in 31 wt % KOH, 25 °C, 217 μm thick DABCO-modified SEBS^94^
	0.1–0.47 Ω cm^2^ for 300–600 μm thick Zirfon-type membrane Z80^67^	0.025 Ω cm^2^ in 25 wt % KOH for 65 μm thick PTFE-reinforced gel-PBI^97^
	0.16 Ω cm^2^ in 30 wt % KOH for 460 μm thick “C100” cerium oxide–polysulfone composite^58^	
	0.46 Ω cm^2^ in 30 wt % KOH at 30 °C for 1 mm thick precommercial diaphragm, PPS with inorganic particles^b^	
H_2_ permeability	20 × 10^–12^ mol cm^–1^ s^–1^ bar^–1^ in 30 wt % KOH at 30 °C for 500 μm thick Zirfon Perl UTP^58^	3.0 × 10^–12^ mol cm^–1^ s^–1^ bar^–1^ at 40 °C and 4.2 × 10^–12^ mol cm^–1^ s^–1^ bar^–1^ at 60 °C, 24 wt % KOH, in situ, for PTFE-reinforced gel-PBI^97^
	3 × 10^–12^ mol cm^–1^ s^–1^ bar^–1^ in 30 wt % KOH for 300 μm thick Zirfon-type membrane Z80^67^	4.0 × 10^–12^ mol cm^–1^ s^–1^ bar^–1^ at 40 °C and 6.8 × 10^–12^ mol cm^–1^ s^–1^ bar^–1^ at 60 °C, 24 wt % KOH, in situ, for *m*-PBI^97^
	1.2 × 10^–12^ mol cm^–1^ s^–1^ bar^–1^ in 30 wt % KOH at 30 °C for 460 μm thick “C100” cerium oxide–polysulfone composite^58^	5.6 × 10^–12^ mol cm^–1^ s^–1^ at 40 °C, 20 wt % KOH, in situ, for 150 μm PBI80/P(IB-PEO) membrane^105^
H_2_ crossover	0.09% at 400 mA/cm^2^ and 0.16% at 200 mA/cm^2^, 60 °C, 5 bar, 30 wt % KOH, Zirfon^94^	0.14% at 400 mA/cm^2^ and 0.28% at 200 mA/cm^2^, 60 °C, 24 wt % KOH, PTFE-reinforced gel-PBI^97^
	0.04% at 400 mA/cm^2^ and 0.09% at 200 mA/cm^2^, 60 °C, 24 wt % KOH, Zirfon^97^	0.19% at 400 mA/cm^2^ and 0.30% at 200 mA/cm^2^, 20 wt % KOH, 60 °C, 150 μm PBI80/P(IB-PEO) membrane^105^
	0.081% at 160 mA/cm^2^ at room temperature, 25 wt % KOH^50^	
dimensional stability	<1.5%, 15 min in water at 100 °C, for Zirfon^337^^c^	dry to 1 M KOH: 16% length and thickness swelling for mTPN,^121^ 12% in length^128^
		dry to 1 M KOH @ 30 °C:^128^ 21.7% (FAA3–50), 8.2% (FAA3-PK-75), 10.3% (PiperION PI-15), 10.5% (PiperION PI-20)
bubble point	3.5 bar (500 μm thick Zirfon Perl UTP)^94^	n/a
	1.9 bar (500 μm thick Zirfon Perl UTP)^338^	
	1.5–6 bar for 300–600 μm thick Zirfon-type membrane Z80^67^	
	4 bar for 460 μm thick “C100” cerium oxide–polysulfone composite^58^	
	<1 bar for 1.3 mm thick precommercial diaphragm, PPS with inorganic particles^b^	
mechanical properties	2.1 MPa/-/32%, wetted with KOH^339^	32.2 MPa/80 MPa/96% PTFE-reinforced gel-PBI doped in 25 wt % KOH^97^
(tensile strength/Young’s modulus/elongation at break)	breaking stress (N/mm^2^) 8–14	19 MPa/136 MPa/101% for *m*-PBI doped in 20 wt % KOH^105^
	breaking extension (%) 35–80	
	elastic modulus (N/mm^2^) 22–30^b^	
alkaline stability	Zirfon has a lifetime >5 years under normal operating conditions;^94^ one work reports 16–50% higher resistance due to iron deposits blocking the pores within 130 h of operation at 60 °C^340^	12% weight loss of a PTFE-reinforced gel-PBI 4 weeks in 25 wt % KOH at 80 °C^97^
	constant voltage of approximately 2.3 V during 600 h in 50 wt % KOH at 120 °C	mTPN (Orion Polymer): ≈ 0% IEC loss after 1000 h at 80 °C in 1 M KOH^341^
		MTCP-50: 5.7% conductivity loss after 8000 h in 1 M NaOH at KOH at 80 °C; stable operation in an electrolyzer for 2500 h (1 M KOH, 60 °C, 500 mA cm^–2^, 15 μV/h voltage increase)^257^
		AEMION+ (Ionomr): stable operation in an electrolyzer for 8900 h (1 M KOH, 70 °C, 200 mA cm^–2^, 18 μV/h voltage increase)^151^
		Sustainion grade T (Dioxide Materials): stable operation in an electrolyzer for 12000 h (1 M KOH, 60 °C, 1 A cm^–2^, 0.7 μV/h voltage increase)^131^
wettability	air contact angle (deg) (in water) 126.2^b^	

a The focus was put on highly alkaline conditions, when data was available.

b Data provided by Membrasenz.

c This seems to be shrink.^340^

### 4.2 Through-Plane Conductivity in KOH and ASR

The area resistance (Ω·cm^2^) is the resistance of a membrane sample normalized by multiplication with the sample area. It can be measured through electrochemical impedance spectroscopy which is carried out utilizing an H-type cell.^54^ The prepared separator is placed in between the two half cells, which are filled with KOH solution. Then the impedance is recorded in the frequency range of, e.g., 0.1 MHz to 1 Hz. Electrodes can be nickel, platinum or gold-plated metal discs or meshes. However, if the mesh is close to the membrane, the electrode area will be smaller than the geometrical area of the mesh. At the same time, a close membrane-electrode distance is preferred, to have a significant difference between resistance values of the cell with and without membrane. For thick membranes of a high resistance, the following equation can be used:
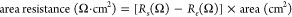
3*R*_*s*_ and *R*_*e*_ are the measured ohmic resistance with separator and without separator, respectively.

For thin membranes having a low resistance, it can be beneficial to measure the cell resistance for several membrane stacks.^97^ Plotting the resistance against the thickness of the membrane stacks (1 membrane, 2 membranes, 3 membranes, etc.) will show a linear trend, for which the *y*-axis intercept represents the sum of other resistances, i.e., empty cell resistance, membrane/electrolyte or membrane/electrode resistance. The assumption made in this measurement is that the membrane/membrane interface has a negligible resistance. In practice the area specific resistance (ASR, in Ω cm^2^) is often plotted against the membrane thickness (in cm), thus obtained slope has a physical meaning of the conductivity reciprocal (S cm^–1^):
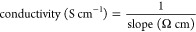
4
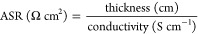
5

Since the resistance of porous diaphragms like Zirfon basically is mainly related to the resistance of the absorbed KOH solution and not to the pore walls, the equation for membrane separator resistance can be modified to
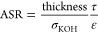
6σ_KOH_ is the conductivity of the KOH solution absorbed in the pores (i.e., related to the concentration of the absorbed solution). ε is the porosity (unitless), and τ is the tortuosity, defined as the squared ratio of the shortest distance through the pore system and the thickness of the porous separator.
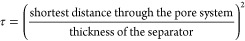
7

### 4.3 True Hydroxide Conductivity of AEM

In-plane conductivity measurements have the advantage that only one membrane sample is needed, because the interfacial resistance between electrode and membrane can be neglected. Commercial cells are available from Scribner (USA, Bekktech BT-110) and Wonatech (Korea, MCC). In these cells, a membrane stripe of about 1 × 4 cm is contacted by 4 platinum wire electrodes. During impedance measurement, an alternating current is applied through the membrane, and the voltage drop over the inner electrodes is measured. If samples are too small to contact all 4 electrodes, but long enough to be covered by the inner, voltage sensing electrodes, the samples can be elongated by plasticizing the ends with a drop of solvent and then gluing other membrane stripes to the end, so that the current can be applied.

Until recently, the rapid absorption of CO_2_ from air hindered reliable conductivity measurements because hydroxide exchanged membranes changed rapidly into the (bi)carbonate form in contact with air.^342^ This was tackled by the Ziv-Dekel method, which first was described for fuel cell membranes in a temperature and humidity-controlled hydrogen atmosphere.^156,343,344^ By applying a direct current over the terminal electrodes, water electrolysis forms hydroxide ions, which move from cathode to anode. After a few hours, all (bi)carbonate ions are purged from the membrane. For electrolysis membranes, the method was simplified.^121,128^ The cell is immersed in a beaker filled with pure water, nitrogen is bubbled through the water to remove dissolved CO_2_, and (bi)carbonate ions are electrochemically purged by applying a direct current. Because the membrane thickness changes during the measurement (hydroxide exchanged membranes absorb more water than (bi)carbonate exchanged membranes),^345^ the thickness used for conductivity calculation is the final thickness after exchange into the hydroxide form.

The limitations of in-plane conductivity measurements are that they can only be done in pure water, that the conductivity in KOH solutions may differ from that in pure water, and that some membranes may show anisotropy, for example reinforced membranes.

At 30 °C, the true in-plane hydroxide conductivity was found to be 58, 49, 71, and 77 mS cm^–1^ for FAA3-50, FAA3-PK-75, PiperION PI-20, and PiperION PI-15, respectively.^128^

### 4.4 Hydrogen Permeability and Crossover

The term of hydrogen gas permeability is specifically used to refer the permeation of hydrogen through the membrane via diffusion and convection mechanisms. The diffusion is driven by the concentration or pressure difference while the convection by the hydraulic pressure gradient between the anode and the cathode. The flux of a permeate is often defined as the volume flow through the membrane per unit area per unit time, in unit of cm^3^/cm^–2^ s^–1^. As the gas volume varies with pressure and temperature, the molar flux in unit of mol cm^–2^ s^–1^ is more practical in use. The hydrogen permeability coefficient or simply permeability is the flux of a permeate through a membrane per unit driving force per unit membrane thickness. With the driving force as the pressure difference across the thickness of the membrane (bar cm^–1^), the hydrogen permeability is hence in unit of mol cm^–1^ s^–1^ bar^–1^.

As mentioned above, the H_2_ crossover is a practical term to refer the overall permeation of hydrogen through the membrane driven by all possible transport mechanisms including the diffusion, convection and electro-osmotic drag, e.g., dissolved hydrogen with the solvated hydroxide ions. The H_2_ crossover is often expressed in equivalent current density (*i*_H_2_crossover_, mA cm^–2^), the same unit related to the production rate of hydrogen and oxygen in electrolyzers. In this way the content of the crossover H_2_ in the anode product O_2_ can be estimated by
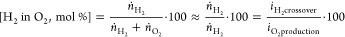
where *ṅ*H_2_ and *ṅ*O_2_ are the mole number of hydrogen and oxygen at the anode side, *i*_H_2_crossover_ is the equivalent current density of hydrogen crossover and *i*_O_2_production_ the operational current density of an electrolyzer. As the H_2_ crossover current density is primarily depending on the pressure difference, the H_2_ content increases when an electrolyzer operates at part load.

**Figure 27:**
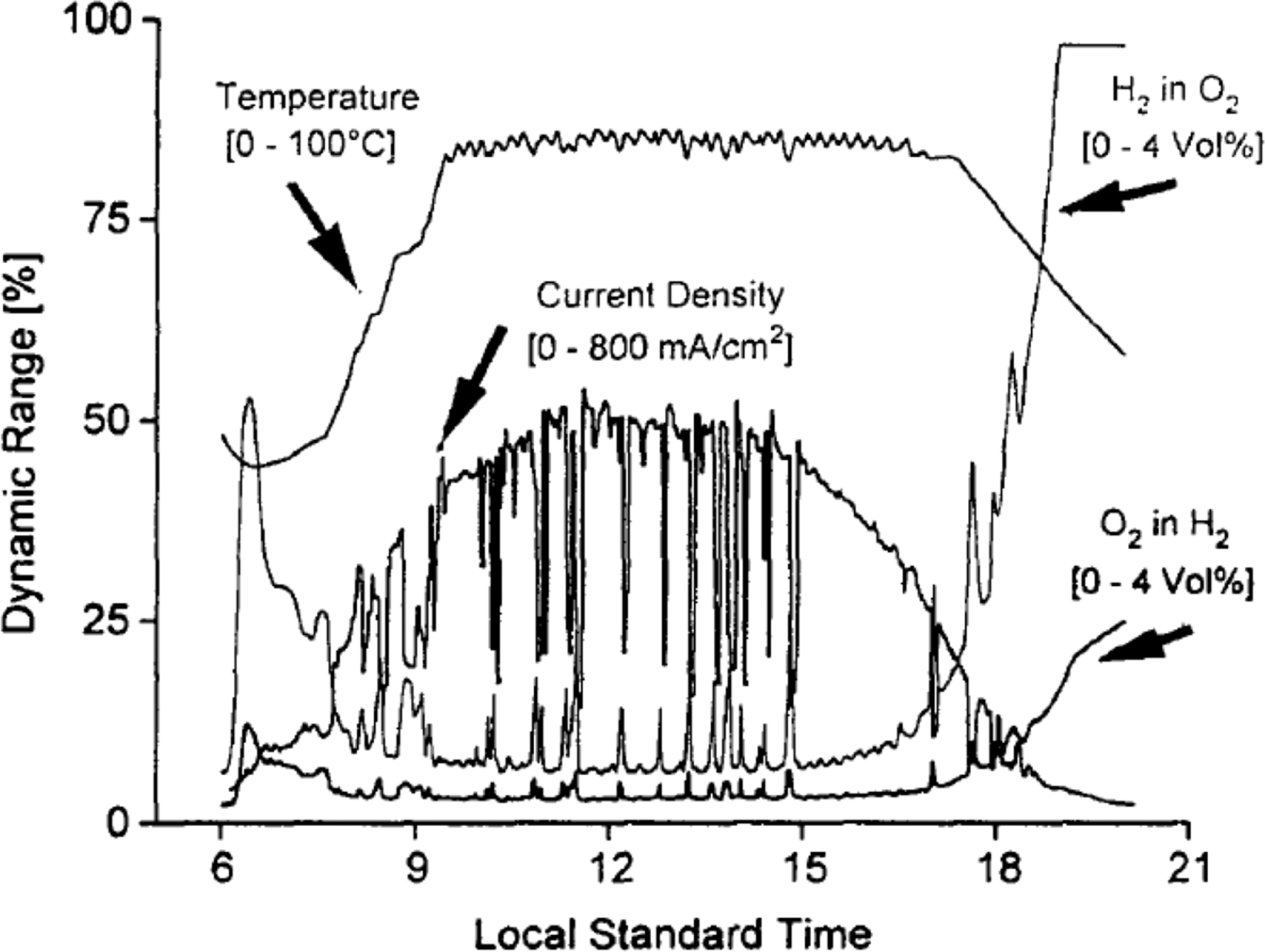
Gas impurities in dependence of current density at solar operation electrolysis within 1 day (24 June 1993). Ten kilowatt alkaline electrolyzer: 20 cells, membranes on the basis of polysulfone are used as separators. Reproduced with permission from reference (31). Copyright 1996 Elsevier, Inc.

The acceptable range for part-load operation of industrial alkaline water electrolyzers is typically 10–40% of the nominal load. Figure 27 illustrates the relationship between current density and the impurities during 1 day of operation of an alkaline electrolyzer powered by solar energy.^31^ It is apparent that at low current densities, gas impurities increase and eventually reach a safety threshold. The lower explosion limits (LEL) and upper explosion limits (UEL) of H_2_/O_2_ mixtures are 3.8 mol % and 95.4 mol % H_2_, respectively, at atmospheric pressure and 80 °C. Gas impurities are primarily attributed to permeation routes, which can be divided into two pathways: diffusive and convective mass transfer mechanisms.^346^

Convective mass transfer occurs due to the circulation of gas-saturated electrolyte throughout the stack and balance of plant. After separating liquid and gas phase, the consumed cathode–electrolyte is still saturated with H_2_, while the consumed anode electrolyte is saturated with O_2_. To compensate for the difference in electrolyte concentration caused by the electrode reactions, the gas-saturated electrolytes are mixed together, and then are evenly pumped back to the cathode and anode. These continuous circulations lead to the overall saturation of the electrolyte with H_2_ and O_2_, thereby making gas mixture from convective mass transfer inevitable. The exact mode of electrolyte circulation can partially control mixing of gases by convective mass transfer.^347^

The diffusive mass transfer mechanism is the transport of gas-saturated electrolyte through the porous separator driven by differential pressure between cathode and anode.

The molar hydrogen permeation flux density (Φ_H_2__^Darcy^, mol s^–1^ cm^–2^) caused by the absolute difference pressure (bar) across the separator thickness *d* (cm) can be expressed using the given formula:
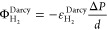
8H_2_ gas permeability driven by differential pressures, ε_H_2__^Darcy^ (mol cm^–1^ s^–1^ bar^–1^) is defined as
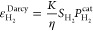
9where *K* implies the electrolyte permeability (cm^2^) which is largely governed by the average pore size of the separator. η is viscosity of the electrolyte (bar s). *S*_H_2__ represents the solubility of H_2_ gas in the electrolyte (mol m^–3^ bar^–1^), and *P*_H_2__^cat^ indicates the partial H_2_ pressure on the cathodic side (bar). The complete measurement method and setup are provided elsewhere.^348^

These equations imply that the diffusive mass transfer is mainly governed by the separator properties, electrolyte permeability (*K*) and separator thickness *d*. The Zirfon UTP 500 series exhibits an average pore size of 150 nm, which is expected to enable the permeation of gas-saturated electrolyte across the separator, resulting in an unsatisfactory gas permeability. The diffusive mass transfer can be reduced by developing separators with smaller pore sizes. Recently, a research group from Seoultech was able to successfully fabricate separators with reduced average pore size by optimizing the preparation conditions and additives.

The transport of gas-saturated electrolyte through the separator also increases when the operation pressure increases because both the value of the absolute pressure difference and the solubility of H_2_ gas in the electrolyte increase. Therefore, pressurized alkaline systems show narrower partial load ranges (40–100%) compared to low pressure systems (20–100%).^337^

### 4.5 Electrolyte Permeability

Electrolyte permeability (L cm^–2^ s^–1^ bar^–1^) is the electrolyte flux through the separator (L cm^–2^ s^–1^) normalized by the absolute pressure difference between both sides of the separator (bar). Commercial separators (Zirfon UTP 500 series) exhibit an average pore size of ∼150 nm, through which the dissolved gases in the electrolyte can diffuse driven by the absolute differential pressure between the two chambers. This contaminates the evolved gas of the opposite cell and has influence on the lower partial load range of alkaline electrolyzer.

**Figure 28:**
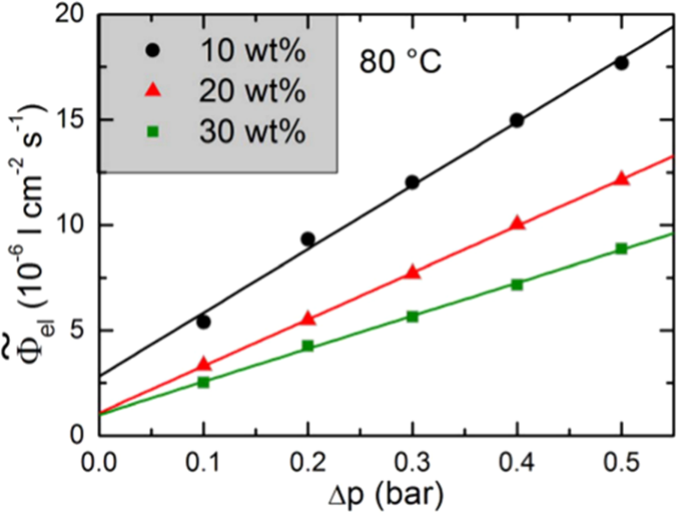
Volumetric permeation flux density of electrolyte through Zirfon sample as a function of the absolute pressure difference at a cell temperature of 80 °C. Reproduced with permission from reference (348). Copyright 2016, The Electrochemical Society.

To measure the electrolyte permeability, the separator sample is inserted between cell chambers which are filled with KOH solution of the desired concentration at a certain temperature (e.g., 25–90 °C). One portion of the cell chamber is pressurized from 1.1 to 1.5 bar with an inert gas. The volume or mass of the electrolyte that permeates through the separator (L cm^–2^ s^–1^) at a certain differential pressure is measured and plotted. The slope of the fitted lines to the data can be used to calculate the electrolyte permeability (Figure 28).

It is widely assumed that gas crossover in the electrolyzer does not occur by break-through of gas bubbles, but by transport of dissolved gases.^349^ Therefore, the electrolyte permeability can be used to estimate the flux of dissolved gases in the electrolyte, and thus the hydrogen permeability (H_2_ mol cm^–1^ s^–1^ bar^–1^) by using Henry’s law.^348^Henry’s law
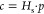
10in which *c* is the concentration of the dissolved gas, *H*_s_ is the Henry solubility (a constant, mol m^–3^ Pa^–1^), and *p* the pressure of the gas component, e.g., H_2_, in the gas phase. *H*_s_ values for H_2_ in 1, 10, 20, and 30 wt % KOH can be found in ref (348).

### 4.6 Dimensional Stability

Dimensional stability is typically measured form the dry to the wet state. However, in an ideal electrolyzer, this dimensional change only occurs during assembly, and then never again, unless system operation is seriously disrupted. What can be observed during operation is swelling as a response to temperature changes.

Changing dimensions from the dry state to the wet state are problematic because the swelling is likely to result in a wrinkled membrane, and the expanding membrane most probably is also pushed into the electrode structure, which could trigger the growth of pin holes or cracks. In addition, if strong compression between the electrodes hinders swelling, the membrane resistance may be higher than expected from ex-situ measurements. In principle, membrane swelling can be tackled by assembling a wet membrane. The challenge here is that membranes lose all absorbed water within just a few minutes,^322^ and constant spraying may work well for small research systems but will be challenging if large areas need to be wetted. Currently, industrial AEM WE systems seem to use an active area <300 cm^2^, but this is expected to increase to 1000 cm^2^ until 2050. In contrast to current AEM WE systems, AWE systems already now reach active areas of 1–3 m^2^.^335^ To tackle this issue, it was suggested to preswell membranes in a solution of ethylene glycol in water (e.g., 50% was found to be a good value for FAA3 membranes). After some minutes in open air, the membranes reach a quasi-constant weight because only the water contents evaporates. These membranes can be easily assembled into electrolyzers and show a minimized dimensional change from begin of assembly to begin of operation, when the high boiling solvent is exchanged for water.^322^ As an additional feature, the preswollen membranes showed slightly improved performance in the electrolyzer, presumably because the preswelling enlarged and merged the hydrophilic domains.

**Figure 29:**
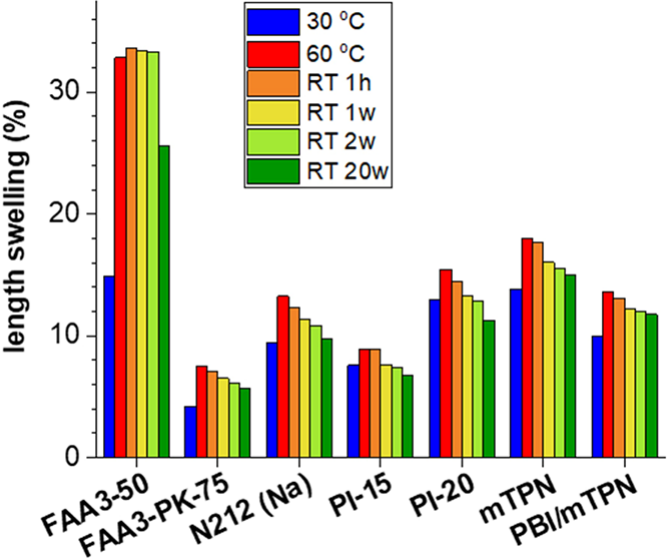
Length swelling of different AEM (chloride form) and Nafion from dry to wet state at 30 °C, then to wet state at 60 °C, and after cooling to room temperature after 1 h, 1 week, 2 weeks and 20 weeks. PI-15 and PI-20 are PiperION membranes from Versogen, and FAA3-PK-75, PI-15 and PBI/mTPN membranes are reinforced.^128^

In general, it appears that swelling from dry to wet is much more pronounced than when the temperature is increased from 30 to 60 °C in the wet state (Figure 29). Interestingly, all membranes showed hysteresis, and did not return to their initial length at 30 °C when the temperature decreased from 60 °C to room temperature. This seems to be due to anisotropic swelling and isotropic shrinking, which indicates that excessive swelling removes oriented morphological features.

### 4.7 Bubble Point of Zirfon Type Separators

The bubble point is an important parameter for porous diaphragms and defines the pressure, at which air can push water (or KOH solution) out of a pore. In other words, when an electrolysis system operates at differential pressure, the bubble point should be well above the pressure difference to prevent breakthrough of hydrogen or oxygen. In a typical measurement system, the porous separator is clamped between two support grids. On one side, the membrane is contacted with water, on the other side, air is supplied and its pressure is increased in small steps. When the pressure reaches the bubble point, air overcomes the capillary forces inside of the widest pore, and a regular chain of gas bubbles evolves from the membrane. Some authors also define the bubble point as the point when a constant gas flow of 0.1 L min^–1^ is reached.^94^ A related standard, developed for filtration membranes, is ASTM F316. For Zirfon PERL, a bubble point of ca. 2.5 bar was reported,^67^ which is within the specified value of 2 ± 1 bar.^52^

The diameter of the limiting pore size can be calculated by the Young–Laplace equation:^350^
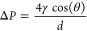
11where Δ*P* is the pressure difference, γ the interfacial surface tension, θ the contact angle between the interface and the pore wall, and *d* is the pore diameter.

### 4.8 Mechanical Strength

Mechanical stability is very important for fuel cell membranes, which repeatedly experience humidity changing during operation and respond by swelling and shrinking. This is less an issue for water electrolyzers, in which membranes (ideally) are always contacted by liquid water. Potentially, changes in temperature will result in dimensional changes, but it was found that the effect is less pronounced than one may expect when considering the dry to wet swelling, which is usually reported. In fact, AEM fully swollen in hot water do shrink when the temperature is decreased but do not reach the initial starting point they had in cold water.^128^ Another reason high mechanical strength is needed is the large area of industrial scale installations, which can reach up to 3 m^2^.^351^ This large area results in large stresses already during membrane handling.

While the mechanical properties of hydrophobic polymers used in diaphragms (e.g., polyphenylene sulfide or polysulfone) show low dependence on the humidity, hydrophilic materials like AEM show a strong dependence and thus ideally should be measured in hot water.^128^ Therefore, the low tensile strength of Zirfon, which is in the range of ≈2 MPa,^339,352^ should be compared with the properties of AEM in water controlled to a temperature relevant for electrolyzers, like 60–80 °C. Furthermore, the water contents of hydroxide exchanged AEMs is higher than that of the chloride exchanged ions, which are commonly preferred, because the hydroxide form membranes rapidly absorb CO_2_ from air.

For ISM, creep could play an important role, similar to highly phosphoric acid doped PBI membranes used in fuel cells, which strongly suffer from creep.^353,354^ Similar investigations for KOH doped membranes were not conducted yet but clearly are of interest.

### 4.9 Alkaline Stability Tests

For AEM, especially those of the first generations, alkaline stability was easy to assess. Membranes were immersed in for example 1–2 M KOH at 60 °C, sometimes even just room temperature, for a few days or even just hours.^355,356^ Typically the conductivity decreased noticeably, because of the low alkaline stability of quaternary ammonium groups. Some membranes became so brittle during the test that they fell apart. The breaking points were determined to be hydrolyzable groups in the polymer backbone like ethers^114^ or imidazolium ions with easily accessible C2 positions.^141−143^ The newest generation of membranes is much more stable but still shows signs of degradation.^341,357^ In contrast to AEMs developed for AEM water electrolysis or fuel cells, AWE separators should last for years. Therefore, alkaline stability tests should be conducted by immersing membrane samples in highly concentrated KOH solutions (e.g., 25–30 wt %) at an elevated temperature of at least 60–80 °C^97,105^ and for a duration of several months. For future AWE, even stabilities measured at 110 to 120 °C would be of interest. Currently, stable operation with state-of-the art Zirfon membranes is limited to 110 °C, because the polysulfone starts to degrade in contact with hot KOH and oxygen.^12^ To prevent absorption of CO_2_ and thus decreased alkalinity over time, the test solutions should be exchanged in regular intervals. The vials containing the solutions should be made of PTFE because glass will dissolve under these conditions and solutions in polypropylene vials were observed to form precipitates, easier due to leached out additives or rapid permeation of CO_2_. Usually, tests are done in air, but stability may be higher in inert atmosphere. The reason is not clear, but there are indications that reactive oxygen species like superoxides may contribute to the observed alkaline degradation.^358−360^ Main properties to be monitored in alkaline stability tests are weight and dimensional changes, conductivity and mechanical properties.

### 4.10 Wettability

Wettability refers to the degree to which a solid surface prefers contact with one fluid over another. It is typically quantified by measuring the apparent contact angle between the electrolyte and the porous separator. However, the contact angle of composite separators cannot be measured directly because the electrolyte quickly permeates the separator through macropores. Therefore, the contact angle can be determined indirectly using Washburn’s method.^361^ First, the separator is cut into sample stripes, e.g., 50 mm length and 20 mm width, and then dried. Subsequently, the samples are immersed in KOH solution and the weight change over time is recorded. The contact angle value can then be obtained by applying the Washburn formula. Furthermore, the elemental concentrations (atomic%) on the top surface of separators can be analyzed using X-ray photoelectron spectroscopy (XPS).

## 5 Design Strategies for Future Separators

### 5.1 Zirfon-Type Diaphragms

The Young–Laplace equation shows that in order to achieve a high bubble point pressure (BPP), it is necessary to produce a separator with smaller pores.^63^ The use of separators with smaller pore diameters increases the BPP and the friction of electrolyte permeation. In addition, a high tortuosity will further reduce the gas crossover,^94^ however, at the cost of increased resistance. Therefore, to reduce differential pressure-driven electrolyte permeability, separators should have small pore diameters and high tortuosity.^348^ From the Table 7, it is clear that the separators with smaller average pore diameter have a strong relationship with higher BPP and the reduced electrolyte permeability.

**Table 7:** Characteristics and Performance Data of Porous Separators

separator	average pore diameter (nm)	tortuosity	thickness (μm)	wettability in 30 wt % KOH (deg)	ohmic resistance (ohm cm^2^ @25 °C 30 wt % KOH)	bubble point pressure (bar)	H_2_ permeability (10^–12^ mol/(cm sec bar))	references
Zirfon UTP 500	140	2.04	500 ± 50	83.41	0.30 ± 0.05	3 ± 1	20 ± 1	AGFA^52^
Zirfon UTP 500+	115	1.34	500 ± 50	81.22	0.2 ± 0.2	3 ± 1	15 ± 1	
Zirfon UTP 200	123	1.75	220 ± 50		0.1 ± 0.2	1.5 ± 0.5	18 ± 1	
Z85_300μm	55	0.68	300 ± 30		0.1 ± 0.2	6.6 ± 0.7	3.8 ± 0.5	H.I. Lee et al. (2020)^67^
Z85_500μm	76.7	0.92	450 ± 50	80.18	0.14 ± 0.2	7.1 ± 0.7	0.46 ± 0.5	
Z82_CNC3	45.9	0.55	450 ± 50	72.04	0.1 ± 0.2	9 ± 0.7	0.55 ± 0.5	J.W. Lee et al. (2022)^66^
Z80_CNC5	91.7	0.44	450 ± 50	70.97	0.08 ± 0.2	6.6 ± 0.7	0.64 ± 0.5	
C100 (CeO_2_ 100 nm)	77.6	0.99	450 ± 50	80.68	0.16 ± 0.2	4 ± 0.7	1.2 ± 0.5	J.W. Lee et al. (2020)^58^
C40 (CeO_2_ 50 nm)	62.6	1.31	450 ± 50	75.79	0.18 ± 0.2	5 ± 0.7	0.69 ± 0.5	
TiO_2_ (40 nm)	113.5	0.86	450 ± 50	81	0.15 ± 0.2	3.7 ± 0.7	5.8 ± 0.5	M.F. Ali et al. (2023)^49^
TiO_2_ (100 nm)	55.7	1.15	450 ± 50	76	0.14 ± 0.2	3.1 ± 0.7	8.12 ± 0.5	
Z30TA70	125.1	1.09	450 ± 50		0.14 ± 0.2	1.5 ± 0.7	11.2 ± 0.5	M.F. Ali et al. (2022)^59^
Z5TA95	82	1.06	450 ± 50		0.16 ± 0.2	1.5 ± 0.7	10.7 ± 0.5	

The ohmic drop is divided into the contributions originating from the area resistance of ion conduction through the separator and the area resistance arising from other sources. The primary factor influencing the ohmic resistance of a cell is the thickness of the separator. As indicated in Table 7, thinner separators exhibit lower ohmic resistance. Moreover, the ohmic resistance can be lowered by the fabrication of higher wettability. Burnat et al. discovered that the conductivity of the top layer is crucial in reducing the resistivity of membranes prepared through the phase inversion process.^54^

As shown in eq 6, porosity, tortuosity, and thickness are directly related to the ohmic resistance of a separator, which agrees with data in Table 7. It is clearly seen that the tortuosity values of Zirfon UTP membranes are above 1.5, which is higher than those of Seoultech separators. This suggests the presence of a high concentration of polymer layers, which is reflected by the tortuosity value. Therefore, future separators should be designed to be thin with a uniform top layer and a low tortuosity, while maintaining mesopores with higher wettability. This could be achieved by symmetrically coating a thin PPS fabric mesh with a slurry of a polymer and nanoparticles that sustain a strong interaction during the blading step. The formation of a dense and polymer-rich top layer should be avoided during the coagulation stage, in order to achieve a more uniform and porous structure throughout the separator. By optimizing these parameters, it may be possible to develop a separator with improved performance and stability for use in high-performance AWE.

Costa and Grimes first suggested the “zero gap” configuration in 1967. This method uses mesh electrodes on either side of a microporous gas separator, with the electrodes firmly pressed against the diaphragm to minimize the area resistance through the solution. This approach employs a catalyst-coated substrate (CCS) fabrication method that is straightforward and suitable for large-scale production. The substrate material acts as both the electrode and the porous transport layer and can take on various forms such as mesh, foam, and sheet. However, nanoparticle catalysts with higher reactivity are difficult to be directly applied to practical and industrial applications in CCS cells because direct deposition techniques like electrochemical deposition, heat treatment, or plasma spray deposition are not easily scaled up despite of promising performances in a lab-scale.^74^ If nonconducting binders are employed to adhere the catalyst onto the substrate, these binders tend to add high charge transfer resistance at higher current densities in electrocatalysis, and often cause the catalyst to peel off from the substrate over extended periods of operation.^362^ Therefore, the design of the zero-gap cell necessitates the development of deposition techniques that can offer scalability, high performance, and durability.

**Figure 30:**
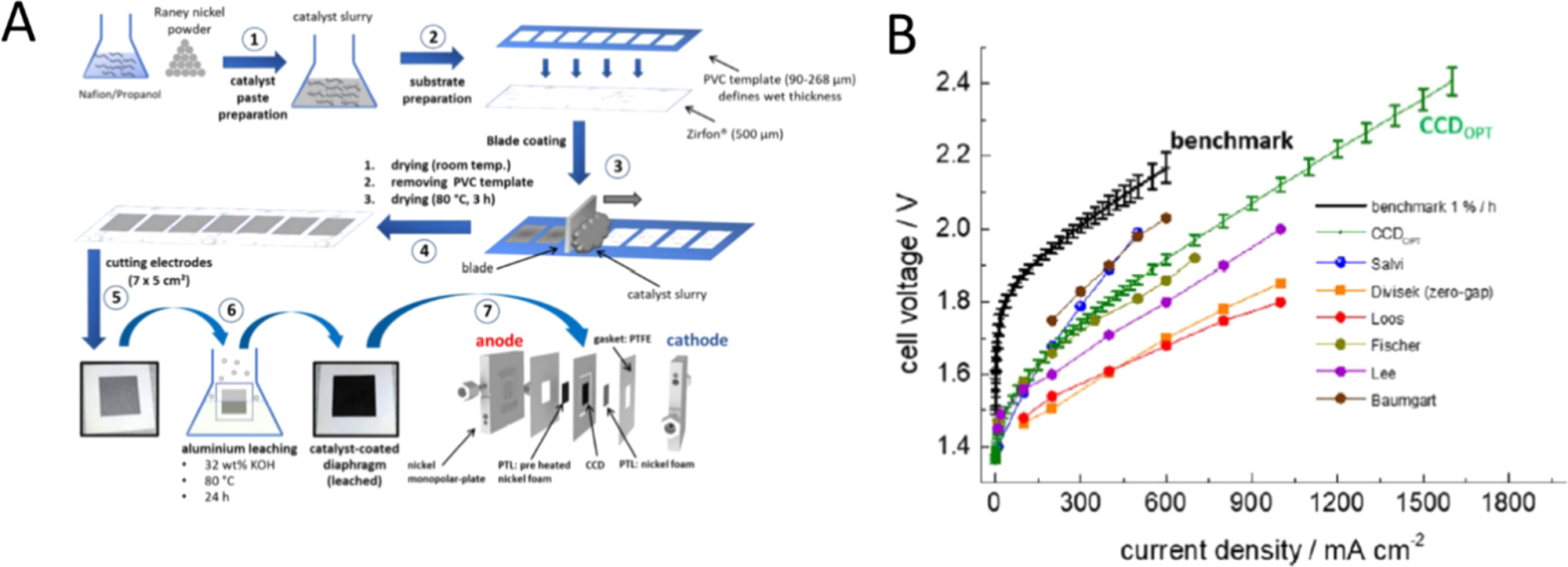
(A) Sketch of the CCM fabrication. 1: Preparation of the catalyst slurry. 2: Cutting the PVC template and assembly on the Zirfon to define the electrode geometry. 3: Blade-coating of catalyst slurry on the diaphragm (Zirfon). 4: Drying the CCM under ambient conditions, removing the PVC template from the Zirfon substrate and hot drying at 80 °C in a furnace under air conditions. 5: Cutting the electrodes with a punch machine into seven separate electrodes. 6: Leaching the aluminum in 32 wt % KOH solution and sodium tartrate at 80 °C for 24 h. 7: Mounting in an alkaline single cell for electrochemical characterization on the cathode side. (B) Polarization curves of zero-gap and nonzero-gap electrodes based on Raney nickel compared to the benchmark and CCM with optimum loading. Reproduced with permission from reference (74). Copyright 2022, The Electrochemical Society.

The catalyst coated membrane (CCM) is another MEA manufacturing process in which a catalyst ink is directly coated onto a membrane. CCM fabrications can be achieved through a variety of methods, including direct wet spraying, decal transfer, painting, screen and inkjet printing, doctor blade coating, and layer-by-layer techniques. These methods were shown to increase the total number of active sites on the MEA, resulting in high performance at low catalyst loading. Karacan et al.^74^ successfully produced a CCM-based MEA using a porous separator for alkaline electrolyzers. They directly coated Raney Ni catalysts onto Zirfon separators as the cathode with Nafion ionomer and used an annealed Ni foam as the anode electrode (Figure 30(A)). The Nafion ionomer acted mainly as a binder and less as an ionic conductor since it is a cation-conducting electrolyte. The catalyst was coated using the blade coating technique, which is similar to industrial roll-to-roll (R2R) coating. The authors varied the catalyst loading, catalyst layer thickness, and weight ratio of catalyst and Nafion to demonstrate the CCM electrode concept. MEAs with low Raney nickel mass loading (∼21 mg cm^–2^) showed higher overpotential due to the reduced active surface area per geometric area. The MEAs with higher Raney nickel mass loading (∼42 mg cm^–2^) exhibited an increased overpotential due to aggregation of catalysts on the electrode surface and the poor contact between the porous transport layer (PTL) and the catalyst layer. Remarkably, an alkaline single cell at an optimum loading (∼36 mg cm^–2^) showed a voltage of 1.7 V at around 200 mA cm^–2^, which is comparable to the performance of the advanced catalyst-deposited CCS cells (Figure 30(B)). This result demonstrated that CCM using the porous separator successfully reduced the kinetic overvoltage at the electrodes by enhancing catalyst utilization. However, the optimized cell showed a degradation rate of 22 μA cm^–2^ h^–1^ after 1000 h at a cell voltage of 2 V. The degradation of the catalytic activity was due to the loss of the catalyst over time via the accumulation of large, unstably attached catalyst particles on the electrode surface. Future work on more active catalyst particles, optimization of the binder, and pore engineering may lead to further improvements in the CCM method. These efforts represent an important step toward highly efficient and scalable alkaline water electrolysis.

While most systems are operated at temperatures of up to 80 °C, higher temperatures are known to reduce the voltage required to split water. Therefore, it is of interest to look at membrane stability also beyond 100 °C. For example, in 30 wt % KOH and above 100 °C, commercial Zirfon appears to be unstable.^12^

### 5.2 AEM and ISM

MEA fabrication is also important for AEM and ISM. The dense nature of AEM and ISM potentially allows to print membranes directly onto the electrodes. Such direct membrane deposition results in an even more intimate contact between membrane and electrodes than by standard CCM methods, because the applied polymers will enter into the electrode pores, membrane and electrode will be mechanically anchored to each other, improving the interfacial strength. Simultaneously, the nonplanar membrane surface may also aid to lower interfacial resistances and improve mass transfer across the membrane. Direct membrane deposition has been shown for PEM WE,^363^ but most research focused on fuel cell MEAs. Polymer layers were deposited by inkjet printing^364^ or ultrasonic-spray deposition.^365^ Ink jet printing was shown also to work in combination with porous reinforcements.^366^ This indicates that direct deposition of reinforced membranes and diaphragms on electrodes is technically feasible.

The use of reinforcements seems to be inevitable, considering the large cell areas of industrial scale AWE (up to 3 m^2^) and AEM WE (up to 300 cm^2^, possibly 1,000 cm^2^ until 2050).^335^ Future research should investigate more closely the support|matrix interface, and stronger interactions, for example ionic or covalent bonds^121^ or mechanical anchoring points by increased surface roughness should be introduced. In addition, an overall increased support surface by thinning the fiber diameter will be helpful.

With respect to the chemistry of AEMs, it appears that poly(norbornene)s are not broadly investigated yet for use as AEM in electrolyzers. A literature search showed 64 hits for norbornene and “anion exchange membrane”, just 7 hits for further refinement to “electrolysis”, and most of these references deal with ionomer development. It could be that the bulky structure of the monomers results in increased polymer free volume and thus larger gas crossover, which would be very attractive for use as electrode ionomer binder,^367,368^ but less suitable for use as AEM; this needs further investigation. However, synthesis of a variety of poly(norbornene)-based AEMs is straightforward and promises alkaline stable backbones functionalized with various quaternary ammonium groups.^136,138,369^

The main bottleneck for AEMs remains the low alkaline stability of the quaternary ammonium groups. Strategies to further improve the stability could be the synthesis of new motifs, for example the hypothetical structure **8** shown in section 3.3, Figure 13, or the development of cofunctional groups which prevent nucleophilic degradation of the QA groups by shielding or chelating the ammonium groups or reducing the nucleophilicity of hydroxide ions in the vicinity of the ammonium groups.

Ion solvating membranes show very promising performances and recently improved stability. We believe that novel ISM chemistries and the use of reinforcing support materials will advance their stability further, and offer a broadly open field for polymer chemists and membrane scientists. In this light, it should be noted that it is a common understanding in the community that PEM selectively conduct cations and AEM selectively conduct anions. What is often ignored in this discussion is that Donnan exclusion is ideal for membranes in pure water, but fails when the ionic strength of the feed solution reaches that of the membrane.^370,371^ In fact, even concentrations of just about 1 M KOH can lead to significant absorption of co-ions.^120,372,373^ For example, Che et al. reported that a poly(arylene piperidinium)-based AEM separating a 3 M ammonium sulfate solution and a 3 M sulfuric acid solution had a larger proton permeability than Nafion 115.^220^ In other words, AEM and PEM lose ion selectivity and start to function as a diaphragm. This opens the field for membranes which are not or less ion-selective. Membranes which have features of proton exchange membranes and ISM, for example sulfonated PBI, can become a field of future research.^29,101^ Such membranes are basically ISM and potentially will have higher conductivity than AEM or traditional ISM like KOH doped *m*-PBI, and avoid quaternary ammonium groups. In principle, aromatic sulfonic acid groups can be substituted by hydroxides, but at least on a preparative scale, very harsh reaction conditions are required for this degradation.^374,375^ This potential degradation pathway will need to be investigated in future research.

Enapter, as an example, recommends to use their AEMWE with 0.2 M KOH feed solution, to balance performance (favors high KOH concentration) and lifetime of the AEM (favors low KOH concentration). Use of quaternary-ammonium-free ISM instead of AEM, combined with an increased KOH concentration, would simultaneously enhance performance and lifetime of “AEMWE” systems. It could well be that AEMs for use in alkaline feed solutions will soon start to be substituted by advanced ISM, which would finally solve the problems associated with the low alkaline stability of quaternary ammonium groups.

In steam electrolyzers, early ion solvating membranes failed. This could be due to strong plasticization or accelerated hydrolysis promoted by the high temperatures, and more detailed investigations are needed. A general hurdle is that mechanical properties are typically tested by stress–strain tests in air. While stress–strain tests in temperature-controlled water were recently shown for chloride exchanged AEM,^128^ tests in hot KOH solution, as it would be needed for diaphragms and even more so for ISM, are currently not an option, due to material limitations and safety concerns.

## 6 Conclusions

Traditionally, alkaline electrolysis involves using electrodes pressed onto a porous separator filled with a liquid alkaline electrolyte. Pore engineering is critical for ionic conductivity and gas separation properties as electrolyte transport and gas permeation through the pores affect efficiency and product purity. The formation of a polymer-rich layer on the top of the separator reduces pore size and wettability, increasing the resistance. It is highly desirable to synthesize the separator as thin as possible with a uniform top layer and low tortuosity, while maintaining mesopores with higher wettability. The cells using the porous separators with optimized pore systems represented remarkable performance, suggesting that PGM-free catalyst AEL electrolysis can compete with PEM electrolysis with PGM catalysts. The CCM cell using a porous separator reduced kinetic overvoltage by enhancing catalyst utilization but experienced the loss of catalyst over time. Future work on more active catalysts, optimization of the binder, and pore engineering may lead to further improvements in the CCM cell using a porous separator; to improve the electrolyzer performance, operation at higher temperatures, up to 120 °C is desired, but polysulfone used in commercial Zirfon starts to show signs of degradation at these harsh conditions.

Most AEM research focused on AEM WE operating with pure water, but these systems require the use of ionomer binders. This raises additional issues, like phenyl oxidation and consequent loss of mobile hydroxide ions. Furthermore, there is a consensus that electrolyzer performance increases when the KOH concentration in the feed solution increases. Therefore, current commercial AEM WE use 1% KOH feed solutions, and with advanced AEM showing improved alkaline stability, AEM WE will probably use higher KOH concentrations to balance performance and lifetime, and AWE and AEM WE fields will merge.

ISM emerge as a promising alternative to AEM and porous diaphragms. Already 1000 h operation without failure were reported, and alternative chemistries beyond KOH doped PBI start to be investigated. Due to the soft nature of KOH swollen polymers, reinforcement strategies will help to increase lifetime. While *m*-PBI has a very low conductivity in 2 M KOH, sulfonated PBI derivatives showed conductivity >100 mS cm^–1^ in 1 M KOH; this suggests that future ISM may show excellent performance in the AEM WE range.

Regardless which membrane types will be used in the future: Not much is known about the lifetime of membranes under real operating conditions. We know from the fuel cell field that degradation can proceed slowly and linear, until the trend is interrupted by sudden, unexpected failure. Some good AEMs already were in use for one year without significant degradation, and Enapter predicts >35,000 h of operation for their electrolysis stacks. Diaphragms have been even in use for over 10 years in AWE. While this is very promising, reliable methods to predict the lifetime are lacking. For this, the exact degradation pathways and the stressors need to be identified, and accelerated stress test protocols need to be developed. For AEM, the degradation pathways already are well understood, but alkaline stability depends strongly on the solvation of hydroxides, and the stability ranking of two membranes can change based on the applied test protocol. Research into this direction should accompany research on new materials.

## List of Abbreviations

AEL: alkaline electrolyzer
AEM: anion exchange membrane
AEM WE: anion exchange membrane water electrolysis
ASD: 5-azoniaspiro[4.5]decane
ASU: 6-azoniaspiro[5.5]undecane
ATRP: atom transfer radical polymerization
AWE: alkaline water electrolysis
BPP: bubble point pressure
CCM: catalyst-coated membrane
CCS: catalyst-coated substrate
CNCs: cellulose nanocrystals
DABCO: 1,4-diazabicyclo[2.2.2]octane
DCM: dichloromethane
ETFE: poly(ethylene-*co*-tetrafluoroethylene)
ePTFE: expanded PTFE (i.e., porous PTFE)
HHV: higher heating value
IEC: ion exchange capacity
ISM: ion solvating membrane
LEL: lower explosion limit
MEA: membrane electrode assembly
NMP: *N*-methyl-2-pyrrolidone
PBI: polybenzimidazole
PE: polyethylene
PEEK: poly(ether ether ketone)
PEM: proton exchange membrane
PEM WE: proton exchange membrane water electrolysis
PFA: perfluoroalkoxy alkanes
PGM: platinum group metals
PP: polypropylene
PPS: polyphenylene sulfide
PSU: polysulfone
PTFE: polytetrafluoroethylene
PVA: poly(vinyl alcohol)
PVDF: poly(vinylidene fluoride)
PVP: polyvinylpyrrolidone
QA: quaternary ammonium
SES: poly(styrene-*block*-ethylene-*block*-styrene)
SEBS: poly(styrene-*block*-(ethylene-*co*-butylene)-*block*-styrene)
TFSA: trifluorosulfonic acid
TMA: trimethylamine
UEL: upper explosion limit
WE: water electrolysis
YSZ: yttria-stabilized zirconia
ZTA: zirconia toughened alumina

## References

[ref1] https://unfccc.int/process-and-meetings/the-paris-agreement (accessed 2023-07-27).

[ref2] Modisha P.; Bessarabov D. Aromatic Liquid Organic Hydrogen Carriers for Hydrogen Storage and Release. Curr. Opin. Green Sustain. Chem. 2023, 42, 100820 10.1016/j.cogsc.2023.100820.

[ref3] Niermann M.; Beckendorff A.; Kaltschmitt M.; Bonhoff K. Liquid Organic Hydrogen Carrier (Lohc) - Assessment Based on Chemical and Economic properties. Int. J. Hydrog. Energy 2019, 44, 6631–6654. 10.1016/j.ijhydene.2019.01.199.

[ref4] Chen Y. Global Potential of Algae-Based Photobiological Hydrogen Production. Energy Environ. Sci. 2022, 15, 2843–2857. 10.1039/D2EE00342B.

[ref5] Sharma A.; Arya S. K. Hydrogen from Algal Biomass: A Review of Production Process. Biotechnol. Rep. 2017, 15, 63–69. 10.1016/j.btre.2017.06.001. PMC5491395 28702371

[ref6] Song H.; Yang G.; Xue P.; Li Y.; Zou J.; Wang S.; Yang H.; Chen H. Recent Development of Biomass Gasification for H_2_ Rich Gas Production. Appl. Energy Combust. Sci. 2022, 10, 100059 10.1016/j.jaecs.2022.100059.

[ref7] Antonini C.; Treyer K.; Moioli E.; Bauer C.; Schildhauer T. J.; Mazzotti M. Hydrogen from Wood Gasification with CCs - a Techno-Environmental Analysis of Production and Use as Transport Fuel. Sustain. Energy Fuels 2021, 5, 2602–2621. 10.1039/D0SE01637C.

[ref8] Song H.; Luo S.; Huang H.; Deng B.; Ye J. Solar-Driven Hydrogen Production: Recent Advances, Challenges, and Future Perspectives. ACS Energy Lett. 2022, 7, 1043–1065. 10.1021/acsenergylett.1c02591.

[ref9] Steinfeld A. Solar Thermochemical Production of Hydrogen — A Review. Sol. Energy 2005, 78, 603–615. 10.1016/j.solener.2003.12.012.

[ref10] Oudejans D.; Offidani M.; Constantinou A.; Albonetti S.; Dimitratos N.; Bansode A. A Comprehensive Review on Two-Step Thermochemical Water Splitting for Hydrogen Production in a Redox Cycle. Energies 2022, 15, 3044 10.3390/en15093044.

[ref11] David M.; Ocampo-Martínez C.; Sánchez-Peña R. Advances in Alkaline Water Electrolyzers: A Review. J. Energy Storage 2019, 23, 392–403. 10.1016/j.est.2019.03.001.

[ref12] Ehlers J. C.; Feidenhans’l A. A.; Therkildsen K. T.; Larrazábal G. O. Affordable Green Hydrogen from Alkaline Water Electrolysis: Key Research Needs from an Industrial Perspective. ACS Energy Lett. 2023, 8, 1502–1509. 10.1021/acsenergylett.2c02897.

[ref13] http://conference.ing.unipi.it/ichs2005/Papers/120001.pdf (accessed 2023-08-23);

[ref14] Schröder V.; Emonts B.; Janßen H.; Schulze H. P. Explosion Limits of Hydrogen/Oxygen Mixtures at Initial Pressures up to 200 bar. Chem. Eng. Technol. 2004, 27, 847–851. 10.1002/ceat.200403174.

[ref15] Carmo M.; Fritz D. L.; Mergel J.; Stolten D. A Comprehensive Review on PEM Water Electrolysis. Int. J. Hydrog. Energy 2013, 38, 4901–4934. 10.1016/j.ijhydene.2013.01.151.

[ref16] Minke C.; Suermann M.; Bensmann B.; Hanke-Rauschenbach R. Is Iridium Demand a Potential Bottleneck in the Realization of Large-Scale PEM Water Electrolysis?. Int. J. Hydrog. Energy 2021, 46, 23581–23590. 10.1016/j.ijhydene.2021.04.174.

[ref17] Jeong H.-Y.; Oh J.; Yi G. S.; Park H.-Y.; Cho S. K.; Jang J. H.; Yoo S. J.; Park H. S. High-Performance Water Electrolyzer with Minimum Platinum Group Metal Usage: Iron Nitride-Iridium Oxide Core-Shell Nanostructures for Stable and Efficient Oxygen Evolution Reaction. Appl. Catal. B: Environ. 2023, 330, 122596 10.1016/j.apcatb.2023.122596.

[ref18] Lohmann R.; Cousins I. T.; DeWitt J. C.; Gluge J.; Goldenman G.; Herzke D.; Lindstrom A. B.; Miller M. F.; Ng C. A.; Patton S.; Scheringer M.; Trier X.; Wang Z. Are Fluoropolymers Really of Low Concern for Human and Environmental Health and Separate from Other PFAS?. Environ. Sci. Technol. 2020, 54, 12820–12828. 10.1021/acs.est.0c03244. 33043667 PMC7700770

[ref19] https://www.eionet.europa.eu/etcs/etc-wmge/products/etcwmge-reports/fluorinated-polymers-in-a-low-carbon-circular-and-toxic-free-economy (accessed 2024-01-08).

[ref20] https://echa.europa.eu/hot-topics/perfluoroalkyl-chemicals-pfas (accessed 2023-08-17).

[ref21] Henkensmeier D.; Najibah M.; Harms C.; Žitka J.; Hnát J.; Bouzek K. Overview: State-of-the Art Commercial Membranes for Anion Exchange Membrane Water Electrolysis. J. Electrochem. Energy Convers. Storage 2021, 18, 024001 10.1115/1.4047963.

[ref22] https://handbook.enapter.com/electrolyzer/el21a/downloads/ELE21-MAN-000EN_REV03.pdf (accessed 2023-01-30).

[ref23] Dekel D. R.; Amar M.; Willdorf S.; Kosa M.; Dhara S.; Diesendruck C. E. Effect of Water on the Stability of Quaternary Ammonium Groups for Anion Exchange Membrane Fuel Cell Applications. Chem. Mater. 2017, 29, 4425–4431. 10.1021/acs.chemmater.7b00958.

[ref24] Gilliam R.; Graydon J.; Kirk D.; Thorpe S. A Review of Specific Conductivities of Potassium Hydroxide Solutions for Various Concentrations and Temperatures. Int. J. Hydrog. Energy 2007, 32, 359–364. 10.1016/j.ijhydene.2006.10.062.

[ref25] Li D.; Park E. J.; Zhu W.; Shi Q.; Zhou Y.; Tian H.; Lin Y.; Serov A.; Zulevi B.; Baca E. D.; Fujimoto C.; Chung H. T.; Kim Y. S. Highly Quaternized Polystyrene Ionomers for High Performance Anion Exchange Membrane Water Electrolyzers. Nat. Energy 2020, 5, 378–385. 10.1038/s41560-020-0577-x.

[ref26] Lindquist G. A.; Gaitor J. C.; Thompson W. L.; Brogden V.; Noonan K. J. T.; Boettcher S. W. Oxidative Instability of Ionomers in Hydroxide Exchangemembrane Electrolyzers. ChemRxiv 2023, 10.26434/chemrxiv-2023-7w3rz.

[ref27] Chen Q.; Huang Y.; Hu X.; Hu B.; Liu M.; Bi J.; Liu L.; Li N. A Novel Ion-Solvating Polymer Electrolyte Based on Imidazole-Containing Polymers for Alkaline Water Electrolysis. J. Membr. Sci. 2023, 668, 121186 10.1016/j.memsci.2022.121186.

[ref28] Hu B.; Huang Y.; Liu L.; Hu X.; Geng K.; Ju Q.; Liu M.; Bi J.; Luo S.; Li N. A Stable Ion-Solvating PBI Electrolyte Enabled by Sterically Bulky Naphthalene for Alkaline Water Electrolysis. J. Membr. Sci. 2022, 643, 120042 10.1016/j.memsci.2021.120042.

[ref29] Dayan A.; Trisno M. L. A.; Yang C.; Kraglund M. R.; Almind M. R.; Hjelm J.; Jensen J. O.; Aili D.; Park H. S.; Henkensmeier D. Quaternary Ammonium-Free Membranes for Water Electrolysis with 1 M KOH. Adv. Energy Mater. 2023, 13, 2302966 10.1002/aenm.202302966.

[ref30] Crandall W.; Harada Y. Improved Asbestos Matrices for Alkaline Fuel Cells. SAE Technical Paper 1975, 750466 10.4271/750466.

[ref31] Kerres J.; Eigenberger G.; Reichle S.; Schramm V.; Hetzel K.; Schnurnberger W.; Seybold I. Advanced Alkaline Electrolysis with Porous Polymeric Diaphragms. Desalination 1996, 104, 47–57. 10.1016/0011-9164(96)00025-2.

[ref32] Choi M.; Lee J.; Ryu S.; Ku B.-C. Fabrication and Applications of Polyphenylene Sulfide (PPS) Composites: A Short Review. Compos. Res. 2020, 33, 91–100. 10.7234/composres.2020.33.3.091.

[ref33] Gao Y.; Zhou X.; Zhang M.; Lyu L.; Li Z. Polyphenylene Sulfide-Based Membranes: Recent Progress and Future Perspectives. Membranes 2022, 12, 924 10.3390/membranes12100924. 36295683 PMC9607490

[ref34] Zhang J.; Zhu C.; Xu J.; Wu J.; Yin X.; Chen S.; Zhu Z.; Wang L.; Li Z.-C. Enhanced Mechanical Behavior and Electrochemical Performance of Composite Separator by Constructing Crosslinked Polymer Electrolyte Networks on Polyphenylene Sulfide Nonwoven Surface. J. Membr. Sci. 2020, 597, 117622 10.1016/j.memsci.2019.117622.

[ref35] Xing J.; Xu Z.; Deng B. Enhanced Oxidation Resistance of Polyphenylene Sulfide Composites Based on Montmorillonite Modified by Benzimidazolium Salt. Polymers 2018, 10, 83 10.3390/polym10010083. 30966117 PMC6415019

[ref36] Yang L.; Takeda M.; Zhang Y. (Toray Industries Inc.) Patent EP2947709B1, 2014.

[ref37] Renaud R.; Leroy R. Separator Materials for Use in Alkaline Water Electrolyzers. Int. J. Hydrog. Energy 1982, 7, 155–166. 10.1016/0360-3199(82)90142-2.

[ref38] Xu D.; Liu B.; Zhang G.; Long S.; Wang X.; Yang J. Effect of Air Plasma Treatment on Interfacial Shear Strength of Carbon Fiber-Reinforced Polyphenylene Sulfide. High Perf. Polym. 2016, 28, 411–424. 10.1177/0954008315585012.

[ref39] Anagreh N.; Dorn L.; Bilke-Krause C. Low-Pressure Plasma Pretreatment of Polyphenylene Sulfide (PPS) Surfaces for Adhesive Bonding. Int. J. Adhes. Adhes. 2008, 28, 16–22. 10.1016/j.ijadhadh.2007.03.003.

[ref40] Quan D.; Deegan B.; Byrne L.; Scarselli G.; Ivanković A.; Murphy N. Rapid Surface Activation of Carbon Fibre Reinforced PEEK and PPS Composites by High-Power UV-Irradiation for the Adhesive Joining of Dissimilar Materials. Compos. Part A Appl. Sci. Manuf. 2020, 137, 105976 10.1016/j.compositesa.2020.105976.

[ref41] Gao Y.; Li Z.; Cheng B.; Su K. Superhydrophilic Poly(p-Phenylene Sulfide) Membrane Preparation with Acid/Alkali Solution Resistance and Its Usage in Oil/Water Separation. Sep. Purification Technol. 2018, 192, 262–270. 10.1016/j.seppur.2017.09.065.

[ref42] Wong I.; Ho C. M. Surface Molecular Property Modifications for Poly(Dimethylsiloxane) (PDMS) Based Microfluidic Devices. Microfluid Nanofluidics 2009, 7, 291–306. 10.1007/s10404-009-0443-4. 20357909 PMC2847407

[ref43] Hoadley J. K.; Ginter J. F. Polyphenylene Sulfide (PPS) as a Membrane in Electrolysis Cells. International Conference On Environmental Systems 1996, 10.4271/961438.

[ref44] Manabe A.; Domon H.; Kosaka J.; Hashimoto T.; Okajima T.; Ohsaka T. Study on Separator for Alkaline Water Electrolysis. J. Electrochem. Soc. 2016, 163, F3139–F3145. 10.1149/2.0191611jes.

[ref45] Giuffré L.; Modica G.; Pagani A.; Marisio G. (Fratelli Testori S.P.A.) Patent US4895634A, 1988.

[ref46] Wang X.; Li Z.; Zhang M.; Fan T.; Cheng B. Preparation of a Polyphenylene Sulfide Membrane from a Ternary Polymer/Solvent/Non-Solvent System by Thermally Induced Phase Separation. RSC Adv. 2017, 7, 10503–10516. 10.1039/C6RA28762J.

[ref47] Godshall G. F.; Spiering G. A.; Crater E. R.; Moore R. B. Low-Density, Semicrystalline Poly(Phenylene Sulfide) Aerogels Fabricated Using a Benign Solvent. ACS Appl. Polym. Mater. 2023, 5, 7994–8004. 10.1021/acsapm.3c01171.

[ref48] Asghar M. R.; Anwar M. T.; Naveed A.; Zhang J. A Review on Inorganic Nanoparticles Modified Composite Membranes for Lithium-Ion Batteries: Recent Progress and Prospects. Membranes 2019, 9, 78 10.3390/membranes9070078. 31269768 PMC6680444

[ref49] Ali M. F.; Cho H.-S.; Bernäcker C. I.; Albers J.; Young-Woo C.; Kim M.; Lee J. H.; Lee C.; Lee S.; Cho W.-C. A Study on the Effect of TiO_2_ Nanoparticle Size on the Performance of Composite Separators in Alkaline Water Electrolysis. J. Membr. Sci. 2023, 678, 121671 10.1016/j.memsci.2023.121671.

[ref50] Stojadinovic J.; Mantia F. L. Woven or nonwoven web. WO2016162417A1, 2016.

[ref51] Schalenbach M.; Lueke W.; Stolten D. Hydrogen Diffusivity and Electrolyte Permeability of the Zirfon Perl Separator for Alkaline Water Electrolysis. J. Electrochem. Soc. 2016, 163, F1480–F1488. 10.1149/2.1251613jes.

[ref52] https://www.agfa.com/specialty-products/wp-content/uploads/sites/8/2020/06/TDS_ZIRFON_PERL_UTP_500_20200525.pdf (accessed 2023-08-03).

[ref53] Zheng X. X.; Böttger A. J.; Jansen K. M. B.; van Turnhout J.; van Kranendonk J. Aging of Polyphenylene Sulfide-Glass Composite and Polysulfone in Highly Oxidative and Strong Alkaline Environments. Front. Mater. 2020, 7, 610440 10.3389/fmats.2020.610440.

[ref54] Burnat D.; Schlupp M.; Wichser A.; Lothenbach B.; Gorbar M.; Züttel A.; Vogt U. F. Composite Membranes for Alkaline Electrolysis Based on Polysulfone and Mineral Fillers. J. Power Sources 2015, 291, 163–172. 10.1016/j.jpowsour.2015.04.066.

[ref55] Otero J.; Sese J.; Michaus I.; Santa Maria M.; Guelbenzu E.; Irusta S.; Carrilero I.; Arruebo M. Sulphonated Polyether Ether Ketone Diaphragms Used in Commercial Scale Alkaline Water Electrolysis. J. Power Sources 2014, 247, 967–974. 10.1016/j.jpowsour.2013.09.062.

[ref56] Wan L.; Xu Z.; Wang B. G. Green Preparation of Highly Alkali-Resistant PTFE Composite Membranes for Advanced Alkaline Water Electrolysis. Chem. Eng. J. 2021, 426, 131340 10.1016/j.cej.2021.131340.

[ref57] Sandstede G.; Wurster R. In Modern Aspects of Electrochemistry; White R. E.; Bockris J. O. M.; Conway B. E., Eds.; Springer: Boston, 1995.

[ref58] Lee J. W.; Lee C.; Lee J. H.; Kim S. K.; Cho H. S.; Kim M.; Cho W. C.; Joo J. H.; Kim C. H. Cerium Oxide-Polysulfone Composite Separator for an Advanced Alkaline Electrolyzer. Polymers 2020, 12, 2821 10.3390/polym12122821. 33261186 PMC7759930

[ref59] Ali M. F.; Lee H. I.; Bernäcker C. I.; Weissgarber T.; Lee S.; Kim S. K.; Cho W. C. Zirconia Toughened Alumina-Based Separator Membrane for Advanced Alkaline Water Electrolyzer. Polymers 2022, 14, 1173 10.3390/polym14061173. 35335503 PMC8951763

[ref60] Seetharaman S.; Ravichandran S.; Davidson D. J.; Vasudevan S.; Sozhan G. Polyvinyl Alcohol Based Membrane as Separator for Alkaline Water Electrolyzer. Sep. Sci. Technol. 2011, 46, 1563–1570. 10.1080/01496395.2011.575427.

[ref61] Chatzichristodoulou C.; Allebrod F.; Mogensen M. B. High Temperature Alkaline Electrolysis Cells with Metal Foam Based Gas Diffusion Electrodes. J. Electrochem. Soc. 2016, 163, F3036–F3040. 10.1149/2.0051611jes.

[ref62] Boom R. M.; Wienk I. M.; van den Boomgaard T.; Smolders C. A. Microstructures in Phase Inversion Membranes. Part 2. The Role of a Polymeric Additive. J. Membr. Sci. 1992, 73, 277–292. 10.1016/0376-7388(92)80135-7.

[ref63] Vermeiren P. H.; Leysen R.; Beckers H.; Moreels J. P.; Claes A. The Influence of Manufacturing Parameters on the Properties of Macroporous Zirfon (R) Separators. J. Porous Mater. 2008, 15, 259–264. 10.1007/s10934-006-9084-0.

[ref64] Mulder M.; Winterton J. In Basic Principle of Membrane Technology; Kluwer Academic Publisher: Dordrecht, 1991.

[ref65] Aerts P.; Kuypers S.; Genne I.; Leysen R.; Mewis J.; Vankelecom I. F. J.; Jacobs P. A. Polysulfone-Zro_2_ Surface Interactions. The Influence on Formation, Morphology and Properties of Zirfon-Membranes. J. Phys. Chem. B 2006, 110, 7425–7430. 10.1021/jp053976c. 16599520

[ref66] Lee J. W.; Lee J. H.; Lee C.; Cho H. S.; Kim M.; Kim S. K.; Joo J. H.; Cho W. C.; Kim C. H. Cellulose Nanocrystals-Blended Zirconia/Polysulfone Composite Separator for Alkaline Electrolyzer at Low Electrolyte Contents. Chem. Eng. J. 2022, 428, 131149 10.1016/j.cej.2021.131149.

[ref67] Lee H. I.; Mehdi M.; Kim S. K.; Cho H. S.; Kim M. J.; Cho W. C.; Rhee Y. W.; Kim C. H. Advanced Zirfon-Type Porous Separator for a High-Rate Alkaline Electrolyzer Operating in a Dynamic Mode. J. Membr. Sci. 2020, 616, 118541 10.1016/j.memsci.2020.118541.

[ref68] Lee H. I.; Cho H.-S.; Kim S. K.; Kim M.; Kim C.-H.; Cho W.-C.; Rhee Y. W. Polymer-Ceramic Composite Diaphragm for Reducing Dissolved Hydrogen Permeability in Alkaline Water Electrolysis. ECS Meeting Abstr. 2020, MA2020-02, 2404–2404. 10.1149/MA2020-02382404mtgabs.

[ref69] Karacan C.; Lohmann-Richters F. P.; Keeley G. P.; Scheepers F.; Shviro M.; Muller M.; Carmo M.; Stolten D. Challenges and Important Considerations When Benchmarking Single-Cell Alkaline Electrolyzers. Int. J. Hydrog. Energy 2022, 47, 4294–4303. 10.1016/j.ijhydene.2021.11.068.

[ref70] Alam A.; Park C.; Lee J.; Ju H. Comparative Analysis of Performance of Alkaline Water Electrolyzer by Using Porous Separator and Ion-Solvating Polybenzimidazole Membrane. Renew. Energy 2020, 166, 222–233. 10.1016/j.renene.2020.11.151.

[ref71] Kraglund M. R.; Carmo M.; Schiller G.; Ansar S. A.; Aili D.; Christensen E.; Jensen J. O. Ion-Solvating Membranes as a New Approach Towards High Rate Alkaline Electrolyzers. Energy Environ. Sci. 2019, 12, 3313–3318. 10.1039/C9EE00832B.

[ref72] Ju W. B.; Heinz M. V. F.; Pusterla L.; Hofer M.; Fumey B.; Castiglioni R.; Pagani M.; Battaglia C.; Vogt U. F. Lab-Scale Alkaline Water Electrolyzer for Bridging Material Fundamentals with Realistic Operation. ACS Sustain. Chem. Eng. 2018, 6, 4829–4837. 10.1021/acssuschemeng.7b04173.

[ref73] Shiva Kumar S.; Ramakrishna S. U. B.; Krishna S. V.; Srilatha K.; Devi B. R.; Himabindu V. Synthesis of Titanium (Iv) Oxide Composite Membrane for Hydrogen Production through Alkaline Water Electrolysis. South African J. Chem. Eng. 2018, 25, 54–61. 10.1016/j.sajce.2017.12.004.

[ref74] Karacan C.; Lohmann-Richters F. P.; Shviro M.; Keeley G. P.; Muller M.; Carmo M.; Stolten D. Fabrication of High Performing and Durable Nickel-Based Catalyst Coated Diaphragms for Alkaline Water Electrolyzers. J. Electrochem. Soc. 2022, 169, 054502 10.1149/1945-7111/ac697f.

[ref75] Schalenbach M.; Tjarks G.; Carmo M.; Lueke W.; Mueller M.; Stolten D. Acidic or Alkaline? Towards a New Perspective on the Efficiency of Water Electrolysis. J. Electrochem. Soc. 2016, 163, F3197–F3208. 10.1149/2.0271611jes.

[ref76] Vermeiren P. Evaluation of the Zirfons Separator for Use in Alkaline Water Electrolysis and Ni-H_2_ Batteries. Int. J. Hydrog. Energy 1998, 23, 321–324. 10.1016/S0360-3199(97)00069-4.

[ref77] Kuleshov N. V.; Kuleshov V. N.; Dovbysh S. A.; Grigoriev S. A.; Kurochkin S. V.; Millet P. Development and Performances of a 0.5 KW High-Pressure Alkaline Water Electrolyzer. Int. J. Hydrog. Energy 2019, 44, 29441–29449. 10.1016/j.ijhydene.2019.05.044.

[ref78] Kuleshov V. N.; Kurochkin S. V.; Kuleshov N. V.; Blinov D. V.; Grigorieva O. Y. Electrode-Diaphragm Assembly for Alkaline Water Electrolysis. J. Phys.: Conf. Ser. 2020, 1683, 052011 10.1088/1742-6596/1683/5/052011.

[ref79] Kim S.; Han J. H.; Yuk J.; Kim S.; Song Y.; So S.; Lee K. T.; Kim T.-H. Highly Selective Porous Separator with Thin Skin Layer for Alkaline Water Electrolysis. J. Power Sources 2022, 524, 231059 10.1016/j.jpowsour.2022.231059.

[ref80] Divisek J.; Schmitz H. A Bipolar Cell for Advanced Alkaline Water Electrolysis. Int. J. Hydrog. Energy 1982, 7, 703–710. 10.1016/0360-3199(82)90018-0.

[ref81] Aili D.; Kraglund M. R.; Tavacoli J.; Chatzichristodoulou C.; Jensen J. O. Polysulfone-Polyvinylpyrrolidone Blend Membranes as Electrolytes in Alkaline Water Electrolysis. J. Membr. Sci. 2020, 598, 117674 10.1016/j.memsci.2019.117674.

[ref82] Hackemuller F. J.; Borgardt E.; Panchenko O.; Muller M.; Bram M. Manufacturing of Large-Scale Titanium-Based Porous Transport Layers for Polymer Electrolyte Membrane Electrolysis by Tape Casting. Adv. Eng. Mater. 2019, 21, 1801201 10.1002/adem.201801201.

[ref83] de Groot M. T.; Vreman A. W. Ohmic Resistance in Zero Gap Alkaline Electrolysis with a Zirfon Diaphragm. Electrochim. Acta 2021, 369, 137684 10.1016/j.electacta.2020.137684.

[ref84] Kiaee M.; Cruden A.; Chladek P.; Infield D. Demonstration of the Operation and Performance of a Pressurised Alkaline Electrolyzer Operating in the Hydrogen Fuelling Station in Porsgrunn, Norway. Energy Convers. Manage. 2015, 94, 40–50. 10.1016/j.enconman.2015.01.070.

[ref85] Khan M. A.; Al-Shankiti I.; Ziani A.; Idriss H. Demonstration of Green Hydrogen Production Using Solar Energy at 28% Efficiency and Evaluation of Its Economic Viability. Sustain. Energy Fuels 2021, 5, 1085–1094. 10.1039/D0SE01761B.

[ref86] Ursua A.; Barrios E. L.; Pascual J.; San Martin I.; Sanchis P. Integration of Commercial Alkaline Water Electrolyzers with Renewable Energies: Limitations and Improvements. Int. J. Hydrog. Energy 2016, 41, 12852–12861. 10.1016/j.ijhydene.2016.06.071.

[ref87] Wijmans J. G.; Smolders C. A. In Synthetic Membranes: Science, Engineering and Applications; Bungay P. M., Lonsdale H. K., Pinho M. N., Eds.; Springer: Dordrecht, 1986.

[ref88] Xing B.; Savadogo O. Hydrogen/Oxygen Polymer Electrolyte Membrane Fuel Cells (PEMFCs) Based on Alkaline-Doped Polybenzimidazole (PBI). Electrochem. Commun. 2000, 2, 697–702. 10.1016/S1388-2481(00)00107-7.

[ref89] Aili D.; Yang J.; Jankova K.; Henkensmeier D.; Li Q. From Polybenzimidazoles to Polybenzimidazoliums and Polybenzimidazolides. J. Mater. Chem. A 2020, 8, 12854–12886. 10.1039/D0TA01788D.

[ref90] Aili D.; Hansen M. K.; Renzaho R. F.; Li Q.; Christensen E.; Jensen J. O.; Bjerrum N. J. Heterogeneous Anion Conducting Membranes Based on Linear and Crosslinked KOH Doped Polybenzimidazole for Alkaline Water Electrolysis. J. Membr. Sci. 2013, 447, 424–432. 10.1016/j.memsci.2013.07.054.

[ref91] Zeng L.; Zhao T. S.; An L.; Zhao G.; Yan X. H. Physicochemical Properties of Alkaline Doped Polybenzimidazole Membranes for Anion Exchange Membrane Fuel Cells. J. Membr. Sci. 2015, 493, 340–348. 10.1016/j.memsci.2015.06.013.

[ref92] Aili D.; Jankova K.; Han J.; Bjerrum N. J.; Jensen J. O.; Li Q. Understanding Ternary Poly(Potassium Benzimidazolide)-Based Polymer Electrolytes. Polymer 2016, 84, 304–310. 10.1016/j.polymer.2016.01.011.

[ref93] Kraglund M. R.; Aili D.; Jankova K.; Christensen E.; Li Q.; Jensen J. O. Zero-Gap Alkaline Water Electrolysis Using Ion-Solvating Polymer Electrolyte Membranes at Reduced KOH Concentrations. J. Electrochem. Soc. 2016, 163, F3125–F3131. 10.1149/2.0161611jes.

[ref94] Brauns J.; Schönebeck J.; Kraglund M. R.; Aili D.; Hnát J.; Žitka J.; Mues W.; Jensen J. O.; Bouzek K.; Turek T. Evaluation of Diaphragms and Membranes as Separators for Alkaline Water Electrolysis. J. Electrochem. Soc. 2021, 168, 014510 10.1149/1945-7111/abda57.

[ref95] Konovalova A.; Kim H.; Kim S.; Lim A.; Park H. S.; Kraglund M. R.; Aili D.; Jang J. H.; Kim H.-J.; Henkensmeier D. Blend Membranes of Polybenzimidazole and an Anion Exchange Ionomer (FAA3) for Alkaline Water Electrolysis: Improved Alkaline Stability and Conductivity. J. Membr. Sci. 2018, 564, 653–662. 10.1016/j.memsci.2018.07.074.

[ref96] Aili D.; Jankova K.; Li Q.; Bjerrum N. J.; Jensen J. O. The Stability of Poly(2,2′-(m-Phenylene)-5,5′-Bibenzimidazole) Membranes in Aqueous Potassium Hydroxide. J. Membr. Sci. 2015, 492, 422–429. 10.1016/j.memsci.2015.06.001.

[ref97] Trisno M. L. A.; Dayan A.; Lee S. J.; Egert F.; Gerle M.; Kraglund M. R.; Jensen J. O.; Aili D.; Roznowska A.; Michalak A.; Park H. S.; Razmjooei F.; Ansar S.-A.; Henkensmeier D. Reinforced Gel-State Polybenzimidazole Hydrogen Separators for Alkaline Water Electrolysis. Energy Environ. Sci. 2022, 15, 4362–4375. 10.1039/D2EE01922A.

[ref98] Serhiichuk D.; Patniboon T.; Xia Y.; Kraglund M. R.; Jensen J. O.; Hansen H. A.; Aili D. Insight into the Alkaline Stability of Arylene-Linked Bis-Benzimidazoles and Polybenzimidazoles. ACS Appl. Polym. Mater. 2023, 5, 803–814. 10.1021/acsapm.2c01769.

[ref99] Thomas O. D.; Soo K. J.; Peckham T. J.; Kulkarni M. P.; Holdcroft S. A Stable Hydroxide-Conducting Polymer. J. Am. Chem. Soc. 2012, 134, 10753–10756. 10.1021/ja303067t. 22712732

[ref100] Aili D.; Wright A. G.; Kraglund M. R.; Jankova K.; Holdcroft S.; Jensen J. O. Towards a Stable Ion-Solvating Polymer Electrolyte for Advanced Alkaline Water Electrolysis. J. Mater. Chem. A 2017, 5, 5055–5066. 10.1039/C6TA10680C.

[ref101] Hu X.; Liu M.; Huang Y.; Liu L.; Li N. Sulfonate-Functionalized Polybenzimidazole as Ion-Solvating Membrane toward High-Performance Alkaline Water Electrolysis. J. Membr. Sci. 2022, 663, 121005 10.1016/j.memsci.2022.121005.

[ref102] Patniboon T.; Hansen H. A. Degradation of Polybenzimidazole in Alkaline Solution with First-Principles Modeling. Electrochim. Acta 2021, 398, 139329 10.1016/j.electacta.2021.139329.

[ref103] Xia Y.; Rajappan S. C.; Serhiichuk D.; Kraglund M. R.; Jensen J. O.; Aili D. Poly(Vinyl Alcohol-Co-Vinyl Acetal) Gel Electrolytes for Alkaline Water Electrolysis. J. Membr. Sci. 2023, 680, 121719 10.1016/j.memsci.2023.121719.

[ref104] Hu X.; Hu B.; Niu C.; Yao J.; Liu M.; Tao H.; Huang Y.; Kang S.; Geng K.; Li N. An Operationally Broadened Alkaline Water Electrolyzer Enabled by Highly Stable Poly(Oxindole Biphenylene) Ion-Solvating Membranes. Nat. Energy 2024, xx 10.1038/s41560-023-01447-w.

[ref105] Makrygianni M.; Aivali S.; Xia Y.; Kraglund M. R.; Aili D.; Deimede V. Polyisatin Derived Ion-Solvating Blend Membranes for Alkaline Water Electrolysis. J. Membr. Sci. 2023, 669, 121331 10.1016/j.memsci.2022.121331.

[ref106] O’Brien T. F.; Bommaraju T. V.; Hine F. Handbook of Chlor-Alkali Technology; Springer: New York, 2005.

[ref107] Tang H.; Aili D.; Tufa R. A.; Kraglund M. R.; Wu Q.; Pan C.; Cleemann L. N.; Li Q. Anion Conductivity of Cation Exchange Membranes in Aqueous Supporting Electrolytes. Solid State Ion. 2022, 383, 115984 10.1016/j.ssi.2022.115984.

[ref108] Coury L. Conductance Measurements Part 1: Theory. Curr. Separat. 1999, 18, 91–96.

[ref109] Yeo R. S.; McBreen J.; Kissel G.; Kulesa F.; Srinivasan S. Perfluorosulphonic Acid (Nafion) Membrane as a Separator for an Advanced Alkaline Water Electrolyzer. J. Appl. Electrochem. 1980, 10, 741–747. 10.1007/BF00611277.

[ref110] Aili D.; Hansen M. K.; Andreasen J. W.; Zhang J.; Jensen J. O.; Bjerrum N. J.; Li Q. Porous Poly(Perfluorosulfonic Acid) Membranes for Alkaline Water Electrolysis. J. Membr. Sci. 2015, 493, 589–598. 10.1016/j.memsci.2015.06.057.

[ref111] Lee W.; Konovalova A.; Tsoy E.; Park G.; Henkensmeier D.; Kwon Y. Alkaline Naphthoquinone-Based Redox Flow Batteries with a Crosslinked Sulfonated Polyphenylsulfone Membrane. Int. J. Energy Res. 2022, 46, 12988–13002. 10.1002/er.8079.

[ref112] Merle G.; Wessling M.; Nijmeijer K. Anion Exchange Membranes for Alkaline Fuel Cells: A Review. J. Membr. Sci. 2011, 377, 1–35. 10.1016/j.memsci.2011.04.043.

[ref113] https://pubs.usgs.gov/periodicals/mcs2022/mcs2022-platinum.pdf (accessed 2023-08-03).

[ref114] Arges C. G.; Ramani V. Two-Dimensional NMR Spectroscopy Reveals Cation-Triggered Backbone Degradation in Polysulfone-Based Anion Exchange Membranes. Proc. Natl. Acad. Sci. U.S.A. 2013, 110, 2490–2495. 10.1073/pnas.1217215110. 23335629 PMC3574919

[ref115] Wu X.; Scott K. CCu_x_Co_3-x_O_4_ (0 ≤ X < 1) Nanoparticles for Oxygen Evolution in High Performance Alkaline Exchange Membrane Water Electrolyzers. J. Mater. Chem. 2011, 21, 12344–12351. 10.1039/c1jm11312g.

[ref116] Leng Y.; Chen G.; Mendoza A. J.; Tighe T. B.; Hickner M. A.; Wang C. Y. Solid-State Water Electrolysis with an Alkaline Membrane. J. Am. Chem. Soc. 2012, 134, 9054–9057. 10.1021/ja302439z. 22587676

[ref117] Xiao L.; Zhang S.; Pan J.; Yang C.; He M.; Zhuang L.; Lu J. First Implementation of Alkaline Polymer Electrolyte Water Electrolysis Working Only with Pure Water. Energy Environ. Sci. 2012, 5, 7869–7871. 10.1039/c2ee22146b.

[ref118] Varcoe J. R.; Atanassov P.; Dekel D. R.; Herring A. M.; Hickner M. A.; Kohl P. A.; Kucernak A. R.; Mustain W. E.; Nijmeijer K.; Scott K.; Xu T.; Zhuang L. Anion-Exchange Membranes in Electrochemical Energy Systems. Energy Environ. Sci. 2014, 7, 3135–3191. 10.1039/C4EE01303D.

[ref119] https://www.caplinq.com/aemion-%E2%84%A2-anion-exchange-membrane-reinforced-75micron-af2-hwp8-75-x.htm (accessed 2023-01-19).

[ref120] Kreuer K.-D.; Münchinger A. Fast and Selective Ionic Transport: From Ion-Conducting Channels to Ion Exchange Membranes for Flow Batteries. Annu. Rev. Mater. Res. 2021, 51, 21–46. 10.1146/annurev-matsci-080619-010139.

[ref121] Najibah M.; Tsoy E.; Khalid H.; Chen Y.; Li Q.; Bae C.; Hnát J.; Plevová M.; Bouzek K.; Jang J. H.; Park H. S.; Henkensmeier D. PBI Nanofiber Mat-Reinforced Anion Exchange Membranes with Covalently Linked Interfaces for Use in Water Electrolyzers. J. Membr. Sci. 2021, 640, 119832 10.1016/j.memsci.2021.119832.

[ref122] Tang Z.; Svoboda R.; Lawton J. S.; Aaron D. S.; Papandrew A. B.; Zawodzinski T. A. Composition and Conductivity of Membranes Equilibrated with Solutions of Sulfuric Acid and Vanadyl Sulfate. J. Electrochem. Soc. 2013, 160, F1040–F1047. 10.1149/2.083309jes.

[ref123] Chempath S.; Einsla B. R.; Pratt L. R.; Macomber C. S.; Boncella J. M.; Rau J. A.; Pivovar B. S. Mechanism of Tetraalkylammonium Headgroup Degradation in Alkaline Fuel Cell Membranes. J. Phys. Chem. C 2008, 112, 3179–3182. 10.1021/jp7115577.

[ref124] Li D.; Matanovic I.; Lee A. S.; Park E. J.; Fujimoto C.; Chung H. T.; Kim Y. S. Phenyl Oxidation Impacts the Durability of Alkaline Membrane Water Electrolyzer. ACS Appl. Mater. Interface 2019, 11, 9696–9701. 10.1021/acsami.9b00711. 30811171

[ref125] Matanovic I.; Kim Y. S. Electrochemical Phenyl Oxidation: A Limiting Factor of Oxygen Evolution Reaction in Water Electrolysis. Curr. Opin. Electrochem. 2023, 38, 101218 10.1016/j.coelec.2023.101218.

[ref126] Bauer B.; Strathmann H.; Effenberger F. Anion-Exchange Membranes with Improved Alkaline Stability. Desalination 1990, 79, 125–144. 10.1016/0011-9164(90)85002-R.

[ref127] Sturgeon M. R.; Macomber C. S.; Engtrakul C.; Long H.; Pivovar B. S. Hydroxide Based Benzyltrimethylammonium Degradation: Quantification of Rates and Degradation Technique Development. J. Electrochem. Soc. 2015, 162, F366–F372. 10.1149/2.0271504jes.

[ref128] Khalid H.; Najibah M.; Park H. S.; Bae C.; Henkensmeier D. Properties of Anion Exchange Membranes with a Focus on Water Electrolysis. Membranes 2022, 12, 989 10.3390/membranes12100989. 36295748 PMC9609780

[ref129] Einsla B. R.; Chempath S.; Pratt L.; Boncella J.; Rau J.; Macomber C.; Pivovar B. Stability of Cations for Anion Exchange Membrane Fuel Cells. ECS Trans. 2007, 11, 1173–1180. 10.1149/1.2781031.

[ref130] Marino M. G.; Kreuer K. D. Alkaline Stability of Quaternary Ammonium Cations for Alkaline Fuel Cell Membranes and Ionic Liquids. ChemSusChem 2015, 8, 513–523. 10.1002/cssc.201403022. 25431246

[ref131] Motealleh B.; Liu Z.; Masel R. I.; Sculley J. P.; Richard Ni Z.; Meroueh L. Next-Generation Anion Exchange Membrane Water Electrolyzers Operating for Commercially Relevant Lifetimes. Int. J. Hydrog. Energy 2021, 46, 3379–3386. 10.1016/j.ijhydene.2020.10.244.

[ref132] Kutz R. B.; Chen Q.; Yang H.; Sajjad S. D.; Liu Z.; Masel I. R. Sustainion Imidazolium-Functionalized Polymers for Carbon Dioxide Electrolysis. Energy Technol. 2017, 5, 929–936. 10.1002/ente.201600636.

[ref133] Hnát J.; Plevová M.; Žitka J.; Paidar M.; Bouzek K. Anion-Selective Materials with 1,4-Diazabicyclo[2.2.2]Octane Functional Groups for Advanced Alkaline Water Electrolysis. Electrochim. Acta 2017, 248, 547–555. 10.1016/j.electacta.2017.07.165.

[ref134] Hnát J.; Plevova M.; Tufa R. A.; Zitka J.; Paidar M.; Bouzek K. Development and Testing of a Novel Catalyst-Coated Membrane with Platinum-Free Catalysts for Alkaline Water Electrolysis. Int. J. Hydrog. Energy 2019, 44, 17493–17504. 10.1016/j.ijhydene.2019.05.054.

[ref135] Žitka J.; Peter J.; Galajdová B.; Pavlovec L.; Pientka Z.; Paidar M.; Hnát J.; Bouzek K. Anion Exchange Membranes and Binders Based on Polystyrene-Block-Poly(Ethylene-Ran-Butylene)-Block-Polystyrene Copolymer for Alkaline Water Electrolysis. Desalination Water Treat. 2019, 142, 90–97. 10.5004/dwt.2019.23411.

[ref136] Mandal M.; Huang G.; Kohl P. A. Highly Conductive Anion-Exchange Membranes Based on Cross-Linked Poly(Norbornene): Vinyl Addition Polymerization. ACS Appl. Energy Mater. 2019, 2, 2447–2457. 10.1021/acsaem.8b02051.

[ref137] Chen M.; Mandal M.; Groenhout K.; McCool G.; Tee H. M.; Zulevi B.; Kohl P. A. Self-Adhesive Ionomers for Durable Low-Temperature Anion Exchange Membrane Electrolysis. J. Power Sources 2022, 536, 231495 10.1016/j.jpowsour.2022.231495.

[ref138] Gaitor J. C.; Treichel M.; Kowalewski T.; Noonan K. J. T. Suppressing Water Uptake and Increasing Hydroxide Conductivity in Ring-Opened Polynorbornene Ion-Exchange Materials Via Backbone Design. ACS Appl. Polym. Mater. 2022, 4, 8032–8042. 10.1021/acsapm.2c00297.

[ref139] Lee W. H.; Park E. J.; Han J.; Shin D. W.; Kim Y. S.; Bae C. Poly(Terphenylene) Anion Exchange Membranes: The Effect of Backbone Structure on Morphology and Membrane Property. ACS Macro Lett. 2017, 6, 566–570. 10.1021/acsmacrolett.7b00148. 35610884

[ref140] Hu M.; Pearce E. M.; Kwei T. K. Modification of Polybenzimidazole: Synthesis and Thermal Stability of Poly(N1-methylbenzimidazole) and Poly(N1,N3-dimethylbenzimidazolium) Salt. J. Polym. Sci., Part A: Polym. Chem. 1993, 31, 553–561. 10.1002/pola.1993.080310228.

[ref141] Thomas O. D.; Soo K. J. W. Y.; Peckham T. J.; Kulkarni M. P.; Holdcroft S. Anion Conducting Poly(Dialkyl Benzimidazolium) Salts. Polym. Chem. 2011, 2, 1641 10.1039/c1py00142f.

[ref142] Henkensmeier D.; Kim H.-J.; Lee H.-J.; Lee D. H.; Oh I.-H.; Hong S.-A.; Nam S.-W.; Lim T.-H. Polybenzimidazolium-Based Solid Electrolytes. Macromol. Mater. Eng. 2011, 296, 899–908. 10.1002/mame.201100100.

[ref143] Henkensmeier D.; Cho H.-R.; Kim H.-J.; Nunes Kirchner C.; Leppin J.; Dyck A.; Jang J. H.; Cho E.; Nam S.-W.; Lim T.-H. Polybenzimidazolium Hydroxides - Structure, Stability and Degradation. Polym. Degrad. Stab. 2012, 97, 264–272. 10.1016/j.polymdegradstab.2011.12.024.

[ref144] Henkensmeier D.; Cho H.; Brela M.; Michalak A.; Dyck A.; Germer W.; Duong N. M. H.; Jang J. H.; Kim H.-J.; Woo N.-S.; Lim T.-H. Anion Conducting Polymers Based on Ether Linked Polybenzimidazole (PBI-OO). Int. J. Hydrog. Energy 2014, 39, 2842–2853. 10.1016/j.ijhydene.2013.07.091.

[ref145] Germer W.; Leppin J.; Nunes Kirchner C.; Cho H.; Kim H.-J.; Henkensmeier D.; Lee K.-Y.; Brela M.; Michalak A.; Dyck A. Phase Separated Methylated Polybenzimidazole (O-PBI) Based Anion Exchange Membranes. Macromol. Mater. Eng. 2015, 300, 497–509. 10.1002/mame.201400345.

[ref146] Wright A. G.; Holdcroft S. Hydroxide-Stable Ionenes. ACS Macro Lett. 2014, 3, 444–447. 10.1021/mz500168d. 35590779

[ref147] Wright A. G.; Fan J.; Britton B.; Weissbach T.; Lee H.-F.; Kitching E. A.; Peckham T. J.; Holdcroft S. Hexamethyl-P-Terphenyl Poly(Benzimidazolium): A Universal Hydroxide-Conducting Polymer for Energy Conversion Devices. Energy Environ. Sci. 2016, 9, 2130–2142. 10.1039/C6EE00656F.

[ref148] Weissbach T.; Wright A. G.; Peckham T. J.; Sadeghi Alavijeh A.; Pan V.; Kjeang E.; Holdcroft S. Simultaneous, Synergistic Control of Ion Exchange Capacity and Cross-Linking of Sterically-Protected Poly(Benzimidazolium)S. Chem. Mater. 2016, 28, 8060–8070. 10.1021/acs.chemmater.6b03902.

[ref149] Hou H.; Wang S.; Liu H.; Sun L.; Jin W.; Jing M.; Jiang L.; Sun G. Synthesis and Characterization of a New Anion Exchange Membrane by a Green and Facile Route. Int. J. Hydrog. Energy 2011, 36, 11955–11960. 10.1016/j.ijhydene.2011.06.054.

[ref150] Fan J.; Willdorf-Cohen S.; Schibli E. M.; Paula Z.; Li W.; Skalski T. J. G.; Sergeenko A. T.; Hohenadel A.; Frisken B. J.; Magliocca E.; Mustain W. E.; Diesendruck C. E.; Dekel D. R.; Holdcroft S. Poly(Bis-Arylimidazoliums) Possessing High Hydroxide Ion Exchange Capacity and High Alkaline Stability. Nat. Commun. 2019, 10, 2306 10.1038/s41467-019-10292-z. 31127108 PMC6534565

[ref151] Moreno-González M.; Mardle P.; Zhu S.; Gholamkhass B.; Jones S.; Chen N.; Britton B.; Holdcroft S. One Year Operation of an Anion Exchange Membrane Water Electrolyzer Utilizing Aemion+® Membrane: Minimal Degradation, Low H_2_ Crossover and High Efficiency. J. Power Sources Adv. 2023, 19, 100109 10.1016/j.powera.2023.100109.

[ref152] Long H.; Pivovar B. Hydroxide Degradation Pathways for Imidazolium Cations: A DFT Study. J. Phys. Chem. C 2014, 118, 9880–9888. 10.1021/jp501362y.

[ref153] Hugar K. M.; Kostalik H. A. t.; Coates G. W. Imidazolium Cations with Exceptional Alkaline Stability: A Systematic Study of Structure-Stability Relationships. J. Am. Chem. Soc. 2015, 137, 8730–8737. 10.1021/jacs.5b02879. 26062959

[ref154] Choi S. Y.; Ikhsan M. M.; Jin K. S.; Henkensmeier D. Nanostructure-Property Relationship of Two Perfluorinated Sulfonic Acid (PFSA) Membranes. Int. J. Energy Res. 2022, 46, 11265–11277. 10.1002/er.7926.

[ref155] Schibli E. M.; Stewart J. C.; Wright A. A.; Chen B.; Holdcroft S.; Frisken B. J. The Nanostructure of HMT-PMBI, a Sterically Hindered Ionene. Macromolecules 2020, 53, 4908–4916. 10.1021/acs.macromol.0c00978.

[ref156] Cao X.; Novitski D.; Holdcroft S. Visualization of Hydroxide Ion Formation Upon Electrolytic Water Splitting in an Anion Exchange Membrane. ACS Mater. Lett. 2019, 1, 362–366. 10.1021/acsmaterialslett.9b00195.

[ref157] Wang L.; Weissbach T.; Reissner R.; Ansar A.; Gago A. S.; Holdcroft S.; Friedrich K. A. High Performance Anion Exchange Membrane Electrolysis Using Plasma-Sprayed, Non-Precious-Metal Electrodes. ACS Appl. Energy Mater. 2019, 2, 7903–7912. 10.1021/acsaem.9b01392.

[ref158] Fortin P.; Khoza T.; Cao X.; Martinsen S. Y.; Oyarce Barnett A.; Holdcroft S. High-Performance Alkaline Water Electrolysis Using Aemion Anion Exchange Membranes. J. Power Sources 2020, 451, 227814 10.1016/j.jpowsour.2020.227814.

[ref159] Khataee A.; Shirole A.; Jannasch P.; Krüger A.; Cornell A. Anion Exchange Membrane Water Electrolysis Using Aemion Membranes and Nickel Electrodes. J. Mater. Chem. A 2022, 10, 16061–16070. 10.1039/D2TA03291K.

[ref160] Wei Q.; Cao X.; Veh P.; Konovalova A.; Mardle P.; Overton P.; Cassegrain S.; Vierrath S.; Breitwieser M.; Holdcroft S. On the Stability of Anion Exchange Membrane Fuel Cells Incorporating Polyimidazolium Ionene (Aemion+®) Membranes and Ionomers. Sustain. Energy Fuels 2022, 6, 3551–3564. 10.1039/D2SE00690A.

[ref161] Couture G.; Alaaeddine A.; Boschet F.; Ameduri B. Polymeric Materials as Anion-Exchange Membranes for Alkaline Fuel Cells. Prog. Polym. Sci. 2011, 36, 1521–1557. 10.1016/j.progpolymsci.2011.04.004.

[ref162] Chen N.; Lee Y. M. Anion Exchange Polyelectrolytes for Membranes and Ionomers. Prog. Polym. Sci. 2021, 113, 101345 10.1016/j.progpolymsci.2020.101345.

[ref163] You W.; Noonan K. J. T.; Coates G. W. Alkaline-Stable Anion Exchange Membranes: A Review of Synthetic Approaches. Prog. Polym. Sci. 2020, 100, 101177 10.1016/j.progpolymsci.2019.101177.

[ref164] Arges C. G.; Wang L.; Parrondo J.; Ramani V. Best Practices for Investigating Anion Exchange Membrane Suitability for Alkaline Electrochemical Devices: Case Study Using Quaternary Ammonium Poly(2,6-Dimethyl 1,4-Phenylene)Oxide Anion Exchange Membranes. J. Electrochem. Soc. 2013, 160, F1258–F1274. 10.1149/2.049311jes.

[ref165] Parrondo J.; Jung M.-s. J.; Wang Z.; Arges C. G.; Ramani V. Synthesis and Alkaline Stability of Solubilized Anion Exchange Membrane Binders Based on Poly(Phenylene Oxide) Functionalized with Quaternary Ammonium Groups Via a Hexyl Spacer. J. Electrochem. Soc. 2015, 162, F1236–F1242. 10.1149/2.0891510jes.

[ref166] Fujimoto C.; Kim D.-S.; Hibbs M.; Wrobleski D.; Kim Y. S. Backbone Stability of Quaternized Polyaromatics for Alkaline Membrane Fuel Cells. J. Membr. Sci. 2012, 423–424, 438–449. 10.1016/j.memsci.2012.08.045.

[ref167] Miyanishi S.; Yamaguchi T. Ether Cleavage-Triggered Degradation of Benzyl Alkylammonium Cations for Polyethersulfone Anion Exchange Membranes. Phys. Chem. Chem. Phys. 2016, 18, 12009–12023. 10.1039/C6CP00579A. 27066832

[ref168] Mohanty A. D.; Tignor S. E.; Krause J. A.; Choe Y.-K.; Bae C. Systematic Alkaline Stability Study of Polymer Backbones for Anion Exchange Membrane Applications. Macromolecules 2016, 49, 3361–3372. 10.1021/acs.macromol.5b02550.

[ref169] Lee W. H.; Kim Y. S.; Bae C. Robust Hydroxide Ion Conducting Poly(Biphenyl Alkylene)s for Alkaline Fuel Cell Membranes. ACS Macro Lett. 2015, 4, 814–818. 10.1021/acsmacrolett.5b00375. 35596501

[ref170] Noh S.; Jeon J. Y.; Adhikari S.; Kim Y. S.; Bae C. Molecular Engineering of Hydroxide Conducting Polymers for Anion Exchange Membranes in Electrochemical Energy Conversion Technology. Acc. Chem. Res. 2019, 52, 2745–2755. 10.1021/acs.accounts.9b00355. 31454229

[ref171] Olah G. A. Superelectrophiles. Angew. Chem., Int. Ed. Engl. 1993, 32, 767–788. 10.1002/anie.199307673.

[ref172] Olah G. A.; Surya Prakash G. K.; Molnár Á.; Sommer J. Superacid Chemistry, 2nd ed.; John Wiley & Sons: Hoboken, NJ, 2009.

[ref173] Colquhoun H. M.; Zolotukhin M. G.; Khalilov L. M.; Dzhemilev U. M. Superelectrophiles in Aromatic Polymer Chemistry. Macromolecules 2001, 34, 1122–1124. 10.1021/ma001579o.

[ref174] Zolotukhin M. G.; Fomina L.; Salcedo R.; Sansores L. E.; Colquhoun H. M.; Khalilov L. M. Superelectrophiles in Polymer Chemistry. A Novel, One-Pot Synthesis of High-Tg, High-Temperature Polymers. Macromolecules 2004, 37, 5140–5141. 10.1021/ma0495902.

[ref175] Zolotukhin M. G.; Fomine S.; Lazo L. M.; Salcedo R.; Sansores L. E.; Cedillo G. G.; Colquhoun H. M.; Fernandez-G J. M.; Khalizov A. F. Superacid-Catalyzed Polycondensation of Acenaphthenequinone with Aromatic Hydrocarbons. Macromolecules 2005, 38, 6005–6014. 10.1021/ma0503460.

[ref176] Diaz A. M.; Zolotukhin M. G.; Fomine S.; Salcedo R.; Manero O.; Cedillo G.; Velasco V. M.; Guzman M. T.; Fritsch D.; Khalizov A. F. A Novel, One-Pot Synthesis of Novel 3f, 5f, and 8f Aromatic Polymers. Macromol. Rapid Commun. 2007, 28, 183–187. 10.1002/marc.200600656.

[ref177] Zolotukhin M. G.; Fomine S.; Lazo L. M.; Hernández M. D. C. G.; Guzmán-Gutiérrez M. T.; Ruiz-Trevino A.; Fritsch D.; Cuellas D. C.; Fernandez-G J. M. A Novel Approach to the Synthesis of High Performance and Functional Polymers. High Perform. Polym. 2007, 19, 638–648. 10.1177/0954008307081204.

[ref178] Cruz A. R.; Zolotukhin M. G.; Morales S. L.; Cardenas J.; Cedillo G.; Fomine S.; Salmon M.; Carreon-Castro M. P. Use of 4-Piperidones in One-Pot Syntheses of Novel, High-Molecular-Weight Linear and Virtually 100%-Hyperbranched Polymers. Chem. Commun. 2009, 4408–4410. 10.1039/b907042g. 19597608

[ref179] Hernandez M. C. G.; Zolotukhin M. G.; Fomine S.; Cedillo G.; Morales S. L.; Fröhlich N.; Preis E.; Scherf U.; Salmón M.; Chávez M. I.; Cárdenas J.; Ruiz-Trevino A. Novel, Metal-Free, Superacid-Catalyzed “Click” Reactions of Isatins with Linear, Nonactivated, Multiring Aromatic Hydrocarbons. Macromolecules 2010, 43, 6968–6979. 10.1021/ma101048z.

[ref180] Guzmán-Gutiérrez M. T.; Nieto D. R.; Fomine S.; Morales S. L.; Zolotukhin M. G.; Hernandez M. C. G.; Kricheldorf H.; Wilks E. S. Dramatic Enhancement of Superacid-Catalyzed Polyhydroxyalkylation Reactions. Macromolecules 2011, 44, 194–202. 10.1021/ma102267f.

[ref181] Kricheldorf H. R.; Zolotukhin M. G.; Cardenas J. Non-Stoichiometric Polycondensations and the Synthesis of High Molar Mass Polycondensates. Macromol. Rapid Commun. 2012, 33, 1814–1832. 10.1002/marc.201200345. 23002001

[ref182] Cruz A. R.; Hernandez M. C. G.; Guzmán-Gutiérrez M. T.; Zolotukhin M. G.; Fomine S.; Morales S. L.; Kricheldorf H.; Wilks E. S.; Cárdenas J.; Salmón M. Precision Synthesis of Narrow Polydispersity, Ultrahigh Molecular Weight Linear Aromatic Polymers by A2 + B2 Nonstoichiometric Step-Selective Polymerization. Macromolecules 2012, 45, 6774–6780. 10.1021/ma301691f.

[ref183] Olvera L. I.; Guzmán-Gutiérrez M. T.; Zolotukhin M. G.; Fomine S.; Cárdenas J.; Ruiz-Trevino F. A.; Villers D.; Ezquerra T. A.; Prokhorov E. Novel High Molecular Weight Aromatic Fluorinated Polymers from One-Pot, Metal-Free Step Polymerizations. Macromolecules 2013, 46, 7245–7256. 10.1021/ma401306s.

[ref184] González-Díaz M. O.; Cetina-Mancilla E.; Sulub-Sulub R.; Montes-Luna A.; Olvera L. I.; Zolotukhin M. G.; Cárdenas J.; Aguilar-Vega M. Novel Fluorinated Aromatic Polymers with Ether-Bond-Free Aryl Backbones for Pure and Mixed Gas Separation. J. Membr. Sci. 2020, 606, 118114 10.1016/j.memsci.2020.118114.

[ref185] Cruz-Rosado A.; Romero-Hernández J. E.; Ríos-López M.; López-Morales S.; Cedillo G.; Ríos-Ruiz L. M.; Cetina-Mancilla E.; Palacios-Alquisira J.; Zolotukhin M. G.; Vivaldo-Lima E. Molecular Weight Development in the Superacid-Catalyzed Polyhydroxyalkylation of 1-Propylisatin and Biphenyl at Stoichiometric Conditions. Polymer 2022, 243, 124616 10.1016/j.polymer.2022.124616.

[ref186] Cetina-Mancilla E.; Reyes-García G. A.; Rodríguez-Molina M.; Zolotukhin M. G.; Vivaldo-Lima E.; Ortencia González-Díaz M.; Ramos-Ortiz G. Room Temperature, Simple and Efficient Synthesis and Functionalization of Aromatic Poly(Arylene Sulfide)s, Poly(Arylene Sulfoxide)s and Poly(Arylene Sulfone)s. Eur. Polym. J. 2023, 184, 111800 10.1016/j.eurpolymj.2022.111800.

[ref187] Klumpp D. A.; Garza M.; Jones A.; Mendoza S. Synthesis of Aryl-Substituted Piperidines by Superacid Activation of Piperidones. J. Org. Chem. 1999, 64, 6702–6705. 10.1021/jo990454i. 11674674

[ref188] Olsson J. S.; Pham T. H.; Jannasch P. Poly(Arylene Piperidinium) Hydroxide Ion Exchange Membranes: Synthesis, Alkaline Stability, and Conductivity. Adv. Funct. Mater. 2018, 28, 1702758 10.1002/adfm.201702758.

[ref189] Wang J.; Zhao Y.; Setzler B. P.; Rojas-Carbonell S.; Ben Yehuda C.; Amel A.; Page M.; Wang L.; Hu K.; Shi L.; Gottesfeld S.; Xu B.; Yan Y. Poly(Aryl Piperidinium) Membranes and Ionomers for Hydroxide Exchange Membrane Fuel Cells. Nat. Energy 2019, 4, 392–398. 10.1038/s41560-019-0372-8.

[ref190] Peng H.; Li Q.; Hu M.; Xiao L.; Lu J.; Zhuang L. Alkaline Polymer Electrolyte Fuel Cells Stably Working at 80 °C. J. Power Sources 2018, 390, 165–167. 10.1016/j.jpowsour.2018.04.047.

[ref191] Chen N.; Wang H. H.; Kim S. P.; Kim H. M.; Lee W. H.; Hu C.; Bae J. Y.; Sim E. S.; Chung Y. C.; Jang J. H.; Yoo S. J.; Zhuang Y.; Lee Y. M. Poly(Fluorenyl Aryl Piperidinium) Membranes and Ionomers for Anion Exchange Membrane Fuel Cells. Nat. Commun. 2021, 12, 2367 10.1038/s41467-021-22612-3. 33888709 PMC8062622

[ref192] Chen N.; Paek S. Y.; Lee J. Y.; Park J. H.; Lee S. Y.; Lee Y. M. High-Performance Anion Exchange Membrane Water Electrolyzers with a Current Density of 7.68 A cm^–2^ and a Durability of 1000 h. Energy Environ. Sci. 2021, 14, 6338–6348. 10.1039/D1EE02642A.

[ref193] Chen N.; Hu C.; Wang H. H.; Kim S. P.; Kim H. M.; Lee W. H.; Bae J. Y.; Park J. H.; Lee Y. M. Poly(Alkyl-Terphenyl Piperidinium) Ionomers and Membranes with an Outstanding Alkaline-Membrane Fuel-Cell Performance of 2.58 W cm^–2^. Angew. Chem., Int. Ed. Engl. 2021, 60, 7710–7718. 10.1002/anie.202013395. 33368927 PMC8048807

[ref194] Hu C.; Park J. H.; Kim H. M.; Wang H. H.; Bae J. Y.; Liu M.-L.; Kang N. Y.; Yoon K.-s.; Park C.-d.; Chen N.; Lee Y. M. Robust and Durable Poly(Aryl-Co-Aryl Piperidinium) Reinforced Membranes for Alkaline Membrane Fuel Cells. J. Mater. Chem. A 2022, 10, 6587–6595. 10.1039/D2TA00196A.

[ref195] Wang H. H.; Hu C.; Park J. H.; Kim H. M.; Kang N. Y.; Bae J. Y.; Lee W. H.; Chen N.; Lee Y. M. Reinforced Poly(Fluorenyl-Co-Terphenyl Piperidinium) Anion Exchange Membranes for Fuel Cells. J. Membr. Sci. 2022, 644, 120160 10.1016/j.memsci.2021.120160.

[ref196] Long C.; Wang Z.; Zhu H. High Chemical Stability Anion Exchange Membrane Based on Poly(Aryl Piperidinium): Effect of Monomer Configuration on Membrane Properties. Int. J. Hydrog. Energy 2021, 46, 18524–18533. 10.1016/j.ijhydene.2021.02.209.

[ref197] Mayadevi T. S.; Sung S.; Varghese L.; Kim T. H. Poly(m*eta*/p*ara*-terphenylene-methyl piperidinium)-Based Anion Exchange Membranes: The Effect of Backbone Structure in AEMFC Application. Membranes 2020, 10, 329 10.3390/membranes10110329. 33167367 PMC7694387

[ref198] Yan X.; Yang X.; Su X.; Gao L.; Zhao J.; Hu L.; Di M.; Li T.; Ruan X.; He G. Twisted Ether-Free Polymer Based Alkaline Membrane for High-Performance Water Electrolysis. J. Power Sources 2020, 480, 228805 10.1016/j.jpowsour.2020.228805.

[ref199] Caielli T.; Ferrari A. R.; Bonizzoni S.; Sediva E.; Caprì A.; Santoro M.; Gatto I.; Baglio V.; Mustarelli P. Synthesis, Characterization and Water Electrolyzer Cell Tests of Poly(Biphenyl Piperidinium) Anion Exchange Membranes. J. Power Sources 2023, 557, 232532 10.1016/j.jpowsour.2022.232532.

[ref200] Xiao J.; Oliveira A. M.; Wang L.; Zhao Y.; Wang T.; Wang J.; Setzler B. P.; Yan Y. Water-Fed Hydroxide Exchange Membrane Electrolyzer Enabled by a Fluoride-Incorporated Nickel-Iron Oxyhydroxide Oxygen Evolution Electrode. ACS Catal. 2021, 11, 264–270. 10.1021/acscatal.0c04200.

[ref201] Jeon Y.; Tinh V. D. C.; Thuc V. D.; Kim D. Ether-Free Polymer Based Bipolar Electrolyte Membranes without an Interlayer Catalyst for Water Electrolysis with Durability at a High Current Density. Chem. Eng. J. 2023, 459, 141467 10.1016/j.cej.2023.141467.

[ref202] Liu M.; Hu X.; Hu B.; Liu L.; Li N. Soluble Poly(Aryl Piperidinium) with Extended Aromatic Segments as Anion Exchange Membranes for Alkaline Fuel Cells and Water Electrolysis. J. Membr. Sci. 2022, 642, 119966 10.1016/j.memsci.2021.119966.

[ref203] Yang Y.; Jiang T.; Li L.; Zhou S.; Fang H.; Li X.; Wei H.; Ding Y. Chemo-Stable Poly(Quinquephenylene-Co-Diphenylene Piperidinium) Ionomers for Anion Exchange Membrane Fuel Cells. J. Power Sources 2021, 506, 230184 10.1016/j.jpowsour.2021.230184.

[ref204] Hu C.; Park J. H.; Kim H. M.; Wang H. H.; Bae J. Y.; Kang N. Y.; Chen N.; Lee Y. M. Elucidating the Role of Alkyl Chain in Poly(Aryl Piperidinium) Copolymers for Anion Exchange Membrane Fuel Cells. J. Membr. Sci. 2022, 647, 120341 10.1016/j.memsci.2022.120341.

[ref205] Wei C.; Yu W.; Zhang Y.; Zhang F.; Li M.; Shen X.; Zhang K.; Ge X.; Wu L.; Xu T. Alkaline Anion Exchange Membrane Containing Pyrene-Based π-π Stacking Interactions. J. Power Sources 2023, 553, 232247 10.1016/j.jpowsour.2022.232247.

[ref206] Gao W. T.; Gao X. L.; Gou W. W.; Wang J. J.; Cai Z. H.; Zhang Q. G.; Zhu A. M.; Liu Q. L. High-Performance Tetracyclic Aromatic Anion Exchange Membranes Containing Twisted Binaphthyl for Fuel Cells. J. Membr. Sci. 2022, 655, 120578 10.1016/j.memsci.2022.120578.

[ref207] Wang X.; Qiao X.; Liu S.; Liu L.; Li N. Poly(Terphenyl Piperidinium) Containing Hydrophilic Crown Ether Units in Main Chains as Anion Exchange Membranes for Alkaline Fuel Cells and Water Electrolyzers. J. Membr. Sci. 2022, 653, 120558 10.1016/j.memsci.2022.120558.

[ref208] Liu J.; Gao L.; Di M.; Hu L.; Sun X.; Wu X.; Jiang X.; Dai Y.; Yan X.; He G. Low Boiling Point Solvent-Soluble, Highly Conductive and Stable Poly (Ether Phenylene Piperidinium) Anion Exchange Membrane. J. Membr. Sci. 2022, 644, 120185 10.1016/j.memsci.2021.120185.

[ref209] Yuan W.; Zeng L.; Jiang S.; Yuan C.; He Q.; Wang J.; Liao Q.; Wei Z. High Performance Poly(Carbazolyl Aryl Piperidinium) Anion Exchange Membranes for Alkaline Fuel Cells. J. Membr. Sci. 2022, 657, 120676 10.1016/j.memsci.2022.120676.

[ref210] Wang Y.; Wang Y.; Guo M.; Ban T.; Zhu X. Poly(Isatin-Piperidinium-Terphenyl) Anion Exchange Membranes with Improved Performance for Direct Borohydride Fuel Cells. Int. J. Hydrog. Energy 2023, 48, 14837–14852. 10.1016/j.ijhydene.2022.12.082.

[ref211] Hu X.; Huang Y.; Liu L.; Ju Q.; Zhou X.; Qiao X.; Zheng Z.; Li N. Piperidinium Functionalized Aryl Ether-Free Polyaromatics as Anion Exchange Membrane for Water Electrolyzers: Performance and Durability. J. Membr. Sci. 2021, 621, 118964 10.1016/j.memsci.2020.118964.

[ref212] Pham T. H.; Olsson J. S.; Jannasch P. Poly(Arylene Alkylene)S with Pendant N-Spirocyclic Quaternary Ammonium Cations for Anion Exchange Membranes. J. Mater. Chem. A 2018, 6, 16537–16547. 10.1039/C8TA04699A.

[ref213] Zhu H.; Li Y.; Chen N.; Lu C.; Long C.; Li Z.; Liu Q. Controllable Physical-Crosslinking Poly(Arylene 6-Azaspiro[5.5] Undecanium) for Long-Lifetime Anion Exchange Membrane Applications. J. Membr. Sci. 2019, 590, 117307 10.1016/j.memsci.2019.117307.

[ref214] Wang F.; Li Y.; Li C.; Zhu H. Preparation and Study of Spirocyclic Cationic Side Chain Functionalized Polybiphenyl Piperidine Anion Exchange Membrane. J. Membr. Sci. 2021, 620, 118919 10.1016/j.memsci.2020.118919.

[ref215] Wang X.; Lin C.; Gao Y.; Lammertink R. G. H. Anion Exchange Membranes with Twisted Poly(Terphenylene) Backbone: Effect of the N-Cyclic Cations. J. Membr. Sci. 2021, 635, 119525 10.1016/j.memsci.2021.119525.

[ref216] Liu L.; Bai L.; Liu Z.; Miao S.; Pan J.; Shen L.; Shi Y.; Li N. Side-Chain Structural Engineering on Poly(Terphenyl Piperidinium) Anion Exchange Membrane for Water Electrolyzers. J. Membr. Sci. 2023, 665, 121135 10.1016/j.memsci.2022.121135.

[ref217] Du X.; Zhang H.; Yuan Y.; Wang Z. Constructing Micro-Phase Separation Structure to Improve the Performance of Anion-Exchange Membrane Based on Poly(Aryl Piperidinium) Cross-Linked Membranes. J. Power Sources 2021, 487, 229429 10.1016/j.jpowsour.2020.229429.

[ref218] Du X.; Wang Z.; Zhang H.; Yuan Y.; Wang H.; Zhang Z. Prepared Poly(Aryl Piperidinium) Anion Exchange Membranes for Acid Recovery to Improve Dialysis Coefficients and Selectivity. J. Membr. Sci. 2021, 619, 118805 10.1016/j.memsci.2020.118805.

[ref219] Liu Q.; Ma W.; Tian L.; Li J.; Yang L.; Wang F.; Wang Z.; Li J.; Wang Z.; Zhu H. Side-Chain Cation-Grafted Poly(Biphenyl Piperidine) Membranes for Anion Exchange Membrane Fuel Cells. J. Power Sources 2022, 551, 232105 10.1016/j.jpowsour.2022.232105.

[ref220] Che X.; Tang W.; Dong J.; Aili D.; Yang J. Anion Exchange Membranes Based on Long Side-Chain Quaternary Ammonium-Functionalized Poly(Arylene Piperidinium)S for Vanadium Redox Flow Batteries. Sci. China Mater. 2022, 65, 683–694. 10.1007/s40843-021-1786-0.

[ref221] Kim H. M.; Hu C.; Wang H. H.; Park J. H.; Chen N.; Lee Y. M. Impact of Side-Chains in Poly(Dibenzyl-Co-Terphenyl Piperidinium) Copolymers for Anion Exchange Membrane Fuel Cells. J. Membr. Sci. 2022, 644, 120109 10.1016/j.memsci.2021.120109.

[ref222] Liu B.; Duan Y.; Li T.; Li J.; Zhang H.; Zhao C. Nanostructured Anion Exchange Membranes Based on Poly(Arylene Piperidinium) with Bis-Cation Strings for Diffusion Dialysis in Acid Recovery. Sep. Sci. Technol. 2022, 282, 120032 10.1016/j.seppur.2021.120032.

[ref223] Xu L.; Wang H.; Min L.; Xu W.; Wang Y.; Zhang W. Anion Exchange Membranes Based on Poly(Aryl Piperidinium) Containing Both Hydrophilic and Hydrophobic Side Chains. Ind. Eng. Chem. Res. 2022, 61, 14232–14241. 10.1021/acs.iecr.2c01722.

[ref224] Pan D.; Pham T. H.; Jannasch P. Poly(Arylene Piperidine) Anion Exchange Membranes with Tunable N-Alicyclic Quaternary Ammonium Side Chains. ACS Appl. Energy Mater. 2021, 4, 11652–11665. 10.1021/acsaem.1c02389.

[ref225] Ma L.; Hussain M.; Li L.; Qaisrani N. A.; Bai L.; Jia Y.; Yan X.; Zhang F.; He G. Octopus-Like Side Chain Grafted Poly(Arylene Piperidinium) Membranes for Fuel Cell Application. J. Membr. Sci. 2021, 636, 119529 10.1016/j.memsci.2021.119529.

[ref226] Jin Y.; Che X.; Xu Y.; Dong J.; Pan C.; Aili D.; Li Q.; Yang J. An Imidazolium Type Ionic Liquid Functionalized Ether-Free Poly(Terphenyl Piperidinium) Membrane for High Temperature Polymer Electrolyte Membrane Fuel Cell Applications. J. Electrochem. Soc. 2022, 169, 024504 10.1149/1945-7111/ac4d6d.

[ref227] Jin C.; Zhang S.; Cong Y.; Zhu X. Highly Durable and Conductive Poly(Arylene Piperidine) with a Long Heterocyclic Ammonium Side-Chain for Hydroxide Exchange Membranes. Int. J. Hydrog. Energy 2019, 44, 24954–24964. 10.1016/j.ijhydene.2019.07.184.

[ref228] Zhao B.; Zhang Z.; Zhang J.; Wang L.; Wang T.; Dong J.; Xu C.; Yang J. Long Side-Chain N-Heterocyclic Cation Based Ionic Liquid Grafted Poly(Terphenyl Piperidinium) Membranes for Anion Exchange Membrane Fuel Cell Applications. Polymer 2023, 286, 126404 10.1016/j.polymer.2023.126404.

[ref229] Lu C.; Long C.; Li Y.; Li Z.; Zhu H. Chemically Stable Poly(Meta-Terphenyl Piperidinium) with Highly Conductive Side Chain for Alkaline Fuel Cell Membranes. J. Membr. Sci. 2020, 598, 117797 10.1016/j.memsci.2019.117797.

[ref230] Gou W. W.; Gao W. T.; Gao X. L.; Zhang Q. G.; Zhu A. M.; Liu Q. L. Highly Conductive Fluorinated Poly(Biphenyl Piperidinium) Anion Exchange Membranes with Robust Durability. J. Membr. Sci. 2022, 645, 120200 10.1016/j.memsci.2021.120200.

[ref231] Lin C.; Cheng W.; Miao X.; Shen X.; Ling L. Clustered Piperidinium-Functionalized Poly(Terphenylene) Anion Exchange Membranes with Well-Developed Conductive Nanochannels. J. Colloid Interface Sci. 2022, 608, 1247–1256. 10.1016/j.jcis.2021.10.122. 34739988

[ref232] Zhang H.; Li Z. a.; Hu L.; Gao L.; Di M.; Du Y.; Yan X.; Dai Y.; Ruan X.; He G. Covalent/Ionic Co-Crosslinking Constructing Ultra-Densely Functionalized Ether-Free Poly(Biphenylene Piperidinium) Amphoteric Membranes for Vanadium Redox Flow Batteries. Electrochim. Acta 2020, 359, 136879 10.1016/j.electacta.2020.136879.

[ref233] Yan X.; Zhang H.; Hu Z.; Li L.; Hu L.; Li Z.; Gao L.; Dai Y.; Jian X.; He G. Amphoteric-Side-Chain-Functionalized ″Ether-Free″ Poly(Arylene Piperidinium) Membrane for Advanced Redox Flow Battery. ACS Appl. Mater. Interface 2019, 11, 44315–44324. 10.1021/acsami.9b15872. 31670931

[ref234] Yang L.; Wang Z.; Wang F.; Wang Z.; Zhu H. Poly(Aryl Piperidinium) Anion Exchange Membranes with Cationic Extender Sidechain for Fuel Cells. J. Membr. Sci. 2022, 653, 120448 10.1016/j.memsci.2022.120448.

[ref235] Zhang J.; Zhang K.; Liang X.; Yu W.; Ge X.; Shehzad M. A.; Ge Z.; Yang Z.; Wu L.; Xu T. Self-Aggregating Cationic-Chains Enable Alkaline Stable Ion-Conducting Channels for Anion-Exchange Membrane Fuel Cells. J. Mater. Chem. A 2021, 9, 327–337. 10.1039/D0TA11011F.

[ref236] Tang W.; Mu T.; Che X.; Dong J.; Yang J. Highly Selective Anion Exchange Membrane Based on Quaternized Poly(Triphenyl Piperidine) for the Vanadium Redox Flow Battery. ACS Sustain. Chem. Eng. 2021, 9, 14297–14306. 10.1021/acssuschemeng.1c05648.

[ref237] Fan Y.; Zhou J.; Chen J.; Shen C.; Gao S. Polyaryl Piperidine Anion Exchange Membranes with Hydrophilic Side Chain. Int. J. Hydrog. Energy 2023, 48, 17630–17640. 10.1016/j.ijhydene.2023.01.243.

[ref238] Hu C.; Park J. H.; Kang N. Y.; Zhang X.; Lee Y. J.; Jeong S. W.; Lee Y. M. Effects of Hydrophobic Side Chains in Poly(Fluorenyl-Co-Aryl Piperidinium) Ionomers for Durable Anion Exchange Membrane Fuel Cells. J. Mater. Chem. A 2023, 11, 2031–2041. 10.1039/D2TA08726J.

[ref239] Xu F.; Chen Y.; Cao X.; Li J.; Lin B.; Yuan N.; Ding J. Comb-Shaped Polyfluorene with Variable Alkyl Chain Length for Application as Anion Exchange Membranes. J. Power Sources 2022, 545, 231880 10.1016/j.jpowsour.2022.231880.

[ref240] Zhou X.; Wu L.; Zhang G.; Li R.; Hu X.; Chang X.; Shen Y.; Liu L.; Li N. Rational Design of Comb-Shaped Poly(Arylene Indole Piperidinium) to Enhance Hydroxide Ion Transport for H_2_/O_2_ Fuel Cell. J. Membr. Sci. 2021, 631, 119335 10.1016/j.memsci.2021.119335.

[ref241] Cai Z. H.; Gao X. L.; Gao W. T.; Choo Y. S. L.; Wang J. J.; Zhang Q. G.; Zhu A. M.; Liu Q. L. Effect of Hydrophobic Side Chain Length on Poly(Carbazolyl Terphenyl Piperidinium) Anion Exchange Membranes. ACS Appl. Energy Mater. 2022, 5, 10165–10176. 10.1021/acsaem.2c01908.

[ref242] Li R.; Chen X.; Zhou X.; Shen Y.; Fu Y. Understanding of Hydroxide Transport in Poly(Arylene Indole Piperidinium) Anion Exchange Membranes: Effect of Side-Chain Position. Sep. Purification Technol. 2023, 314, 123577 10.1016/j.seppur.2023.123577.

[ref243] Adamski M.; Skalski T. J. G.; Schibli E. M.; Killer M.; Wu Y.; Peressin N.; Frisken B. J.; Holdcroft S. Molecular Branching as a Simple Approach to Improving Polymer Electrolyte Membranes. J. Membr. Sci. 2020, 595, 117539 10.1016/j.memsci.2019.117539.

[ref244] Wu X.; Chen N.; Klok H. A.; Lee Y. M.; Hu X. Branched Poly(Aryl Piperidinium) Membranes for Anion-Exchange Membrane Fuel Cells. Angew. Chem., Int. Ed. Engl. 2022, 61, e202114892 10.1002/anie.202114892. 34904347 PMC9304273

[ref245] Bai L.; Ma L.; Li L.; Zhang A.; Yan X.; Zhang F.; He G. Branched, Side-Chain Grafted Polyarylpiperidine Anion Exchange Membranes for Fuel Cell Application. ACS Appl. Energy Mater. 2021, 4, 6957–6967. 10.1021/acsaem.1c01037.

[ref246] Liu Q.; Zhang S.; Tian L.; Li J.; Ma W.; Wang F.; Wang Z.; Li J.; Zhu H. “Windmill” Shaped Branched Anion-Conducting Poly(Aryl Piperidine) with Extra Molecular Interaction Sites as New Anion Exchange Membranes. J. Power Sources 2023, 564, 232822 10.1016/j.jpowsour.2023.232822.

[ref247] Liu X.; Wei W.; Yang Y.; Li Y.; Li Y.; Xu S.; Dong Y.; He R. A Porous Membrane Electrolyte Enabled by Poly(Biphenyl Piperidinium Triphenylmethane) for Dendrite-Free Zinc Anode with Enhanced Cycling Life. Chem. Eng. J. 2022, 437, 135409 10.1016/j.cej.2022.135409.

[ref248] Li X.; Zhang B.; Guo J.; Chen Y.; Dai L.; Zheng J.; Li S.; Zhang S. High-Strength, Ultra-Thin Anion Exchange Membranes with a Branched Structure toward Alkaline Membrane Fuel Cells. J. Mater. Chem. A 2023, 11, 10738–10747. 10.1039/D2TA09914D.

[ref249] Olsson J. S.; Pham T. H.; Jannasch P. Tuning Poly(Arylene Piperidinium) Anion-Exchange Membranes by Copolymerization, Partial Quaternization and Crosslinking. J. Membr. Sci. 2019, 578, 183–195. 10.1016/j.memsci.2019.01.036.

[ref250] Tian L.; Li J.; Liu Q.; Ma W.; Wang F.; Zhu H.; Wang Z. Cross-Linked Anion-Exchange Membranes with Dipole-Containing Cross-Linkers Based on Poly(Terphenyl Isatin Piperidinium) Copolymers. ACS Appl. Mater. Interface 2022, 14, 39343–39353. 10.1021/acsami.2c08221. 35997247

[ref251] Wang X.; Lammertink R. G. H. Dimensionally Stable Multication-Crosslinked Poly(Arylene Piperidinium) Membranes for Water Electrolysis. J. Mater. Chem. A 2022, 10, 8401–8412. 10.1039/D1TA10820D.

[ref252] Chen N.; Park J. H.; Hu C.; Wang H. H.; Kim H. M.; Kang N. Y.; Lee Y. M. Di-Piperidinium-Crosslinked Poly(Fluorenyl-Co-Terphenyl Piperidinium)S for High-Performance Alkaline Exchange Membrane Fuel Cells. J. Mater. Chem. A 2022, 10, 3678–3687. 10.1039/D1TA10178A.

[ref253] Chen N.; Lu C.; Li Y.; Long C.; Li Z.; Zhu H. Tunable Multi-Cations-Crosslinked Poly(Arylene Piperidinium)-Based Alkaline Membranes with High Ion Conductivity and Durability. J. Membr. Sci. 2019, 588, 117120 10.1016/j.memsci.2019.05.044.

[ref254] Jia Y.; Ma L.; Yu Q.; Qaisrani N. A.; Li L.; Zhou R.; He G.; Zhang F. Partially Fluorinated, Multication Cross-Linked Poly(Arylene Piperidinium) Membranes with Improved Conductivity and Reduced Swelling for Fuel Cell Application. Ionics 2020, 26, 5617–5627. 10.1007/s11581-020-03721-3.

[ref255] Wang J. J.; Gao W. T.; Choo Y. S. L.; Cai Z. H.; Zhang Q. G.; Zhu A. M.; Liu Q. L. Highly Conductive Branched Poly(Aryl Piperidinium) Anion Exchange Membranes with Robust Chemical Stability. J. Colloid Interface Sci. 2023, 629, 377–387. 10.1016/j.jcis.2022.08.183. 36087553

[ref256] Chen N.; Hu C.; Wang H. H.; Park J. H.; Kim H. M.; Lee Y. M. Chemically & Physically Stable Crosslinked Poly(Aryl-Co-Aryl Piperidinium)S for Anion Exchange Membrane Fuel Cells. J. Membr. Sci. 2021, 638, 119685 10.1016/j.memsci.2021.119685.

[ref257] Song W.; Peng K.; Xu W.; Liu X.; Zhang H.; Liang X.; Ye B.; Zhang H.; Yang Z.; Wu L.; Ge X.; Xu T. Upscaled Production of an Ultramicroporous Anion-Exchange Membrane Enables Long-Term Operation in Electrochemical Energy Devices. Nat. Commun. 2023, 14, 2732 10.1038/s41467-023-38350-7. 37169752 PMC10175247

[ref258] Liu B.; Li T.; Li Q.; Zhu S.; Duan Y.; Li J.; Zhang H.; Zhao C. Enhanced Diffusion Dialysis Performance of Cross-Linked Poly(Aryl Piperidine) Anion Exchange Membranes by Thiol-Ene Click Chemistry for Acid Recovery. J. Membr. Sci. 2022, 660, 120816 10.1016/j.memsci.2022.120816.

[ref259] Tinh V. D. C.; Thuc V. D.; Jeon Y.; Gu G.-Y.; Kim D. Highly Durable Poly(Arylene Piperidinium) Composite Membranes Modified with Polyhedral Oligomeric Silsesquioxane for Fuel Cell and Water Electrolysis Application. J. Membr. Sci. 2022, 660, 120903 10.1016/j.memsci.2022.120903.

[ref260] Xu F.; Chen Y.; Li J.; Lin B.; Chu F.; Ding J. Polyfluorene/Poly(Vinylbenzyl Chloride) Cross-Linked Anion-Exchange Membranes with Multiple Cations for Fuel Cell Applications. ACS Appl. Energy Mater. 2022, 5, 9101–9108. 10.1021/acsaem.2c01592.

[ref261] Chen N.; Lu C.; Li Y.; Long C.; Zhu H. Robust Poly(Aryl Piperidinium)/N-Spirocyclic Poly(2,6-Dimethyl-1,4-Phenyl) for Hydroxide-Exchange Membranes. J. Membr. Sci. 2019, 572, 246–254. 10.1016/j.memsci.2018.10.067.

[ref262] Wang F.; Li C.; Sang J.; Li J. Poly(Terphenylene) and Bromohexylated Polystyrene-B-Poly(Ethylene-Co-Butene)-B-Polystyrene Cross-Linked Anion Exchange Membrane with Excellent Comprehensive Performance. Energy Fuels 2022, 36, 7795–7805. 10.1021/acs.energyfuels.2c01670.

[ref263] Min K.; Chae J. E.; Lee Y.; Kim H.-J.; Kim T.-H. Crosslinked Poly(m-terphenyl N-methyl piperidinium)-SEBS Membranes with Aryl-Ether Free and Kinked Backbones as Highly Stable and Conductive Anion Exchange Membranes. J. Membr. Sci. 2022, 653, 120487 10.1016/j.memsci.2022.120487.

[ref264] Min K.; Lee Y.; Choi Y.; Kwon O. J.; Kim T.-H. High-Performance Anion Exchange Membranes Achieved by Crosslinking Two Aryl Ether-Free Polymers: Poly(bibenzyl N-methyl piperidine) and SEBS. J. Membr. Sci. 2022, 664, 121071 10.1016/j.memsci.2022.121071.

[ref265] Wang L.; You R.; Ling Y.; Xie Y.; Mei C.; Li Z.; Wang F. Covalent Cross-Linked Anion Exchange Membrane Based on Poly(Biphenyl Piperidine) and Poly(Styrene-B-(Ethylene-Co-Butylene)-B-Styrene): Preparation and Properties. Polym.-Plast. Technol. Mater. 2021, 60, 1233–1246. 10.1080/25740881.2021.1888987.

[ref266] Park E. J.; Capuano C. B.; Ayers K. E.; Bae C. Chemically Durable Polymer Electrolytes for Solid-State Alkaline Water Electrolysis. J. Power Sources 2018, 375, 367–372. 10.1016/j.jpowsour.2017.07.090.

[ref267] Park E. J.; Maurya S.; Lee A. S.; Leonard D. P.; Li D.; Jeon J. Y.; Bae C.; Kim Y. S. How Does a Small Structural Change of Anode Ionomer Make a Big Difference in Alkaline Membrane Fuel Cell Performance?. J. Mater. Chem. A 2019, 7, 25040–25046. 10.1039/C9TA10157H.

[ref268] Wang T.; Jeon J. Y.; Han J.; Kim J. H.; Bae C.; Kim S. Poly(Terphenylene) Anion Exchange Membranes with High Conductivity and Low Vanadium Permeability for Vanadium Redox Flow Batteries (VRFBs). J. Membr. Sci. 2020, 598, 117665 10.1016/j.memsci.2019.117665.

[ref269] Jiang T.; Wu C.; Zhou Y.; Cheng S.; Yang S.; Wei H.; Ding Y.; Wu Y. Highly Stable Poly(p-Quaterphenylene Alkylene)-Based Anion Exchange Membranes. J. Membr. Sci. 2022, 647, 120342 10.1016/j.memsci.2022.120342.

[ref270] Jiang T.; Zhou Y.; Yang Y.; Wu C.; Fang H.; Yang S.; Wei H.; Ding Y. Dimensionally and Oxidatively Stable Anion Exchange Membranes Based on Bication Cross-Linked Poly(Meta-Terphenylene Alkylene)s. Polymer 2021, 216, 123433 10.1016/j.polymer.2021.123433.

[ref271] Li L.; Jiang T.; Wang S.; Cheng S.; Li X.; Wei H.; Ding Y. Branched Anion-Conducting Poly(Arylene Alkylene)S for Alkaline Membrane Fuel Cells. ACS Appl. Energy Mater. 2022, 5, 2462–2473. 10.1021/acsaem.1c03952.

[ref272] Wang T.; Zhao Y.; Wang S.; Cheng S.; Yang S.; Wei H.; Ding Y. Towards High Alkaline Stability and Fuel Cell Performance in Anion Exchange Membranes Via Backbone-Cation Alkylene Spacer Tuning for Quaternized Poly(Biphenylene Alkylene)S. J. Power Sources 2023, 557, 232590 10.1016/j.jpowsour.2022.232590.

[ref273] Jiang T.; Wang C.; Wang T.; Wang X.; Wang X.; Li X.; Ding Y.; Wei H. Imidazolium Structural Isomer Pyrazolium: A Better Alkali-Stable Anion Conductor for Anion Exchange Membranes. J. Membr. Sci. 2022, 660, 120843 10.1016/j.memsci.2022.120843.

[ref274] Ren R.; Zhang S.; Miller H. A.; Vizza F.; Varcoe J. R.; He Q. Facile Preparation of an Ether-Free Anion Exchange Membrane with Pendant Cyclic Quaternary Ammonium Groups. ACS Appl. Energy Mater. 2019, 2, 4576–4581. 10.1021/acsaem.9b00674.

[ref275] Allushi A.; Pham T. H.; Jannasch P. Highly Conductive Hydroxide Exchange Membranes Containing Fluorene-Units Tethered with Dual Pairs of Quaternary Piperidinium Cations. J. Membr. Sci. 2021, 632, 119376 10.1016/j.memsci.2021.119376.

[ref276] Allushi A.; Bakvand P. M.; Jannasch P. Polyfluorenes Bearing N,N-Dimethylpiperidinium Cations on Short Spacers for Durable Anion Exchange Membranes. Macromolecules 2023, 56, 1165–1176. 10.1021/acs.macromol.2c02291.

[ref277] Allushi A.; Pham T. H.; Olsson J. S.; Jannasch P. Ether-Free Polyfluorenes Tethered with Quinuclidinium Cations as Hydroxide Exchange Membranes. J. Mater. Chem. A 2019, 7, 27164–27174. 10.1039/C9TA09213G.

[ref278] Li X.; Yang K.; Li Z.; Guo J.; Zheng J.; Li S.; Zhang S.; Sherazi T. A. The Effect of Side Chain Length on the Morphology and Transport Properties of Fluorene-Based Anion Exchange Membranes. Int. J. Hydrog. Energy 2022, 47, 15044–15055. 10.1016/j.ijhydene.2022.03.009.

[ref279] Li X.; Yang K.; Wang Z.; Chen Y.; Li Y.; Guo J.; Zheng J.; Li S.; Zhang S. Chain Architecture Dependence of Morphology and Water Transport in Poly(Fluorene Alkylene)-Based Anion-Exchange Membranes. Macromolecules 2022, 55, 10607–10617. 10.1021/acs.macromol.2c01488.

[ref280] Mohamed Ahmed Mahmoud A.; Miyatake K. Highly Conductive and Ultra Alkaline Stable Anion Exchange Membranes by Superacid-Promoted Polycondensation for Fuel Cells. ACS Appl. Polym. Mater. 2023, 5, 2243–2253. 10.1021/acsapm.2c02227.

[ref281] Adhikari S.; Leonard D. P.; Lim K. H.; Park E. J.; Fujimoto C.; Morales-Collazo O.; Brennecke J. F.; Hu Z.; Jia H.; Kim Y. S. Hydrophobic Quaternized Poly(Fluorene) Ionomers for Emerging Fuel Cells. ACS Appl. Energy Mater. 2022, 5, 2663–2668. 10.1021/acsaem.2c00119.

[ref282] Wu X.; Chen N.; Hu C.; Klok H. A.; Lee Y. M.; Hu X. Fluorinated Poly(Aryl Piperidinium) Membranes for Anion Exchange Membrane Fuel Cells. Adv. Mater. 2023, 35, e2210432 10.1002/adma.202210432. 36642967

[ref283] Wei C.; Yu W.; Wu L.; Ge X.; Xu T. Physically and Chemically Stable Anion Exchange Membranes with Hydrogen-Bond Induced Ion Conducting Channels. Polymers 2022, 14, 4920 10.3390/polym14224920. 36433047 PMC9696997

[ref284] Cha M. S.; Park J. E.; Kim S.; Han S.-H.; Shin S.-H.; Yang S. H.; Kim T.-H.; Yu D. M.; So S.; Hong Y. T.; Yoon S. J.; Oh S.-G.; Kang S. Y.; Kim O.-H.; Park H. S.; Bae B.; Sung Y.-E.; Cho Y.-H.; Lee J. Y. Poly(Carbazole)-Based Anion-Conducting Materials with High Performance and Durability for Energy Conversion Devices. Energy Environ. Sci. 2020, 13, 3633–3645. 10.1039/D0EE01842B.

[ref285] Li J.; Liu Q.; Tian L.; Ma W.; Wang F.; Wang Z.; Zhu H. Novel Poly(Carbazole-Butanedione) Anion Exchange Membranes Constructed by Obvious Microphase Separation for Fuel Cells. Int. J. Hydrog. Energy 2022, 47, 32262–32272. 10.1016/j.ijhydene.2022.07.095.

[ref286] Ren R.; Zhang S.; Miller H. A.; Vizza F.; Varcoe J. R.; He Q. Facile Preparation of Novel Cardo Poly(Oxindolebiphenylylene) with Pendent Quaternary Ammonium by Superacid-Catalysed Polyhydroxyalkylation Reaction for Anion Exchange Membranes. J. Membr. Sci. 2019, 591, 117320 10.1016/j.memsci.2019.117320.

[ref287] Wang K.; Gao L.; Liu J.; Su X.; Yan X.; Dai Y.; Jiang X.; Wu X.; He G. Comb-Shaped Ether-Free Poly(Biphenyl Indole) Based Alkaline Membrane. J. Membr. Sci. 2019, 588, 117216 10.1016/j.memsci.2019.117216.

[ref288] Zhang S.; Zhu X.; Jin C. Development of a High-Performance Anion Exchange Membrane Using Poly(Isatin Biphenylene) with Flexible Heterocyclic Quaternary Ammonium Cations for Alkaline Fuel Cells. Journal of Materials Chemistry A 2019, 7, 6883–6893. 10.1039/C8TA11291F.

[ref289] Long C.; Zhao T.; Tian L.; Liu Q.; Wang F.; Wang Z.; Zhu H. Highly Stable and Conductive Multicationic Poly(Biphenyl Indole) with Extender Side Chains for Anion Exchange Membrane Fuel Cells. ACS Appl. Energy Mater. 2021, 4, 6154–6165. 10.1021/acsaem.1c00942.

[ref290] Zhang J.; Yu W.; Liang X.; Zhang K.; Wang H.; Ge X.; Wei C.; Song W.; Ge Z.; Wu L.; Xu T. Flexible Bis-Piperidinium Side Chains Construct Highly Conductive and Robust Anion-Exchange Membranes. ACS Appl. Energy Mater. 2021, 4, 9701–9711. 10.1021/acsaem.1c01783.

[ref291] Zhao T.; Long C.; Wang Z.; Zhu H. Multication Cross-Linked Poly(P-Terphenyl Isatin) Anion Exchange Membranes for Fuel Cells: Effect of Cross-Linker Length on Membrane Performance. ACS Appl. Energy Mater. 2021, 4, 14476–14487. 10.1021/acsaem.1c03153.

[ref292] Yuan Y.; Du X.; Zhang H.; Wang H.; Wang Z. Poly (Isatin Biphenylene) Polymer Containing Ferrocenium Derivatives for Anion Exchange Membrane Fuel Cell. J. Membr. Sci. 2022, 642, 119986 10.1016/j.memsci.2021.119986.

[ref293] Ma L.; Li L.; Yuan M.; Bai L.; Zhang A.; Yan X.; He G.; Zhang F. Hydrophilic-Hydrophobic Bulky Units Modified Anion Exchange Membranes for Fuel Cell Application. ACS Sustain. Chem. Eng. 2022, 10, 5748–5757. 10.1021/acssuschemeng.1c07886.

[ref294] Li Z.; Guo J.; Zheng J.; Sherazi T. A.; Li S.; Zhang S. A Microporous Polymer with Suspended Cations for Anion Exchange Membrane Fuel Cells. Macromolecules 2020, 53, 10998–11008. 10.1021/acs.macromol.0c01948.

[ref295] Wang Q.; Huang L.; Wang Z.; Zheng J.; Zhang Q.; Qin G.; Li S.; Zhang S. High Conductive Anion Exchange Membranes from All-Carbon Twisted Intrinsic Microporous Polymers. Macromolecules 2022, 55, 10713–10722. 10.1021/acs.macromol.2c01874.

[ref296] Xu S.; Wei W.; Su X.; He R. Crown-Ether Block Copolymer Based Poly(Isatin Terphenyl) Anion Exchange Membranes for Electrochemical Energy Conversion Devices. Chem. Eng. J. 2023, 455, 140776 10.1016/j.cej.2022.140776.

[ref297] Zhu L.; Pan J.; Wang Y.; Han J.; Zhuang L.; Hickner M. A. Multication Side Chain Anion Exchange Membranes. Macromolecules 2016, 49, 815–824. 10.1021/acs.macromol.5b02671.

[ref298] Dang H.-S.; Jannasch P. Exploring Different Cationic Alkyl Side Chain Designs for Enhanced Alkaline Stability and Hydroxide Ion Conductivity of Anion-Exchange Membranes. Macromolecules 2015, 48, 5742–5751. 10.1021/acs.macromol.5b01302.

[ref299] Narducci R.; Becerra-Arciniegas R. A.; Pasquini L.; Ercolani G.; Knauth P.; Di Vona M. L. Anion-Conducting Polymer Electrolyte without Ether Linkages and with Ionic Groups Grafted on Long Side Chains: Poly(Alkylene Biphenyl Butyltrimethyl Ammonium) (ABBA). Membranes 2022, 12, 337 10.3390/membranes12030337. 35323811 PMC8956100

[ref300] Pan D.; Chen S.; Jannasch P. Alkali-Stable Anion Exchange Membranes Based on Poly(Xanthene). ACS Macro Lett. 2023, 12, 20–25. 10.1021/acsmacrolett.2c00672. 36538018 PMC9850910

[ref301] Mansouri Bakvand P.; Jannasch P. Poly(Arylene Alkylene)s with Pendent Benzyl-Tethered Ammonium Cations for Anion Exchange Membranes. J. Membr. Sci. 2023, 668, 121229 10.1016/j.memsci.2022.121229.

[ref302] Zhang S.; Wang Y.; Gao X.; Liu P.; Wang X.; Zhu X. Enhanced Conductivity and Stability Via Comb-Shaped Polymer Anion Exchange Membrane Incorporated with Porous Polymeric Nanospheres. J. Membr. Sci. 2020, 597, 117750 10.1016/j.memsci.2019.117750.

[ref303] Shin D. W.; Guiver M. D.; Lee Y. M. Hydrocarbon-Based Polymer Electrolyte Membranes: Importance of Morphology on Ion Transport and Membrane Stability. Chem. Rev. 2017, 117, 4759–4805. 10.1021/acs.chemrev.6b00586. 28257183

[ref304] Pham T. H.; Olsson J. S.; Jannasch P. Effects of the N-Alicyclic Cation and Backbone Structures on the Performance of Poly(Terphenyl)-Based Hydroxide Exchange Membranes. J. Mater. Chem. A 2019, 7, 15895–15906. 10.1039/C9TA05531B.

[ref305] Xue B.; Cui W.; Zhou S.; Zhang Q.; Zheng J.; Li S.; Zhang S. Facile Preparation of Highly Alkaline Stable Poly(Arylene-Imidazolium) Anion Exchange Membranes through an Ionized Monomer Strategy. Macromolecules 2021, 54, 2202–2212. 10.1021/acs.macromol.0c02612.

[ref306] Wang T.; Dong J.; Yu N.; Tang W.; Jin Y.; Xu Y.; Yang J. Synthesis and Properties of a New Ether-Free Poly(Bis-Alkylpiperidinium) Polymer for the Anion Exchange Membrane. Eur. Polym. J. 2022, 173, 111271 10.1016/j.eurpolymj.2022.111271.

[ref307] Su X.; Gao L.; Hu L.; Qaisrani N. A.; Yan X.; Zhang W.; Jiang X.; Ruan X.; He G. Novel Piperidinium Functionalized Anionic Membrane for Alkaline Polymer Electrolysis with Excellent Electrochemical Properties. J. Membr. Sci. 2019, 581, 283–292. 10.1016/j.memsci.2019.03.072.

[ref308] Li H.; Kraglund M. R.; Reumert A. K.; Ren X.; Aili D.; Yang J. Poly(Vinyl Benzyl Methylpyrrolidinium) Hydroxide Derived Anion Exchange Membranes for Water Electrolysis. J. Mater. Chem. A 2019, 7, 17914–17922. 10.1039/C9TA04868E.

[ref309] Li H.; Yu N.; Gellrich F.; Reumert A. K.; Kraglund M. R.; Dong J.; Aili D.; Yang J. Diamine Crosslinked Anion Exchange Membranes Based on Poly(Vinyl Benzyl Methylpyrrolidinium) for Alkaline Water Electrolysis. J. Membr. Sci. 2021, 633, 119418 10.1016/j.memsci.2021.119418.

[ref310] Olsson J. S.; Pham T. H.; Jannasch P. Functionalizing Polystyrene with N-Alicyclic Piperidine-Based Cations Via Friedel-Crafts Alkylation for Highly Alkali-Stable Anion-Exchange Membranes. Macromolecules 2020, 53, 4722–4732. 10.1021/acs.macromol.0c00201. 32905320 PMC7467773

[ref311] Jeon J. Y.; Park S.; Han J.; Maurya S.; Mohanty A. D.; Tian D.; Saikia N.; Hickner M. A.; Ryu C. Y.; Tuckerman M. E.; Paddison S. J.; Kim Y. S.; Bae C. Synthesis of Aromatic Anion Exchange Membranes by Friedel-Crafts Bromoalkylation and Cross-Linking of Polystyrene Block Copolymers. Macromolecules 2019, 52, 2139–2147. 10.1021/acs.macromol.8b02355.

[ref312] Jeon J. Y.; Tian D.; Pagels M. K.; Bae C. Efficient Preparation of Styrene Block Copolymer Anion Exchange Membranes Via One-Step Friedel-Crafts Bromoalkylation with Alkenes. Org. Process Res. Dev. 2019, 23, 1580–1586. 10.1021/acs.oprd.9b00218.

[ref313] Ponce-González J.; Ouachan I.; Varcoe J. R.; Whelligan D. K. Radiation-Induced Grafting of a Butyl-Spacer Styrenic Monomer onto Etfe: The Synthesis of the Most Alkali Stable Radiation-Grafted Anion-Exchange Membrane to Date. J. Mater. Chem. A 2018, 6, 823–827. 10.1039/C7TA10222D.

[ref314] Meek K. M.; Reed C. M.; Pivovar B.; Kreuer K. D.; Varcoe J. R.; Bance-Soualhi R. The Alkali Degradation of LDPE-Based Radiation-Grafted Anion-Exchange Membranes Studied Using Different Ex Situ Methods. RSC Adv. 2020, 10, 36467–36477. 10.1039/D0RA06484J. 35517956 PMC9056956

[ref315] Gupta G.; Scott K.; Mamlouk M. Performance of Polyethylene Based Radiation Grafted Anion Exchange Membrane with Polystyrene-b-Poly (Ethylene/Butylene)-b-Polystyrene Based Ionomer Using NiCo_2_O_4_ Catalyst for Water Electrolysis. J. Power Sources 2018, 375, 387–396. 10.1016/j.jpowsour.2017.07.026.

[ref316] Li D.; Motz A. R.; Bae C.; Fujimoto C.; Yang G.; Zhang F.-Y.; Ayers K. E.; Kim Y. S. Durability of Anion Exchange Membrane Water Electrolyzers. Energy Environ. Sci. 2021, 14, 3393–3419. 10.1039/D0EE04086J.

[ref317] Mohanty A. D.; Ryu C. Y.; Kim Y. S.; Bae C. Stable Elastomeric Anion Exchange Membranes Based on Quaternary Ammonium-Tethered Polystyrene-B-Poly(Ethylene-Co-Butylene)-B-Polystyrene Triblock Copolymers. Macromolecules 2015, 48, 7085–7095. 10.1021/acs.macromol.5b01382.

[ref318] Shi Y.; Zhao Z.; Liu W.; Zhang C. Physically Self-Cross-Linked SEBS Anion Exchange Membranes. Energy Fuels 2020, 34, 16746–16755. 10.1021/acs.energyfuels.0c03017.

[ref319] Liu Z.; Sajjad S. D.; Gao Y.; Yang H.; Kaczur J. J.; Masel R. I. The Effect of Membrane on an Alkaline Water Electrolyzer. Int. J. Hydrog. Energy 2017, 42, 29661–29665. 10.1016/j.ijhydene.2017.10.050.

[ref320] Lee J.; Jung H.; Park Y. S.; Woo S.; Kwon N.; Xing Y.; Oh S. H.; Choi S. M.; Han J. W.; Lim B. Corrosion-Engineered Bimetallic Oxide Electrode as Anode for High-Efficiency Anion Exchange Membrane Water Electrolyzer. Chem. Eng. J. 2021, 420, 127670 10.1016/j.cej.2020.127670.

[ref321] Xu Z.; Wilke V.; Chmielarz J. J.; Tobias M.; Atanasov V.; Gago A. S.; Friedrich K. A. Novel Piperidinium-Functionalized Crosslinked Anion Exchange Membrane with Flexible Spacers for Water Electrolysis. J. Membr. Sci. 2023, 670, 121302 10.1016/j.memsci.2022.121302.

[ref322] Najibah M.; Kong J.; Khalid H.; Hnát J.; Park H. S.; Bouzek K.; Henkensmeier D. Pre-Swelling of FAA3Membranes with Water-Based Ethylene Glycol Solution to Minimize Dimensional Changes after Assembly into a Water Electrolyzer: Effect on Properties and Performance. J. Membr. Sci. 2023, 670, 121344 10.1016/j.memsci.2022.121344.

[ref323] Zhang H.; Ohashi H.; Tamaki T.; Yamaguchi T. Water Movement in a Solid-State Alkaline Fuel Cell Affected by the Anion-Exchange Pore-Filling Membrane Properties. J. Phys. Chem. C 2013, 117, 16791–16801. 10.1021/jp405088s.

[ref324] Rakhshani S.; Araneo R.; Pucci A.; Rinaldi A.; Giuliani C.; Pozio A. Synthesis and Characterization of a Composite Anion Exchange Membrane for Water Electrolyzers (AEMWE). Membranes 2023, 13, 109 10.3390/membranes13010109. 36676916 PMC9860756

[ref325] Park J.; Wang L.; Advani S. G.; Prasad A. K. Durability Analysis of Nafion/Hydrophilic Pretreated PTFE Membranes for Pemfcs. J. Electrochem. Soc. 2012, 159, F864–F870. 10.1149/2.016301jes.

[ref326] Li D.; Xu K.; Zhang Y. A Review on Research Progress in Plasma-Controlled Superwetting Surface Structure and Properties. Polymers 2022, 14, 3759 10.3390/polym14183759. 36145911 PMC9505013

[ref327] Jones D.; Roziere J.; Cavaliere S.; Subianto S.; Burton S. (Johnson Matthey Fuel Cells Limited, Centre national de la recherche scientifique, Universite Montpellier) Patent WO 2016/020668 Al, 2014.

[ref328] Abouzari-Lotf E.; Jacob M. V.; Ghassemi H.; Zakeri M.; Nasef M. M.; Abdolahi Y.; Abbasi A.; Ahmad A. Highly Conductive Anion Exchange Membranes Based on Polymer Networks Containing Imidazolium Functionalised Side Chains. Sci. Rep. 2021, 11, 3764 10.1038/s41598-021-83161-9. 33580110 PMC7881124

[ref329] Ahmed Mahmoud A. M.; Miyatake K. Tuning the Hydrophobic Component in Reinforced Poly(Arylimidazolium)-Based Anion Exchange Membranes for Alkaline Fuel Cells. ACS Appl. Energy Mater. 2022, 5, 15211–15221. 10.1021/acsaem.2c02868.

[ref330] Wang Z.; Parrondo J.; Sankarasubramanian S.; Bhattacharyya K.; Ghosh M.; Ramani V. Alkaline Stability of Pure Aliphatic-Based Anion Exchange Membranes Containing Cycloaliphatic Quaternary Ammonium Cations. J. Electrochem. Soc. 2020, 167, 124504 10.1149/1945-7111/abac29.

[ref331] Nauman S.; Lubineau G.; Alharbi H. F. Post Processing Strategies for the Enhancement of Mechanical Properties of Enms (Electrospun Nanofibrous Membranes): A Review. Membranes 2021, 11, 39 10.3390/membranes11010039. 33466446 PMC7824849

[ref332] Mun S. C.; Won J. H. Manufacturing Processes of Microporous Polyolefin Separators for Lithium-Ion Batteries and Correlations between Mechanical and Physical Properties. Crystals 2021, 11, 1013 10.3390/cryst11091013.

[ref333] Washburn E. W. The Dynamics of Capillary Flow. Phys. Rev. 1921, 17, 273–283. 10.1103/PhysRev.17.273.

[ref334] Li K.; Zhang D.; Bian H.; Meng C.; Yang Y. Criteria for Applying the Lucas-Washburn Law. Sci. Rep. 2015, 5, 14085 10.1038/srep14085. 26364749 PMC4568521

[ref335] Chatenet M.; Pollet B. G.; Dekel D. R.; Dionigi F.; Deseure J.; Millet P.; Braatz R. D.; Bazant M. Z.; Eikerling M.; Staffell I.; Balcombe P.; Shao-Horn Y.; Schafer H. Water Electrolysis: From Textbook Knowledge to the Latest Scientific Strategies and Industrial Developments. Chem. Soc. Rev. 2022, 51, 4583–4762. 10.1039/D0CS01079K. 35575644 PMC9332215

[ref336] In Lee H.; Dung D. T.; Kim J.; Pak J. H.; Kim S. k.; Cho H. S.; Cho W. C.; Kim C. H. The Synthesis of a Zirfon-Type Porous Separator with Reduced Gas Crossover for Alkaline Electrolyzer. Int. J. Energy Res. 2020, 44, 1875–1885. 10.1002/er.5038.

[ref337] https://www.agfa.com/specialty-products/wp-content/uploads/sites/8/2020/10/TDS-ZIRFON-UTP-500_20200831.pdf (accessed 2023-08-03).

[ref338] Yuan X.; Yan T.; Liu Z.; Kang P. Highly Efficient Alkaline Water Electrolysis Using Alkanolamine-Functionalized Zirconia-Blended Separators. ACS Sustain. Chem. Eng. 2023, 11, 4269–4278. 10.1021/acssuschemeng.2c07618.

[ref339] Vermeiren P.; Adriansens W.; Moreels J. P.; Leysen R. In Hydrogen Power: Theoretical and Engineering Solutions; Springer Science + Business Media: Dordrecht, 1998.

[ref340] Rodriguez J.; Palmas S.; Sanchez-Molina M.; Amores E.; Mais L.; Campana R. Simple and Precise Approach for Determination of Ohmic Contribution of Diaphragms in Alkaline Water Electrolysis. Membranes 2019, 9, 129 10.3390/membranes9100129. 31590320 PMC6835761

[ref341] Meek K. M.; Antunes C. M.; Strasser D.; Owczarczyk Z. R.; Neyerlin A.; Pivovar B. S. High-Throughput Anion Exchange Membrane Characterization at Nrel. ECS Trans. 2019, 92, 723–731. 10.1149/09208.0723ecst.

[ref342] Yanagi H.; Fukuta K. Anion Exchange Membrane and Ionomer for Alkaline Membrane Fuel Cells (Amfcs). ECS Trans. 2008, 16, 257–262. 10.1149/1.2981860.

[ref343] Ziv N.; Dekel D. R. A Practical Method for Measuring the True Hydroxide Conductivity of Anion Exchange Membranes. Electrochem. Commun. 2018, 88, 109–113. 10.1016/j.elecom.2018.01.021.

[ref344] Zhegur-Khais A.; Kubannek F.; Krewer U.; Dekel D. R. Measuring the True Hydroxide Conductivity of Anion Exchange Membranes. J. Membr. Sci. 2020, 612, 118461 10.1016/j.memsci.2020.118461.

[ref345] Luo X.; Rojas-Carbonell S.; Yan Y.; Kusoglu A. Structure-Transport Relationships of Poly(Aryl Piperidinium) Anion-Exchange Membranes: Eeffect of Anions and Hydration. J. Membr. Sci. 2020, 598, 117680 10.1016/j.memsci.2019.117680.

[ref346] Trinke P.; Haug P.; Brauns J.; Bensmann B.; Hanke-Rauschenbach R.; Turek T. Hydrogen Crossover in PEM and Alkaline Water Electrolysis: Mechanisms, Direct Comparison and Mitigation Strategies. J. Electrochem. Soc. 2018, 165, F502–F513. 10.1149/2.0541807jes.

[ref347] https://orbit.dtu.dk/files/117260640/RES_Hydrogen.pdf (accessed 2024-01-08).

[ref348] Schalenbach M. Hydrogen Diffusivity and Electrolyte Permeability of the Zirfon Perl Separator for Alkaline Water Electrolysis (Vol 163, Pg F1480, 2016). J. Electrochem. Soc. 2017, 164, X23–X23. 10.1149/2.0051802jes.

[ref349] Aili D.; Kraglund M. R.; Rajappan S. C.; Serhiichuk D.; Xia Y.; Deimede V.; Kallitsis J.; Bae C.; Jannasch P.; Henkensmeier D.; Jensen J. O. Electrode Separators for the Next-Generation Alkaline Water Electrolyzers. ACS Energy Lett. 2023, 8, 1900–1910. 10.1021/acsenergylett.3c00185. 37090167 PMC10111418

[ref350] Tanis-Kanbur M. B.; Peinador R. I.; Calvo J. I.; Hernández A.; Chew J. W. Porosimetric Membrane Characterization Techniques: A Review. J. Membr. Sci. 2021, 619, 118750 10.1016/j.memsci.2020.118750.

[ref351] https://www.irena.org/-/media/Files/IRENA/Agency/Publication/2020/Dec/IRENA_Green_hydrogen_cost_2020.pdf (accessed 2023-08-08).

[ref352] Xu L.; Yu Y.; Li W.; You Y.; Xu W.; Zhang S. X. The Influence of Manufacturing Parameters and Adding Support Layer on the Properties of Zirfon (R) Separators. Front. Chem. Sci. Eng. 2014, 8, 295–305. 10.1007/s11705-014-1433-y.

[ref353] Chen X.; Qian G.; Molleo M. A.; Benicewicz B. C.; Ploehn H. J. High Temperature Creep Behavior of Phosphoric Acid-Polybenzimidazole Gel Membranes. J. Polym. Sci., Part B: Polym. Phys. 2015, 53, 1527–1538. 10.1002/polb.23791.

[ref354] Molleo M. A.; Chen X.; Ploehn H. J.; Fishel K. J.; Benicewicz B. C. High Polymer Content 3,5-Pyridine-Polybenzimidazole Copolymer Membranes with Improved Compressive Properties. Fuel Cells 2014, 14, 16–25. 10.1002/fuce.201300202.

[ref355] Jheng L.-C.; Tai C.-K.; Hsu S. L.-C.; Lin B.-Y.; Chen L.; Wang B.-C.; Chiang L.-K.; Ko W.-C. Study on the Alkaline Stability of Imidazolium and Benzimidazolium Based Polyelectrolytes for Anion Exchange Membrane Fuel Cells. Int. J. Hydrog. Energy 2017, 42, 5315–5326. 10.1016/j.ijhydene.2016.11.129.

[ref356] Wu Y.; Wu C.; Xu T.; Lin X.; Fu Y. Novel Silica/Poly(2,6-Dimethyl-1,4-Phenylene Oxide) Hybrid Anion-Exchange Membranes for Alkaline Fuel Cells: Effect of Heat Treatment. J. Membr. Sci. 2009, 338, 51–60. 10.1016/j.memsci.2009.04.012.

[ref357] Haj-Bsoul S.; Varcoe J. R.; Dekel D. R. Measuring the Alkaline Stability of Anion-Exchange Membranes. J. Electroanal. Chem. 2022, 908, 116112 10.1016/j.jelechem.2022.116112.

[ref358] Zhang Y.; Parrondo J.; Sankarasubramanian S.; Ramani V. Detection of Reactive Oxygen Species in Anion Exchange Membrane Fuel Cells Using in Situ Fluorescence Spectroscopy. ChemSusChem 2017, 10, 3056–3062. 10.1002/cssc.201700760. 28657214

[ref359] Parrondo J.; Wang Z.; Jung M. S.; Ramani V. Reactive Oxygen Species Accelerate Degradation of Anion Exchange Membranes Based on Polyphenylene Oxide in Alkaline Environments. Phys. Chem. Chem. Phys. 2016, 18, 19705–19712. 10.1039/C6CP01978A. 27381009

[ref360] Ye N.; Xu Y.; Zhang D.; Yang J.; He R. Inhibition Mechanism of the Radical Inhibitors to Alkaline Degradation of Anion Exchange Membranes. Polym. Degrad. Stab. 2018, 153, 298–306. 10.1016/j.polymdegradstab.2018.05.002.

[ref361] Alghunaim A.; Kirdponpattara S.; Newby B. M. Z. Techniques for Determining Contact Angle and Wettability of Powders. Powder Technol. 2016, 287, 201–215. 10.1016/j.powtec.2015.10.002.

[ref362] Bhavanari M.; Lee K.-R.; Tseng C.-J.; Tang I. H.; Chen H.-H. Cufe Electrocatalyst for Hydrogen Evolution Reaction in Alkaline Electrolysis. Int. J. Hydrog. Energy 2021, 46, 35886–35895. 10.1016/j.ijhydene.2021.01.227.

[ref363] Holzapfel P.; Bühler M.; Van Pham C.; Hegge F.; Böhm T.; McLaughlin D.; Breitwieser M.; Thiele S. Directly Coated Membrane Electrode Assemblies for Proton Exchange Membrane Water Electrolysis. Electrochem. Commun. 2020, 110, 106640 10.1016/j.elecom.2019.106640.

[ref364] Klingele M.; Breitwieser M.; Zengerle R.; Thiele S. Direct Deposition of Proton Exchange Membranes Enabling High Performance Hydrogen Fuel Cells. J. Mater. Chem. A 2015, 3, 11239–11245. 10.1039/C5TA01341K.

[ref365] Karaca A.; Galkina I.; Sohn Y. J.; Wippermann K.; Scheepers F.; Glusen A.; Shviro M.; Muller M.; Carmo M.; Stolten D. Self-Standing, Ultrasonic Spray-Deposited Membranes for Fuel Cells. Membranes 2023, 13, 522 10.3390/membranes13050522. 37233583 PMC10221716

[ref366] Breitwieser M.; Klose C.; Klingele M.; Hartmann A.; Erben J.; Cho H.; Kerres J.; Zengerle R.; Thiele S. Simple Fabrication of 12 μm Thin Nanocomposite Fuel Cell Membranes by Direct Electrospinning and Printing. J. Power Sources 2017, 337, 137–144. 10.1016/j.jpowsour.2016.10.094.

[ref367] Fu S.-Q.; Hu B.; Ge J.-H.; Zhu M.-Z.; Huang P.-P.; Xue B.; Li N.; Liu P.-N. Application of Ionized Intrinsic Microporous Poly(phenyl-alkane)s as Alkaline Ionomers for Anion Exchange Membrane Water Electrolyzers. Macromolecules 2023, 56, 6037–6050. 10.1021/acs.macromol.3c00739.

[ref368] Hu C.; Kang H. W.; Jung S. W.; Liu M. L.; Lee Y. J.; Park J. H.; Kang N. Y.; Kim M. G.; Yoo S. J.; Park C. H.; Lee Y. M. High Free Volume Polyelectrolytes for Anion Exchange Membrane Water Electrolyzers with a Current Density of 13.39 A cm^–2^ and a Durability of 1000 h. Adv. Sci. 2024, 11, e2306988 10.1002/advs.202306988. PMC10837377 38044283

[ref369] Lehmann M.; Leonard D.; Zheng J.; He L.; Tang X.; Chen X. C.; Lim K. H.; Maurya S.; Kim Y. S.; Saito T. Quaternized Polynorbornene Random Copolymers for Fuel Cell Devices. ACS Appl. Energy Mater. 2023, 6, 1822–1833. 10.1021/acsaem.2c03682.

[ref370] Donnan F. G. The Theory of Membrane Equilibria. Chem. Rev. 1924, 1, 73–90. 10.1021/cr60001a003.

[ref371] Sarkar S.; SenGupta A. K.; Prakash P. The Donnan Membrane Principle: Opportunities for Sustainable Engineered Processes and Materials. Environ. Sci. Technol. 2010, 44, 1161–1166. 10.1021/es9024029. 20092307

[ref372] Kushner D. I.; Crothers A. R.; Kusoglu A.; Weber A. Z. Transport Phenomena in Flow Battery Ion-Conducting Membranes. Curr. Opin. Electrochem. 2020, 21, 132–139. 10.1016/j.coelec.2020.01.010.

[ref373] Kamcev J.; Paul D. R.; Freeman B. D. Ion Activity Coefficients in Ion Exchange Polymers: Applicability of Manning’s Counterion Condensation Theory. Macromolecules 2015, 48, 8011–8024. 10.1021/acs.macromol.5b01654.

[ref374] Wedege K.; Drazevic E.; Konya D.; Bentien A. Organic Redox Species in Aqueous Flow Batteries: Redox Potentials, Chemical Stability and Solubility. Sci. Rep. 2016, 6, 39101 10.1038/srep39101. 27966605 PMC5155426

[ref375] Becker H.; Berger W.; Domschke G.; Fanghänel E.; Faust J.; Fischer M.; Gentz F.; Gewald K.; Gluch R.; Mayer R.; Müller K.; Pavel D.; Schmidt H.; Schollberg K.; Schwetlick K.; Seiler E.; Zeppenfeld G. Organikum, 5th ed.; VEB Deutscher Verlag der Wissenschaften: Berlin, 1976.

